# Chromosomal fusions trigger rediploidization of autopolyploid genomes

**DOI:** 10.1038/s41586-026-10439-1

**Published:** 2026-04-22

**Authors:** Chuanshuai Xie, Zitu Ma, Chaowei Zhou, Kexin Ma, Haoyu Wang, Jiahong Wu, Yan Zhou, Yongrui Lu, Da Ji, Xuedie Gu, He Gao, Junting Li, Suxing Fu, Weiqiang Li, Zhaofang Han, Shijun Xiao, Fei Liu, Benhe Zeng, Sheng’ao Chen, Jiangong Niu, Tao Zhang, Jian Shen, Chunna Liu, Jing Luo, Daniel J. Macqueen, Axel Meyer, Haiping Liu, Luohao Xu

**Affiliations:** 1 MOE Key Laboratory of Freshwater Fish Reproduction and Development, College of Fisheries, School of Life Sciences, Integrative Science Center of Germplasm Creation in Western China (Chongqing) Science City, Southwest University, Chongqing, China; 2 Bio-X Center for Interdisciplinary Innovation, School of Ecology and Environment, School of Life Sciences, Yunnan University, Kunming, China; 3 College of Mycology, Jilin Agricultural University, Jilin, China; 4 Institute of Aquatic Sciences, College of Agriculture and Animal Husbandry Xizang Autonomous Region, Xizang Autonomous Region, China; 5 College of Life Science and Technology, Tarim University, Xinjiang Uygur Autonomous Region, China; 6 Xinjiang Uygur Autonomous Region Aquatic Development Center, Xinjiang Uygur Autonomous Region, China; 7 China Institute of Water Resources and Hydropower Research, Beijing, China; 8 Southwest United Graduate School, Yunnan University, Kunming, China; 9 The Roslin Institute and Royal (Dick) School of Veterinary Studies, University of Edinburgh, Edinburgh, UK; 10 Department of Biology, University of Konstanz, Konstanz, Germany; 11 Museum of Comparative Zoology, Harvard University, Cambridge, MA USA; 12 State Key Laboratory of Tropical Oceanography, South China Sea Institute of Oceanology, Chinese Academy of Sciences, Guangzhou, China

**Keywords:** Molecular evolution, Chromosomes, Evolutionary genetics, Polyploidy

## Abstract

The ancestor of all vertebrates is thought to have undergone autopolyploid whole-genome duplication (WGD)^1,2^, doubling the genetic raw material for evolutionary diversification^3–5^. However, we still do not understand the first steps of rediploidization that followed, required for the emergence and divergence of duplicated genes (ohnologues) created by WGD^6,7^. Consequently, how the functional potential created by autopolyploidy becomes realized during evolution remains unclear. Snow carps (Schizothoracine) have a history of recent WGDs and evolved high-altitude adaptations^8–10^, making these fish a particularly suitable system to study the early stages and consequences of rediploidization. Here genomic data from all snow carp genera reveal their autopolyploid origin, including tetraploids, hexaploids and one icosaploid (20*n*). We present haplotype-resolved genomes for two snow carp species (*Schizopygopsis younghusbandi* and *Schizothorax curvilabiatus*) from divergent lineages, revealing a single ancestral autotetraploidy event. Comparative genomic, meiotic pairing and allele composition analyses indicate that unbalanced chromosome fusions were responsible for the transition from tetrasomic to disomic inheritance, creating genomic regions harbouring diploid ohnologue pairs, with non-rearranged chromosomes remaining tetraploid. This study suggests that this mechanism initiated rediploidization and documents its early chromosomal and genomic consequences. It starts at chromosome fusion sites and expands outwards towards chromosomal arms, a process that remained incomplete post-speciation, leading to a mixture of ancestral and lineage-specific ohnologue divergence on highly syntenic chromosomes.

## Abstract (web summary)

Genomic data from snow carps reveal that the fusion of chromosomes initiates rediploidization following whole-genome duplication.

## Main

WGD through polyploidization has occurred at many stages of eukaryotic evolution, with potentially profound implications for lineage diversification^3–5^. Over 50 years ago, Ohno argued that WGD occurred during early vertebrate evolution, providing new genetic material for saltatory evolution via a burst of gene duplication^1,2^. WGD creates duplicated gene copies (ohnologues) across the genome, each with the potential to evolve new functions and expression phenotypes^6,7^. WGD is thought to have shaped the evolution of complexity in vertebrates, a group that underwent WGD ancestral to gnathostomes and cyclostomes, along with lineage-specific WGDs at the base of each of these lineages^11,12^.

WGD can occur within species (autopolyploidy) or following hybridization of different species (allopolyploidy). The former duplicates identical chromosome sets, whereas the latter doubles the chromosome sets merged from distinct parent species. In all cases, WGD is followed by rediploidization, where the duplicated chromosomes return to a more stable diploid state^7,13^. Studies of WGD events ancestral to all teleost^14^, salmonid^15–17^ and acipenseriform^18,19^ fish revealed that rediploidization occurred asynchronously across the genome. Consequently, although some regions reverted to disomic inheritance, others remained tetrasomic (maintaining four alleles at affected loci) for tens of millions of years post-WGD. Asynchronous rediploidization also challenges inferences on the timing and number of WGD events during evolution, due to the potentially incorrect assumption that WGD is closely temporally coupled to ohnologue divergence^18^. Although the importance of rediploidization is recognized, its early stages remain unknown, as the WGD events studied to date are very ancient.

Snow carps (Schizothoracinae, Cyprinidae) offer an ideal system to study the early stages of vertebrate rediploidization^8^. The group originated 10–30 million years ago (Ma) during the dramatic uplift and climatic upheavals of the Qinghai–Tibetan Plateau^9,10^. The group is polyploid (4–20*n*^20,21^), comprising more than 100 species from 11 genera assigned to three groups — ‘non-specialized’ (sometimes previously designated ‘basal’), ‘specialized’ and ‘highly specialized’ — on the basis of unique phenotypic adaptations^9,22^ (Supplementary Fig. 1). Here we report two haplotype-resolved snow carp genomes, and show that all snow carps share a single ancestral autotetraploidy. Although much of the genome remains tetrasomic with four randomly pairing chromosomes, rediploidization occurred following chromosomal fusions, progressing outwards along the chromosome. Our findings offer a model for the first stages of autopolyploid rediploidization, with potentially general implications for understanding vertebrate diversification.

## Genomic data confirm autopolyploidy

**Fig. 1:**
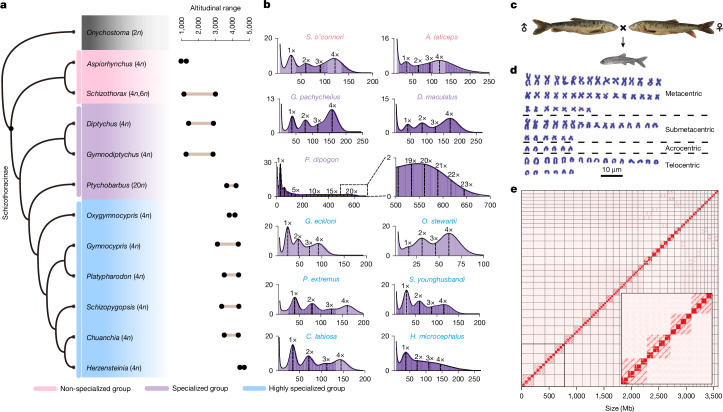
All snow carp genera are autopolyploid. **a**, Mitogenome phylogeny of all 11 snow carp genera, grouped by three classes defined by morphological specialization. All nodes exhibited bootstrap values greater than 70. The 25th and 75th percentile of the altitudinal range^47^ are also shown (right). **b**, *K*-mer frequency distributions of 11 species. In *P. dipogon*, 1× represents the position of the first peak, and 2–20× are calculated as multiples of the 1× peak. The *x* axis represents *k*-mer depth (number of occurrences), and the *y* axis shows the number of distinct *k*-mers at each depth. **c**, The parent–offspring trio of *S. younghusbandi* (photo credit: H.G.). **d**, The karyotyping of *S.* *younghusbandi* reveals a tetraploid number of 4*n* = 90 chromosomes, comprising metacentric (40), submetacentric (22), acrocentric (6) and telocentric (22) chromosomes. **e**, Hi-C heatmap of *S. younghusbandi*, with homologous chromosomes involved in fusions placed at the end.

We generated 1.5 TB short-read sequencing data spanning all 11 snow carp genera (Fig. 1a and Supplementary Table 1). *K*-mer frequency distributions revealed clear peaks at 1×, 2× and 4× depths for all species (Fig. 1b) except *Platypharodon dipogon* and *Schizothorax wangchiachii*, which showed respective peaks at 20× depth (Fig. 1b) and 6× depth (Supplementary Fig. 2), confirming icosaploidy (20*n*)^21^ for *P. dipogon* and hexaploidy (6*n*) for *S. wangchiachii*. Estimated haploid genome sizes ranged from 724 Mb to 921 Mb (Supplementary Table 1), consistent with published genome assemblies (Supplementary Table 2). The absence of prominent 2× *k*-mer peaks in most species is consistent with the presence of autotetraploidy in all snow carps (Fig. 1b). A phylogenetic tree using the mitochondrial genomes (Fig. 1a) confirmed relationships observed in past work^23^, capturing the non-specialized, specialized and highly specialized snow carp groups.

## Fully phased genome assembly

Assembling the genomes of young autopolyploids remains challenging, despite recent advances in long-read sequencing technologies, partially because only few unique Hi-C reads can be assigned to contigs of different haplotypes. We initially selected *S. younghusbandi* (highly specialized group), one of the most commercially valuable fishes in the Yarlung Tsangpo River, to produce a haplotype-resolved genome assembly. A parent–offspring trio was established where divergent parental samples were selected (Fig. 1c). The offspring was sequenced to generate more than 100× coverage PacBio HiFi reads, more than 50× coverage Oxford Nanopore Technologies (ONT) ultra-long reads, and more than 250× coverage for Hi-C reads (Supplementary Table 3). Each parent was also sequenced at more than 100× coverage with short-read data (Supplementary Table 3).

**Extended Data Fig. 1:**
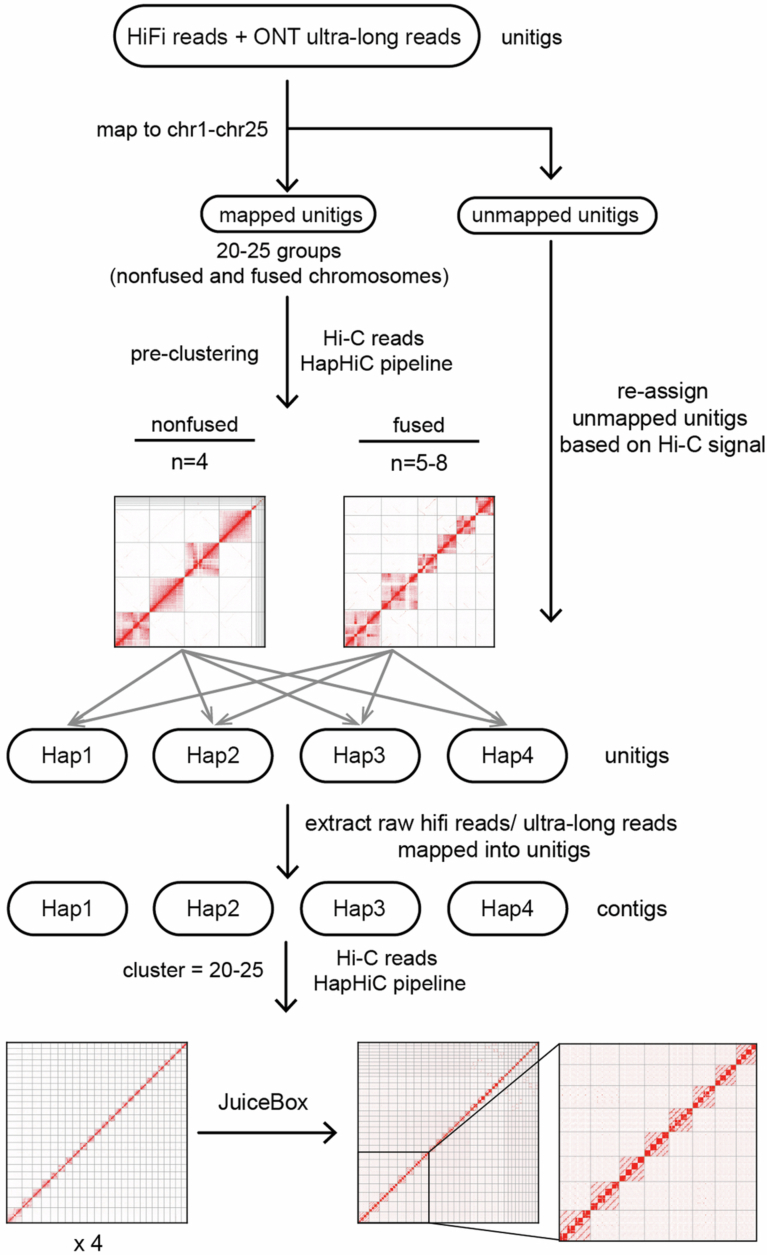
Workflow of read-binning assembly. Unitigs generated from Hifiasm are divided into 20-25 groups based on detected fusions, and clustering is performed using an expected chromosome number of 4–6, also informed by fusion signals. The clustered unitigs are manually assigned to four haplotypes (hap1-hap4). Long reads (HiFi and ONT) are subsequently extracted according to the inferred relationships between unitigs and haplotypes. Finally, Hifiasm and Hap-HiC are re-run to perform a standard scaffolding pipeline.

To reduce assembly complexity, we used a read-binning and a reassembly approach (Extended Data Fig. 1; Methods) as done for the polyploid sugar cane^24^. In brief, de novo assembly of long reads generated 3.67-Gb unitigs that were grouped into 25 clusters by chromosome homology. The raw reads for each four haplotypes of a chromosome were re-extracted for assembly (Supplementary Table 4), followed by Hi-C scaffolding. This independent Hi-C scaffolding per haplotype improved the utilization of Hi-C data (unique Hi-C reads increased from 1% to 12%). The final haplotype-resolved assembly consisted of 90 chromosomes containing 373 gaps (Supplementary Table 4), consistent with the observed karyotype (Fig. 1d,e).

To assess the effectiveness of this approach, we generated another assembly using the standard trio-binning strategy (Supplementary Fig. 3; Methods), leading to the assembly of 45 maternal chromosomes and 45 paternal chromosomes, respectively (Supplementary Table 5). The genomes obtained through both strategies were highly consistent, without major switch errors of maternal and paternal chromosomes (Supplementary Fig. 4). The genome obtained through read binning showed comparable completeness and accuracy (BUSCO value of 99.1% versus 99.0%; quality value score of 57.8 versus 57.9) and improved contiguity (373 gaps versus 429 gaps). Thus, our read-binning approach is both efficient and accurate for assembling autopolyploid genomes. Approximately 55% of the genome comprises repeat sequences (Supplementary Table 6 and Supplementary Fig. 5) and 100,212 genes were annotated for downstream analyses.

## Unbalanced chromosomal fusions

**Extended Data Fig. 2:**
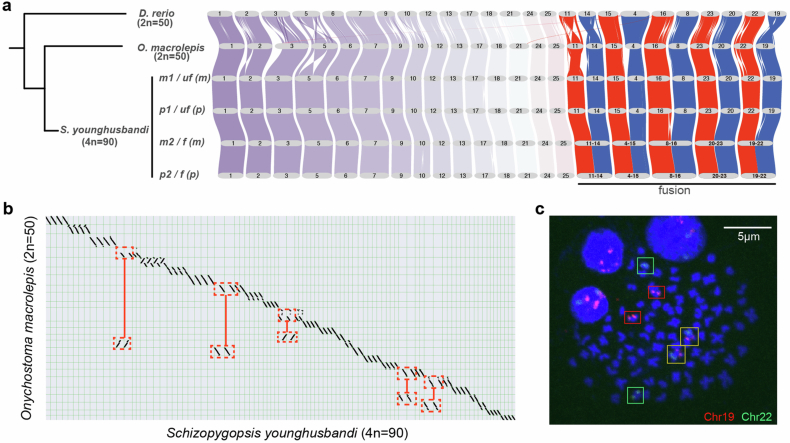
Five pairs of unbalanced chromosomal fusions in *S. younghusbandi.* (a) Genomic synteny among *S. younghusbandi*, *O. macrolepis* and *D. rerio*. The right panel shows the genomic synteny, while the left panel displays the phylogenetic relationships (topology only; branch lengths are omitted). The homologous links of the two chromosomes involved in the fusion were labeled in red and blue, respectively, to visualize whether mixing has occurred. (b) Dot plot shows the collinearity between chromosomes of *S. younghusbandi* and *O. macrolepis*. In *S. younghusbandi*, each of 10 chromosomes corresponds to two chromosomes in *O. macrolepis*, marked in orange. (c) Fluorescence in situ hybridization (FISH) experiments showing the localization of chr19 and chr22 probes. Red signals represent chr19, green signals represent chr22, and yellow signals show the co-localized (fused) chromosomes. The experiment was repeated twice with similar results. Chromosomes were stained with DAPI.

Although the haploid chromosome number of snow carp is *n* = 25 (ref. ^25^), the assembled and cytogenetically observed chromosome number of *S. younghusbandi* is 90 (Fig. 1d,e) instead of the expected 100 (4*n* = 25 × 4), suggesting either chromosomal fusions or losses after polyploidization. Genomic synteny comparison with the outgroup species *Onychostoma macrolepis*^26^ revealed chromosome fusions for five chromosomal pairs (Extended Data Fig. 2a,b), supporting the hypothesis that chromosomal fusions, not losses, were responsible for chromosome number reduction^27^.

**Fig. 2:**
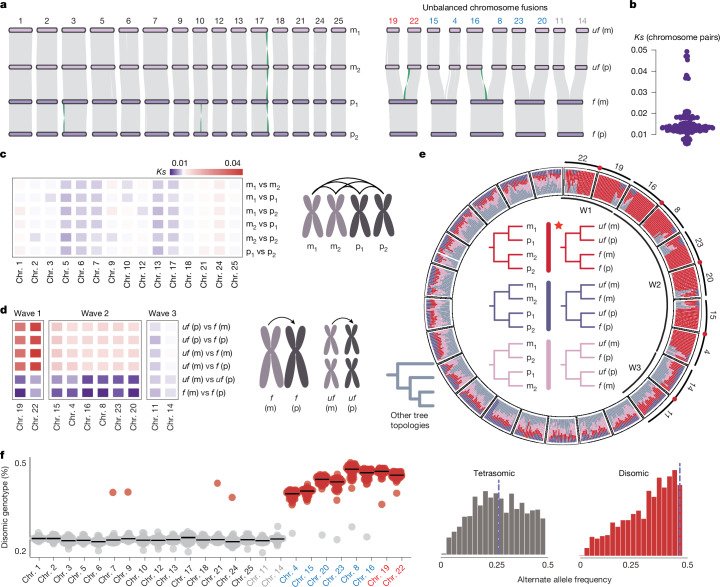
Three waves of rediploidization in the *S. younghusbandi* genome. **a**, Synteny between four chromosome-scale haplotypes of *S. younghusbandi*, including 15 unfused quartets and 10 fused quartets. The chromosome ID colours represent three different waves of rediploidization. Inversions are marked in green. **b**, Average sequence divergence (*Ks*) among all pairwise homologous and putative homoeologous (rediploidized) chromosome pairs. **c**,**d**, Heatmaps showing mean *Ks* values between tetrasomic (**c**) and disomic (**d**) chromosome pairs. A model of chromosomal pairing leading to tetrasomic (that is, non-preferential pairing) and disomic (that is, preferential bivalent pairing) inheritance pattern is also shown (right). Chr., chromosome. **e**, Distribution of phylogenetic topologies of four haplotypes across the genome. Each chromosome was partitioned into 20 bins, and phylogenetic trees were reconstructed within each bin based on alignments of 30-kb windows. The bar plot illustrates the proportion of each topology within every bin. The red dots indicating the positions of fusion sites. W1–W3 denote the three waves. The topology expected to result from rediploidization is marked with a red asterisk. **f**, Ratio of disomic genotype to alternate allele frequency distribution based on resequencing data of 48 *S. younghusbandi* individuals. Each point represents one individual (*n* = 48 resequenced samples), and the black centre line denotes the median. The alternate allele frequency refers to the proportion of allele depth of alternate alleles to the total depth, with a peak around 0.5 for disomic and 0.25 for tetrasomic inheritance (vertical dashed line). Red, blue and grey indicate waves 1, 2 and 3, respectively.

As young autotetraploids maintain tetrasomic inheritance, the four homologous chromosomes are expected to pair non-preferentially during meiosis and share homogenized sequences due to four-way recombination. The five fusions identified above, however, involved only two of the four homologous chromosomes, with the other two unfused (Fig. 2a). Fluorescence in situ hybridization (FISH) experiments were conducted to verify the unbalanced chromosomal fusions (Extended Data Fig. 2c). These unbalanced chromosomal changes implied that the initial tetraploid chromosomes diverged into fused (*f*) and unfused (*uf*) copies with different evolutionary trajectories. We therefore denoted those chromosomes as *f* (m), *f* (p), *uf* (m) and *uf* (p), where ‘m’ and ‘p’ indicate the parental origins (Fig. 2a). Other quartets without fusions were denoted as m_1_m_2_p_1_p_2_, where ‘1’ and ‘2’ were randomly assigned. These unbalanced chromosomal fusions also raised the possibility that the genome of *S. younghusbandi* is in the process of rediploidization.

## Onset of asynchronous rediploidization

To investigate whether and to what extent rediploidization has occurred, we compared sequence divergence (*Ks* values) among homologous (and potentially homoeologous) chromosomal quartets (Fig. 2b and Supplementary Table 7). Out of the 25 chromosomal quartets, 15 unfused quartets show limited divergence in all four combinations (*Ks* values close to 0.01), suggesting they are homologous, pairing non-preferentially during meiosis, producing tetrasomic inheritance (Fig. 2c). For the 10 quartets where unbalanced chromosomal fusions occurred (Fig. 2a), higher *Ks* values were observed between any combination of fused versus unfused chromosomes, whereas divergence of haplotype pairs within the fused or unfused chromosome sets was negligible (Fig. 2d). In addition, analysis of 27,049 phylogenetic trees generated along the genome revealed a predominant topology ((*uf*, *uf*), (*f*, *f*)) on the fused quartets, in contrast to a uniform distribution of multiple topologies on the unfused quartets (Fig. 2e). This supports a model in which the unbalanced fusions suppressed non-preferential pairing during meiosis and promoted preferential bivalent pairing of the fused or unfused chromosome sets, leading to rediploidization and ohnologue divergence. Of note, these 10 quartets show evidence for varying levels of rediploidization and hence sequence divergence. The chromosome 19 and chromosome 22 quartets show the highest *Ks* values (Fig. 2d), which may represent the first ‘wave’ of rediploidization within the genome (hereafter, wave 1). Six other quartets showed moderate *Ks* values (less than the chromosome 19 and chromosome 22 quartets; wave 2), whereas the chromosome 11 and chromosome 14 quartets exhibit barely elevated *Ks* values compared with unfused quartets, implying that rediploidization may be in its very early stages, with limited ohnologue divergence (wave 3). This result supports a model in which the chromosomal fusions occurred at different times in evolution, leading to different absolute timing of rediploidization among the fused chromosomes.

Concurrently, we noted that chromosomes that had reverted to disomic inheritance exhibited limited heterozygosity (1.1%), which was significantly lower than that of chromosomes still under tetrasomic inheritance (1.8%, two-sided Mann–Whitney *U*-test *P* < 0.01; Supplementary Table 8). The *Ka*/*Ks* values between maternal and paternal in fused quartet (*Ka/Ks* median = 0.095) are significantly lower than that in unfused quartets (*Ka/Ks* median = 0.137, *P* < 0.01; Supplementary Fig. 6). This suggests more efficient purifying selection under disomic inheritance^28^. Furthermore, compared with tetrasomic inheritance, genetic drift and inbreeding are expected to exert greater effects in disomic systems^29^. These factors may collectively contribute to the reduced heterozygosity observed among disomic chromosomes.

To validate the co-existence of disomic and tetrasomic inheritance within the same genome, we used an allele-based method^30,31^ to compare the proportion of different tetraploid genotypes (AAAA, AAAa, AAaa, Aaaa and aaaa) among the chromosomes. The fused chromosomal quartets, except for chromosome 11 and chromosome 14, have significantly more disomic genotypes (AAaa) and less tetrasomic genotypes (AAAa and Aaaa) than the unfused quartets (*P* < 0.05; Supplementary Fig. 7). Consistently, in wave 1 and wave 2 quartets, the alternative allele depth ratio displayed a prominent peak at 0.5, whereas the unfused quartets exhibited a peak at 0.25 (Supplementary Fig. 7). We further analysed resequencing data from 48 additional *S. younghusbandi* individuals^32^, and consistently reproduced this pattern (Fig. 2f), indicating that the inferred state of asynchronous rediploidization is stable in *S. younghusbandi*.

## Rediploidization started at fusion sites

**Fig. 3:**
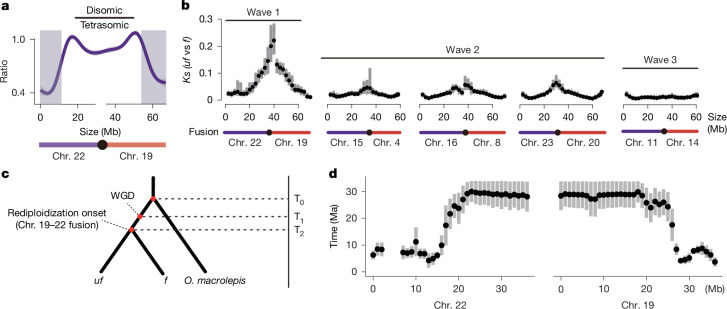
Rediploidization initiates at chromosomal fusion sites. **a**, The *y* axis represents the ratio of disomic (AAaa) to tetrasomic (AAAa and Aaaa) genotypes. The lines indicate fitted trends and the shaded areas represent 95% confidence intervals. The two ends of the fused chromosomes 19–22 remain tetrasomic. **b**, Distribution of *Ks* values (*uf* versus *f*) along the fused chromosomes. The coordinates of the *x* axis are based on the *f* (m) chromosomes. Points represent the median, and the error bars denote the interquartile range. The sample size for each point is the total number of genes within the corresponding 5-Mb window. **c**, Evolutionary timeline of *S. younghusbandi* depicting the divergence from *O. macrolepis* (T0), followed by WGD (T1) and the subsequent onset of rediploidization (T2). **d**, Divergence time estimates for chromosomes 22 and 19 based on an independent rate clock model in 2-Mb windows. The bars represent the 95% highest posterior density (HPD) intervals, and the points indicate the median values.

On the fused chromosomes, we found that the disomic genotypes are primarily distributed around the fusion sites, whereas the chromosome arms tend to maintain tetrasomic genotypes (Fig. 3a and Supplementary Fig. 8). Our phylogenomic analysis also supported this distribution with respect to the location of the ((*uf*, *uf*), (*f, f*)) topology (Fig. 2e). To further study the occurrence and timing of rediploidization, we used 5-Mb sliding windows to estimate divergence levels between the fused and the unfused chromosomes (Fig. 3b). The chromosomal centres exhibited the highest divergence levels (Fig. 3b), suggesting that they began diverging earlier than other chromosomal regions. Similarly, a greater degree of divergence in protein sequences is observed near the fusion sites (Supplementary Fig. 8). These findings provide strong evidence that rediploidization depends on proximity to chromosomal fusion sites, and that snow carps are in the early stages of a highly asynchronous rediploidization process.

The divergence time estimated at the fusion sites would be expected to mark the onset of rediploidization. However, the protracted and asynchronous nature of the rediploidization process implies an intervening period of tetraploid inheritance of unknown duration following the initial WGD (Fig. 3c). Therefore, the time of the first rediploidization within the genome, denoted as T_2_, can be regarded as a lower bound for the timing of WGD (T_1_), whereas the upper bound is constrained by the divergence time of the diploid common ancestors (T_0,_ approximately 30 Ma)^33^ (Fig. 3c). Using an independent rate molecular clock model, we estimated divergence times associated with the chromosome 19–chromosome 22 fusion event. The peak divergence time inferred from the fusion point of chromosome 19–chromosome 22 (wave 1) was approximately 30 Ma (Fig. 3d). Similarly, our estimates indicated that the wave 2 rediploidization started 10–20 Ma (Supplementary Fig. 9). The tetrasomic inheritance observed in all telomeric regions (distal to the fusion sites) implies that homologous recombination is maintained in those regions.

## Chromosome behaviour

**Fig. 4:**
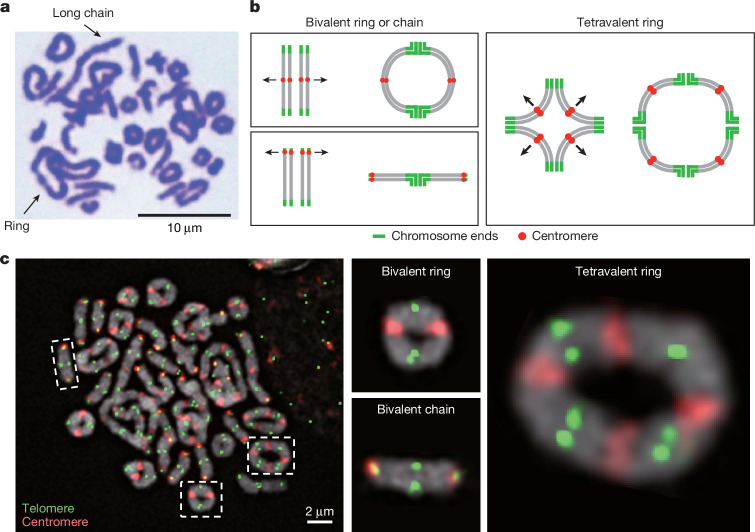
Chromosome behaviour in meiotic metaphase I. **a**, A karyotype of meiotic metaphase I cells of the testis. The experiment was repeated six times with similar results; additional results are shown in Supplementary Fig. 10. **b**, Schematic of the formation of ring and chain structures. The left side within a box is a schematic of chromosome pairing during the pachytene stage, and the right side is the shape after the metaphase is pulled apart. **c**, Fluorescence in situ hybridization of centromeres and telomeres on meiotic metaphase I chromosomes. The right panels show examples of a bivalent ring, a bivalent chain and a tetravalent ring. The experiment was repeated four times with similar results; additional results are shown in Extended Data Fig. 5.

**Extended Data Fig. 3:**
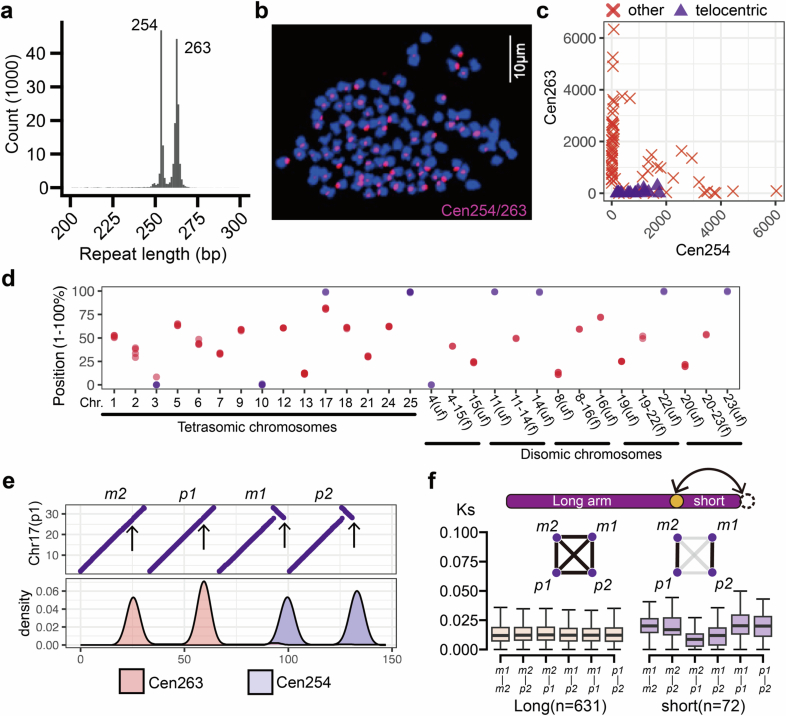
Centromere identification and repositioning. (a) Distribution of centromere monomer lengths in *S. younghusbandi*. (b) FISH of centromeric repeats on metaphase chromosomes. Centromeric probes are marked in red, and chromosomes are stained with DAPI. The experiment was repeated three times with similar results. (c) The location of centromeres on the 90 chromosomes. Each dot represents the location of a centromere, blue represents the telocentric chromosomes, and red represents the others. (d) The composition of Cen254 and Cen263 in different chromosomes. The acrocentric or telocentric chromosomes are marked with a purple triangle, and the other chromosomes are marked in red. (e) The inversion and centromere composition of Chr17. Because 1 and 2 are randomly assigned, m1 and p2 exhibit consistent collinearity, as do m2 and p1. (f) Inheritance pattern on Chr17. The *Ks* values among haplotypes were calculated in short arm and long arm, respectively, where the short arm shows a disomic pattern, consistent with the pattern in rediploidization. In the short arm, the two haplotypes carrying the inversion exhibit lower *Ks* values, suggesting preferential pairing. In the long arm, the *Ks* values between paired haplotypes are similar, indicating non-preferential pairing. The box plots display the median (center line), the interquartile range (box), and the data range within 1.5×IQR (whiskers).

To further study the genomic reconstruction of snow carp rediploidization at the cytogenetic level, we collected metaphase I meiotic chromosomes of *S. younghusbandi* (Fig. 4a and Supplementary Fig. 10). We observed both large ring-like structures and chain-like structure that are hallmarks of autopolyploids^34^. Previous studies have suggested that ring-like pairings are probably formed by metacentric chromosomes, whereas chain-like pairings are formed by telocentric chromosomes^34^ (Fig. 4b). To test this hypothesis, we annotated the centromere sequence of *S. younghusbandi* (Extended Data Fig. 3a) and found that telocentric chromosomes tend to use a 263-bp monomer (Cen263) as the centromeric repeat, whereas other chromosomes tend to use 254-bp monomer (Cen254; Extended Data Fig. 3b,c and Supplementary Table 9), although Cen263 and Cen254 are homologous (Supplementary Fig. 11). Of the 90 chromosomes, 23 (26%) are telocentric (Extended Data Fig. 3d), whereas the rest (67, 74%) are metacentric, submetacentric and acrocentric chromosomes, explaining the coexistence of both ring and chain shapes.

**Extended Data Fig. 4:**
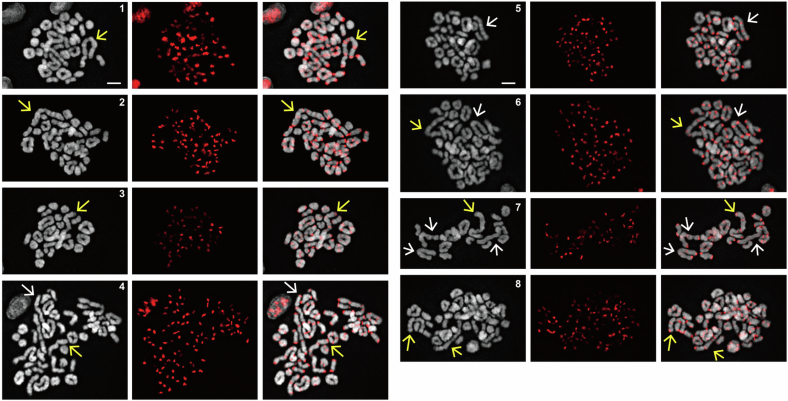
Centromere FISH showing chromosome pairing behavior during meiosis I. Long chain configurations are observed in eight representative fields, with yellow arrows indicating hexavalent chain configurations and white arrows pointing to tetravalent chain configurations. Chromosomes are stained with DAPI, and centromeres are labeled in red. Scale bar: 2μm. The experiment was repeated at least five times with similar results.

**Extended Data Fig. 5:**
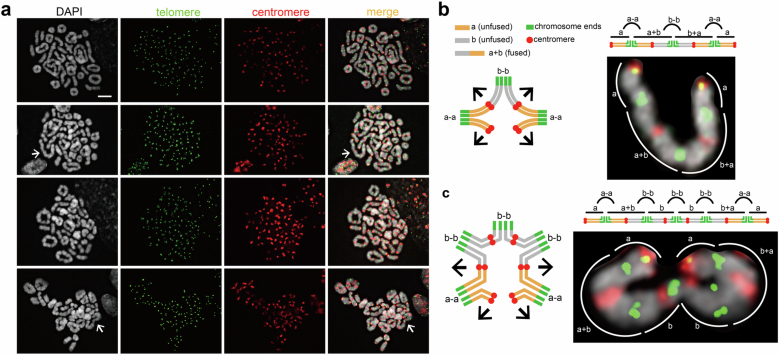
Multivalent chain formation through pairing of fused and unfused chromosomes, revealed by FISH. (a) Meiotic metaphase I karyotype in four representative fields, showing telomeres (green), centromeres (red), and DAPI-stained chromosomes (blue). Scale bar: 2μm. Arrowheads indicate tetravalent or hexavalent chains, with close-up views shown on the right. (b) Schematic diagram of a tetravalent chain, formed by a fused chromosome pairing with one pair of unfused chromosomes. (c) Schematic diagram of a hexavalent chain, formed by a fused chromosome pairing with two pairs of unfused chromosomes. The unfused chromosomes are labeled a and b, and the resulting fused chromosome is designated a + b. The experiment was repeated four times with similar results.

To further verify multivalent pairing, we visualized chromosomes at metaphase I by using centromeric and telomeric probes. The presence of four centromeric foci and four telomeric foci within ring-shaped configurations supports quadrivalent pairing (Fig. 4c). In addition, ring–chain-shaped bivalent pairing structures were also observed, and as expected, their distinction lies in the positions of the centromeres (Fig. 4b). We found that the long-chain configurations consist of four or six centromeric foci (Extended Data Fig. 4), which may be formed by the pairing of fused chromosomes with one or two pairs of unfused chromosomes (Extended Data Fig. 5).

We also detected two centromeric repositioning events, as demonstrated in chromosome 3 and chromosome 17 (Supplementary Fig. 11). In chromosome 17, the centromeres in two chromosomes are approximately 5 Mb away from the centromeres in the other two chromosomes, probably caused by a centromeric inversion (Extended Data Fig. 3e). The newly formed submetacentric chromosome used Cen263 rather than Cen254 (Extended Data Fig. 3e), suggesting that centromeric repositioning led to turnover of centromeric repeats. In the short arm of chromosome 17 involving a centromeric inversion, a rediploidized pattern might be indicative of disomic inheritance, whereas the long arm showed a tetrasomic inheritance pattern (Extended Data Fig. 3f). This rediploidization pattern is not observed in other acrocentric chromosomes without centromeric inversions (Supplementary Fig. 12), indicating an association between rediploidization and centromeric inversions.

## Asymmetric ohnologue evolution

**Extended Data Fig. 6:**
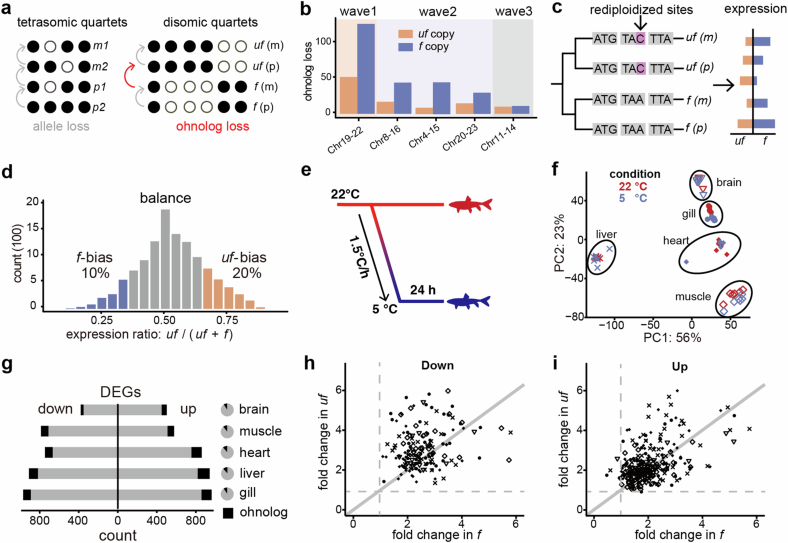
Biased gene loss and transcriptional activity in *uf* copies. (a) Schematic diagram of gens loss caused by allele loss or ohnolog loss. Each hollow dots indicates a gene loss. Gray arrows indicate allele relationships, while red arrows indicate ohnolog relationships. (b) Ohnolog loss occurs significantly more often in *f* copies than in *uf* copies (a two-sided chi-square test against the null hypothesis of random loss, p = 0.0001). (c) Inference of relative ohnolog expression using rediploidized sites. Only genes with at least three rediploidized sites were included. (d) Ohnologs are more often *uf*-biased than *f*-biased, based on RNA-seq from eleven tissues. (e) Cold exposure experimental design. (f) Principal component analysis (PCA) of five tissues from the control and cold-exposed groups. (g) Bar plot showing DEG numbers between control and cold-exposure groups among five tissues. Pie charts showing the proportion of ohnologs among DEGs. Black shading represents ohnologs, while gray shading represents non-ohnologs. (h,i) Fold changes in *uf* and *f* ohnolog expression after cold treatment (Down/Up: down/up-regulated upon cold treatment). Fold change was calculated from TPM values and averaged across biological replicates. Ohnologs with fewer than three biological replicates (lacking rediploidized sites) were excluded. Only differentially expressed ohnologs are shown. Point shape denotes tissue type, consistent with panel (f).

Rediploidization creates ohnologue pairs on different chromosomes that are free to diverge and undergo specialization, neofunctionalization, subfunctionalization or loss. We initially compared the inferred rate of gene loss across the *S. younghusbandi* genome. The majority (13,556, 79%) of genes retain four copies (*O. macrolepis*:*S. younghusbandi* = 1:4), whereas a few retain three (1,901, 11%) or two (948, 5.5%) copies (Supplementary Fig. 13). We further focused on ohnologue loss (Extended Data Fig. 6a), referring to genes absent from either the *uf* or the *f* chromosomes in disomic quartets, and identified 360 such events. Also, consistent with their rediploidization level, wave 1 regions of the genome exhibited the highest gene loss proportion, followed by wave 2, with wave 3 showing the lowest (Extended Data Fig. 6b). In the disomic chromosomes, ohnologue gene loss was significantly biased towards fused chromosomes (chi-squared test, *P* < 0.01), accounting for 71.4% (257 of 360) of ohnologue losses (Extended Data Fig. 6b).

Among the retained four-copy genes, 2,449 disomic ohnologue pairs (representing 9.8% of all genes annotated on one haplotype; designated ohnologue *uf* and *f*) were selected based on phylogenetic evidence for rediploidization (Extended Data Fig. 6c). A total of 60,169 rediploidization sites (substitutions fixed between ohnologue pairs in both alleles) were identified within the coding sequences of these ohnologue pairs, enabling the assignment of transcripts derived from the *uf* or *f* ohnologue (Extended Data Fig. 6c). Using RNA sequencing (RNA-seq) data from 11 tissues (Supplementary Table 10), we evaluated the relative expression levels of *uf* versus* f* ohnologue pairs. Approximately 20% of ohnologue pairs exhibited a *uf-*biased pattern, in contrast to only 10% *f-*biased ohnologue pairs (Extended Data Fig. 6d and Supplementary Fig. 14). Consistent with this pattern, the expression level of *uf* was significantly higher than that of *f* (paired Student’s *t*-test, *P* < 0.01), with the strongest difference found for the wave 1 chromosomes (Supplementary Fig. 15). Moreover, we detected 114 ohnologue pairs exhibiting *f* bias in some tissues but *uf* bias in others, suggesting potential functional divergence (Supplementary Table 11). To investigate whether expression divergence of ohnologues has a role in high-altitude adaptation, we subjected *S. younghusbandi* juveniles to 24-h of low-temperature stress (5 °C) and conducted RNA-seq on five tissues (Extended Data Fig. 6e,f). We detected more differentially expressed genes in the gills than in other tissues (Extended Data Fig. 6g and Supplementary Table 12). Although ohnologue pairs were enriched among differentially expressed genes in the liver and heart (odds ratios = 1.23 and 1.21, respectively; Fisher’s exact test, *P* < 0.01), this enrichment was absent for the other three tissues (Supplementary Table 13). Of note, *uf* and *f* genes showed concordant responses to low-temperature stress, such that upregulation or downregulation of a *uf* gene was generally accompanied by upregulation or downregulation of its homologous *f* gene (Extended Data Fig. 6h,i).

## Shared autopolyploidization

**Fig. 5:**
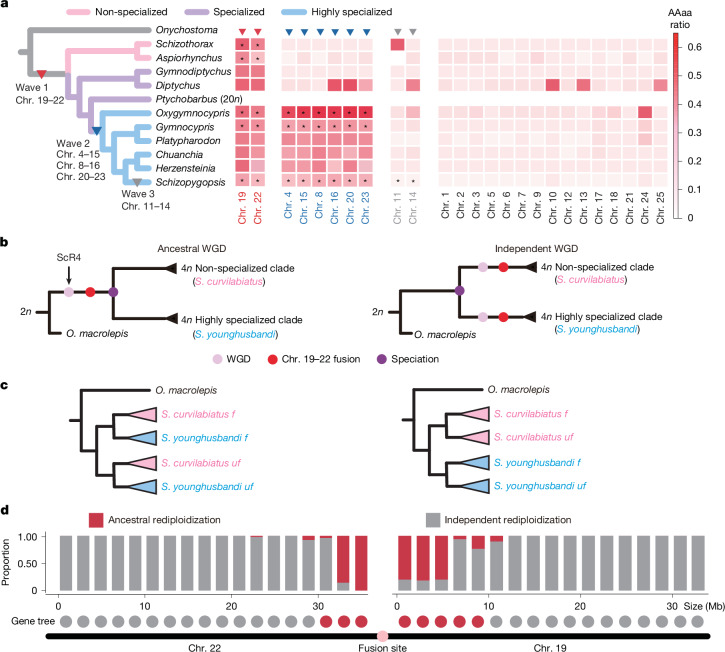
A single ancestral WGD in snow carp. **a**, Proportion of disomic (versus tetrasomic) genotypes across all snow carp genera, with *P. dipogon* (20*n*) and *S. wangchiachii* (6*n*) excluded. The asterisks mark fused chromosomes validated by Hi-C data. The colours of chromosome IDs represent the three different rediploidization waves. **b**, Hypotheses that all snow carps originated from a single WGD (left) or multiple WGDs^10^ (right). **c**, Topologies expected under ancestral (left) and independent or lineage-specific (right) rediploidization. **d**, Distribution of phylogenetic topologies supporting the two rediploidization models along chromosomes 19 and 22. The top bar chart shows the proportions of phylogenetic tree topologies inferred from whole-genome alignment (30-kb windows, summarized in 2-Mb bins). The bottom panel presents the rediploidization model supported by phylogenetic trees built from concatenated protein sequences within 2-Mb bins. Coordinates of the *x* axis are based on the *uf* (m) chromosomes of *S. younghusbandi*.

**Extended Data Fig. 7:**
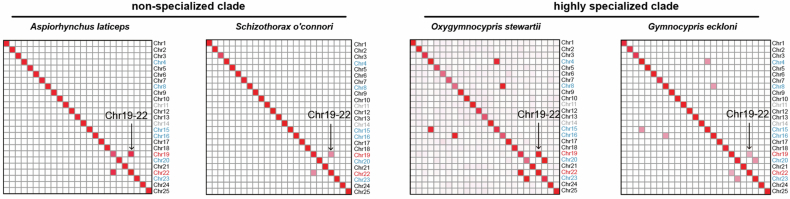
Identification of chromosome fusions based on mapped Hi-C signals. The names of the chromosomes involved in the three waves of rediploidization are marked in different colours. The Hi-C signal are normalized by KR method.

Given the striking association between chromosome fusion, inheritance modes and allelic frequencies (Fig. 3a), we extended our allelic frequency analyses to infer inheritance modes across all snow carp genera (Fig. 5a). We identified a range of 5.2–17.9 million heterozygous biallelic sites across nine additional species to *S. younghusbandi* (Supplementary Table 14). An abundance of disomic genotypes was observed on chromosome 19 and chromosome 22 in all species (Fig. 5a), suggesting that these two chromosomes rediploidized ancestrally. We also detected enrichment of disomic genotypes on six other chromosomes (4, 8, 15, 16, 20 and 23) in the highly specialized lineages (Fig. 5a). These correspond to the wave 2 chromosomes in *S. younghusbandi*, supporting the hypothesis that the same unbalanced fusion events were ancestral to the highly specialized lineages. To test this, we mapped Hi-C data available for four species to calculate interchromosomal interactions, expecting that the fused chromosomes would show a higher frequency of interactions. In agreement with this prediction, we detected strong interaction signals between chromosome 19 and chromosome 22 in all species (Extended Data Fig. 7) and strong contact signals for the three pairs of wave 2 chromosomes in two highly specialized species, corroborating the inferred chromosomal fusions. The chromosomes 11–14 fusion, however, was not observed in other highly specialized species, suggesting that the wave 3 rediploidization was specific to *S. younghusbandi*. The timing of chromosomal fusions at three different points of snow carp phylogeny are therefore probably equivalent to the three rediploidization waves inferred in *S. younghusbandi* (Fig. 5a).

**Extended Data Fig. 8:**
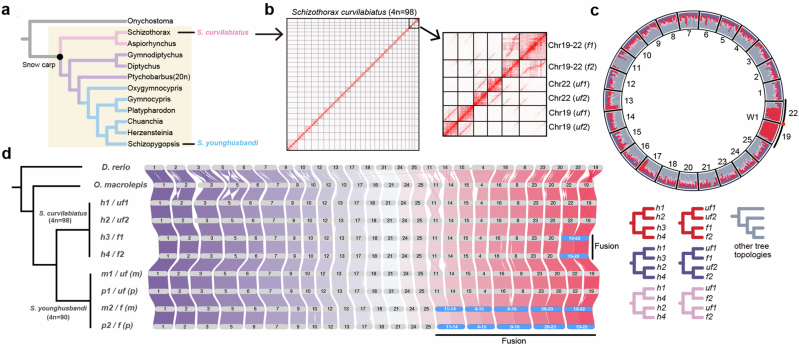
Genome assembly and rediploidization pattern of *S. curvilablatus.* (a) Phylogenetic position of *S. curvilabiatus*. (b) Hi-C contact heatmap of *S. curvilabiatus* (4n = 98), with chromosomes involved in fusion events placed at the end. A collapsed regions of chr19 (*uf*), chr22 (*uf)*, and chr19-22 (*f)* are shown on the right, which was later manually rescued for subsequent analysis. (c) The distribution of phylogenetic topologies of the four haplotypes across the *S. curvilabiatus* genome. Each chromosome was divided into 20 bins, and phylogenetic trees were reconstructed within each bin using 30-kb sliding windows. The bar plots indicate the proportion of each topology within individual bins. Red dots denote the positions of chromosomal fusion site. (d) Genomic synteny among *S. younghusbandi, S. curvilabiatus, O. macrolepis*, and *D. rerio*. The right panel displays genome-wide syntenic relationships, while the left panel shows the corresponding phylogenetic relationships (branch lengths are omitted). Fused chromosomes are highlighted with blue.

The observed coupling of chromosomal fusions and rediploidization supports one ancestral snow carp autopolyploidization event before the chromosomes 19–22 fusion and species diversification (Fig. 5b). To further validate this hypothesis, we generated the haplotype-resolved genome for a non-specialized snow carp, *S. curvilabiatus*, with a chromosome number of 98 (Extended Data Fig. 8a,b and Supplementary Table 15). Comparing *S. younghusbandi* and *S. curvilabiatus* captures the most basal speciation in snow carp evolution (Extended Data Fig. 8a), maximizing scope to detect genomic synapomorphies ancestral to all snow carps. Comparative synteny analysis demonstrate an ancestral fusion of chromosome 19 and chromosome 22 in both species; this chromosome pair exhibits a high degree of rediploidization in *S. curvilabiatus* (wave 1; Extended Data Fig. 8c,d), similar to that for *S. younghusbandi*.

**Extended Data Fig. 9:**
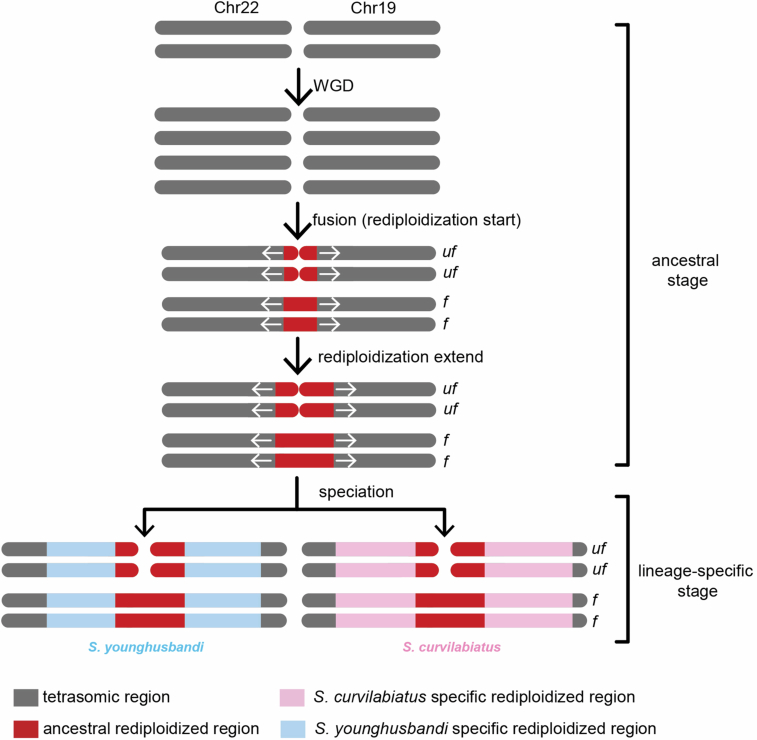
Rediploidization pattern of chr19 and chr22 (wave1). From top to bottom, the schematic shows the evolutionary history of chr19–22. Following WGD, chr19 and chr22 are present in four copies. Subsequently, two copies of chr19 and chr22 underwent chromosomal fusion, which initiated rediploidization spreading from the fusion site toward the chromosomal ends. This ancestral rediploidization state persisted for a period of time, leaving a segment of ancestrally rediploidized regions on chr19 and chr22. A subsequent speciation event then occurred, after which rediploidization proceeded independently in the two descendant lineages, resulting in lineage-specific rediploidized regions.

We next reconstructed phylogenetic trees sampling genomic sequences from whole-genome alignments and concatenated orthologous ohnologues from *S. curvilabiatus* and *S. younghusbandi* along chromosome 19 and chromosome 22. If rediploidization was ancestral, ohnologue pair divergence should have happened in the *S. curvilabiatus–S. younghusbandi* ancestor, captured by orthologous ohnologue pairs located on the same (*f* or *uf*) chromosomes in both species (Fig. 5c). Proximal to the fusion site, our phylogenetic trees captured two ohnologue branches representing either the *f* or *uf* copy from both species, whereas more distal regions of the chromosome showed species-specific *f* + *uf* ohnologue branches (Fig. 5d and Supplementary Fig. 16). In addition to refuting the independent-WGD hypothesis proposed by a recent study^10^, these results indicate that rediploidization along chromosomes 19–22 was incomplete at the point of the earliest snow carp speciation event. Although fusion initiated rediploidization, leading to a small region of shared ancestral ohnologue divergence near the fusion site, the rediploidization process continued in more distal regions in a lineage-specific manner (Extended Data Fig. 9).

## Discussion

Elucidating the genetic mechanisms driving evolutionary changes following autopolyploidy, such as rediploidization, is essential to understanding how the vertebrate lineage diversified^35,36^. Although previous studies on salmonids^15–17^, sturgeons and paddlefish^18^, teleost fish^14,37^ and ancestral vertebrate polyploidy^11,38^ have proposed a model of asynchronous rediploidization, the earliest stage of rediploidization has remained unknown. Leveraging the recent autopolyploidization in snow carps, we demonstrate that chromosomal fusions are the first trigger of rediploidization. It has been proposed that the ancestor of all vertebrates experienced eight to nine chromosomal fusions following 1R^12,39–41^ within a period of approximately 14 million years before 2R, implying that rapid chromosomal fusions may have promoted rediploidization in a manner similar to that for snow carps, pointing to a general mechanism for early rediploidization. It should be noted that not all vertebrate ancestral chromosomes experienced fusions^11,42^, implying that other mechanisms, such as inversions, may also contribute to genome rediploidization, as proposed in salmonids^15^. Moreover, we showed that some downstream consequences of the rediploidization described here mimic subgenome dominance in allopolyploids^43,44^, including biased gene retention and transcriptional activity, meaning that care must be taken when drawing conclusions on allopolyploidy versus autopolyploidy on that sole basis, a point made by others^14^.

Asynchronous rediploidization is known to complicate inference of the timing and number of WGDs, as demonstrated in sturgeons and other lineages^7,16,18,37,45,46^. Our haplotype-resolved genome assemblies for the autotetraploid *S. younghusbandi* and *S. curvilabiatus*, differentiating disomic and tetrasomic regions, reveal that a single ancestral snow carp autopolyploid WGD event was followed by lineage-specific rediploidization. Our study further highlights the importance of identifying the genomic regions where rediploidization initiated earliest, because using other regions with later stages of rediploidization will lead to underestimation of the age of WGDs^16,46^.

Finally, the early stage of asynchronous rediploidization revealed here in snow carp makes these fish an ideal model for further studies on the functional divergence and evolution of ohnologues during adaptive radiation^6^. As rediploidization is still an ongoing process, assembling additional haplotype-resolved genomes from snow carps with varying degrees of rediploidization will provide a valuable window into understanding the functional evolution of young ohnologues derived from recently rediploidized regions.

## Methods

### Sample collection

Wild specimens of *Aspiorhynchus laticeps*, *Gymnodiptychus pachycheilus*, *P. dipogon*, *S. younghusbandi*, *Chuanchia labiosa*, *Platypharodon extremus* and *Herzensteinia microcephalus* were collected from Xinjiang, Gansu and Tibet (Xizang) provinces of China. Except for *S. younghusbandi*, the age and sex of the other sampled individuals were not determined. The captured male and female *S. younghusbandi* were used to obtain the F1 generation through artificial breeding. Genomic DNA from all specimens, including the F1 generation of *S. younghusbandi*, was extracted from the muscle tissue using the cetyltrimethylammonium bromide (CTAB) method. All experiments were approved by the Institutional Animal Care and Use Committee (IACUC) of Southwest University (LAC2024-2-0127).

### Library construction and sequencing

For DNA sequencing, libraries were constructed using the MGIEasy Universal DNA Library Prep Kit V1.0 (1000005250, MGI) following the standard protocol. In brief, 1 μg of genomic DNA was randomly fragmented using a Covaris system. The fragmented DNA was then selected using MGIEasy DNA Clean Beads (1000005279, MGI) to achieve an average size of 200–400 bp. The selected fragments were end repaired, 3′ adenylated and ligated with adapters. The DNA samples were then amplified by PCR, and the products were purified with MGIEasy DNA Clean Beads (1000005279, MGI). The double-stranded PCR products were heat denatured and circularized using the splint oligo sequence from the MGIEasy Circularization Module (1000005260, MGI). The resulting single-stranded circular DNA was used to construct the final library and underwent quality control. The qualified libraries were sequenced on the DNBSEQ-T7RS platform.

For PacBio HiFi sequencing, genomic DNA of *S. younghusbandi* F1 generation was fragmented using the Megaruptor 3 system and concentrated with AMPure PB magnetic beads. SMRTbell libraries were prepared using the Pacific Biosciences SMRTbell Prep Kit 3.0. The libraries were size selected on a PippinHT system for 20-kb molecules, followed by primer annealing and the binding of SMRTbell templates to polymerases using the Revio Polymerase Kit. After primer annealing, sequencing was conducted on the Pacific Biosciences Revio platform.

For ONT ultra-long sequencing, the high-molecular-weight genomic DNA was extracted from the same F1 generation individual. The library was prepared using a Ligation Sequencing Kit (SQK-LSK110, Oxford Nanopore Technologies), and sequencing was performed on the PromethION platform with the flowcell (version r10.4.1).

For the Hi-C library, muscle tissue from the same *S. younghusbandi* individual was crosslinked with 40 ml of 2% formaldehyde solution at room temperature for 15 min. Next, the tissue was ground, washed and resuspended in nuclei isolation buffers to generate a nuclear suspension for further processing. The nuclear suspension was then treated with SDS and Triton X-100, and digested overnight with MboI at 37 °C. Subsequently, the cleaved ends were labelled with biotin-14-dCTP and ligated using DNA ligase to generate chimeric DNA molecules. After reverse crosslinking and biotin removal, the fragments were enriched using streptavidin beads. Finally, adapters were added, and sequencing was performed on the DNBSEQ-T7RS platform.

### Genome survey and mitochondrial phylogeny

Short reads were used to count *k*-mer frequencies by KMC (3.1.1)^48^ with default parameters. The results were subsequently subjected to genomescope 2.0^49^ and Smudgeplot to estimate genome size and ploidy with recommended parameters. We assembled and annotated the mitochondrial genomes from short reads using MitoZ (v3.4)^50^ with assembler megahit–kmers_megahit 59 79 99 119 141 -clade Chordata parameters. We extracted the nucleotide sequences of 13 mitochondrial protein-coding genes and aligned sequences using MAFFT (v7.526)^51^ with default parameters. The alignments were concatenated into a super matrix, and a phylogenetic tree was subsequently constructed using IQ-TREE (v2.0.3)^52^ with the parameters -m TEST, -seqtype DNA, and -bb 10000. In addition, we added the mitochondrial genome of *O. macrolepis* (NC_023799.1) as the outgroup.

### Haplotype-resolved genome assembly

We used two strategies to assemble the genome, including read binning and trio binning strategy. De novo assembly was performed using Hifiasm (0.19.8-r603)^53^ with -ul-cut 30000 -ul-rate 0.12 parameter, based on HiFi and ultra-long ONT data. All unitigs generated by Hihiasm were mapped into the haploid genome^54^ using minimap2 (2.28)^55^ with -cx asm20. Then, Hi-C reads were mapped into unitigs using BWA (0.7.18) mem^54^ with the -5SP parameter. The resulting BAM file was processed according to the HapHiC (v1.0.2) pipeline^56^. We assigned the unitigs into 25 ancestral chromosome groups and simultaneously split the corresponding BAM files. Then, the results of each chromosome group were subjected to HapHiC pipeline with nchr = 4 or 6 (for fused quartets). Next, we assigned the clustered unitigs into four haploid sets according to the.agp file obtained from the above step. The unmapped and unclustered unitigs were reassigned into four haploid sets according to the Hi-C contact records. We mapped HiFi and ultra-long ONT into unitigs by using minimap2 with -ax map-hifi -N 1-secondary = no parameters, and extracted reads according to the relationship between unitigs and haploid sets. Next, we extracted the raw reads of the four haplotypes using SeqKit (v2.8.0)^57^ according to the alignment above. The extracted reads for each haploid set were independently reassembled using Hifiasm (0.19.9-r616) and rescaffolded using HapHiC with nchr = 20–25. The generated scaffolds were finally polished using Juicebox (v2.20)^58^.

For the trio binning strategy, parental reads were first subjected to yak (https://github.com/lh3/yak) to generate paternal and maternal specific *k*-mer libraries. De novo assembly was performed using Hifiasm (0.19.8-r603) by combination of parental *k*-mer to obtain paternal contigs and maternal contigs. Hi-C reads were also split into two datasets according to parental *k*-mers. Paternal and maternal scaffolds were constructed by using juicer (v1.6)^59^, 3D-DNA (v180419)^60^ and Juicebox workflow.

Parental reads were mapped into scaffolds to distinguish paternal genome and maternal genome. The two versions of the genome were compared with each other using MUMmer (4.0.0rc1)^61^ to validate the quality. BUSCO (5.7.0)^62^ was also used to validate the completeness of assembly with actinopterygii_odb10 as a database. Merqury (v1.3)^63^ was used to evaluate the quality value of assembly. We mapped Hi-C, DNA-seq and RNA-seq short reads into the genome using BWA and HISAT2 (v2.2.1)^64^ to detect multiple mapping. The tag ‘XA:Z’ was used to identify multiple mapping records from alignment.

### Transposable element and gene annotation

EarlGrey (v4.2.4)^65^ was used to de novo predict the transposable element library and analysed their dynamics, including kimura distance among transposable element families. RepeatMasker (v4.1.5; http://www.repeatmasker.org) was used to soft mask the repetitive sequence before protein-coding gene prediction. We annotated protein-coding genes by using several strategies, including de novo prediction, RNA-seq-based prediction and homology-based prediction. RNA-seq reads were mapped to the genome using HISAT2, and the resulting alignments were assembled into transcripts with StringTie (v2.2.3) using a reference-guided approach. The transcripts were further annotated for gene structure using TransDecoder (v5.7.1). The alignment of RNA-seq data and the protein sequence of published snow carp were provided to BRAKER3 (v3.0.8)^66^ to perform a comprehensive gene prediction. Full-length and non-redundant isoforms were obtained through IsoSeq3 software (https://github.com/PacificBiosciences/pbbioconda) based on ISO-seq data. The generated transcripts were mapped to the reference genome using pbmm2. Finally, the gene models from above software were combined using EvidenceModeler (v2.1.0)^67^ to obtain final gene structures. The generated protein sequences were uploaded to eggnog-mapper^68^ for functional annotation.

### Phylogenomic analysis based on whole-genome alignment

A whole-genome alignment was generated using Cactus (v2.8.2)^69^ with a haploid subset (25 unfused chromosomes from the paternal genome) as the reference. Each chromosome alignment was divided into 20 bins. Within each bin, multiple alignments were generated using non-overlapping 30-kb windows. Each alignment was used to infer a phylogenetic tree with FastTree (v2.1.11)^70^, and the resulting trees were midpoint rooted. For a rooted tree with four branches, there are 15 possible topological categories, including three types of 2–2 tree and 12 types of 1–3 tree. All trees are classified into four categories, including three 2–2 tree types and other tree type (Fig. 2e), and the proportion in each bin was counted. Heterozygosity was calculated from the whole-genome alignment generated by Cactus, excluding gap regions.

### Tetraploid genotype calling

Short reads were mapped into the haploid subset (25 unfused chromosomes from paternal) of the *S. younghusbandi* genome using BWA mem algorithm. The alignments were subject to GATK (v4.5.0.0) HaplotypeCaller^71^ to call single-nucleotide polymorphism with *-*ploidy = 4, followed by filtration with genotype quality (QD) < 2.0 | | mapping quality (MQ) < 40.0 | | FisherStrand (FS) > 60.0 | | StrandOddsRatio (SOR) > 3.0 || MQRankSum < −12.5 || ReadPosRankSum < −8.0 parameters. Only biallelic site were restored and counted, such as 0/0/0/1, 0/0/1/1 and 0/1/1/1. The number of 0/0/1/1 genotypes were counted as disomic genotypes (AAaa), and the number of 0/0/0/1 and 0/1/1/1 were counted as tetrasomic genotypes (AAAa and Aaaa, respectively). The disomic genotype ratio was calculated by dividing the disomic genotype by the sum of the disomic and tetrasomic genotypes. Published resequencing data of *S. younghusbandi*^32^ were used to calculate the alternative allele depth and disomic genotype ratio.

### Syntenic analysis and gene loss analysis

Genomic synteny between the four haplotypes of *S. younghusbandi* was performed using JCVI (v1.4.11)^72^, MCScanX^73^ and GENESPACE (v1.3.1)^74^. The 1:1:1:1 syntenic gene pair was extracted from MCScanX results. KaKs_Calculator2.0 (ref. ^75^) was used to estimate *Ks* values between syntenic gene pairs with the YN method. Similar methods were used to identify 1:4, 1:3 and 1:2 syntenic genes between O*. macrolepis* and *S. younghusbandi*. From 1:2 syntenic gene groups, the gene groups that retained two copies present in only *uf* or *f* copies were defined as rediploidization-related gene loss (ohnologue loss) events. On the basis of synteny analysis, we examined the configurations of *uf*:*f* = 2:0 (*f* copy loss) and *uf*:*f* = 0:2 (*uf* copy loss), and counted their respective occurrences across the five fused events.

### Rediploidization age estimation

To estimate the rediploidization age, whole-genome alignment was performed across the four haplotypes and the outgroup *O. macrolepis* using Cactus. Intronic sequences were extracted from the alignment and partitioned into 2-Mb windows with a 1-Mb sliding step. Divergence times were inferred using MCMCTree (part of the paml package 4.10.7)^76^ under an independent rates clock model (clock = 2). The divergence time between *O. macrolepis* and all *S. younghusbandi* haplotypes was calibrated to 30–34 Ma (ref. ^33^). The mean divergence times and 95% credibility intervals obtained from this analysis were used for visualization and to infer the timing of chromosome fusion. The sample frequency was set to 100, with a final sample size of 20,000.

### Centromeric repeats identification

We first used TRASH (v1.2)^77^ to predict tandem repeats in 90 chromosome sequences. The most enriched repeats predicted for each chromosome were identified as candidates for centromeric repeats. RepeatMasker was then used to annotate the chromosomal locations of these candidate repeats and to assess whether they exhibited a unimodal distribution across the majority of chromosomes. On the basis of one most enriched centromeric repeats, TRASH was re-run to further predict all centromeric monomers on the genome by adding -seqt parameter. All annotated monomers were classified into Cen254 and Cen263 according to their length. Finally, the three monomers with the highest repetition frequency were selected and subjected to multiple sequence alignment using MAFFT to identify conserved regions.

### Centromere FISH validation

The centromeric sequence was synthesized by BGI Genomics, and the target fragment was amplified by PCR using primers (cen-R TTCTTTCTAATGAAATG and cen-F CATTTATCATGTTTTTGAGC). The amplified product was purified using a Gel Extraction Kit (D2500-02, Omega) and labelled with a probe using a Nick Translation Kit (10976776001, Roche). Chromosome slides were prepared from *S. younghusbandi* cells cultured in our laboratory using standard methods. First, cells were harvested and treated with a hypotonic solution (0.075 M KCl) to induce cell division, followed by fixation in Carnoy’s solution (3:1 methanol:acetic acid). The fixed cells were then dropped onto clean microscope slides, and the slides were placed in an oven to dry, allowing the chromosomes to spread. The prepared chromosome slides were subjected to enzymatic digestion at 37 °C for 1.5 h. The slides were then sequentially treated with 2× saline sodium citrate (SSC) washing, 4% paraformaldehyde fixation, PBS washing and ethanol gradient dehydration. Deionized formamide (70%) was added to the slides, treated at 70 °C for 1 min and 20 s, then ethanol gradient dehydration and ddH_2_O washing were performed. The labelled probe was denatured at 95 °C for 7 min, added to the chromosome slides and hybridized at 37 °C for 6 h. The slides were incubated with digoxin antibody (11207750910, Roche) at 37 °C for 1 h following washing, and stained with DAPI (2680173, Invitrogen). Finally, the slides were imaged using laser confocal fluorescence microscopy (Olympus FV3000). The oligonucleotide probes for Oligo-FISH were designed using the Chorus2 (v2.1)^78^, and the experiments were conducted following the same protocol.

### Meiosis FISH experiment

Testis were collected from an adult male *S. younghusbandi*, minced and then subjected to hypotonic treatment and fixation following the above method. Chromosome spreads were prepared from the resulting testicular cell suspension. Telomere probes were generated using the primers telo_F (TTAGGG)_4_ and telo_R (CCCTAA)_4._ Telemere probes were labelled using DIG-11-dUTP and centromere probes were labelled using biotin-16-dUTP. The FISH experiment was performed following the same method. Final image acquisition was performed using multimodality structured illumination microscopy (Multi-SIM, NanoInsights), and the reconstructed images were analysed with ImageJ (v2.9.0) software.

### Identification of young ohnologues

To identify young ohnologues, we extracted all four-copy genes derived from fused chromosomal quartets. Multiple sequence alignment was performed using MAFFT, and gene trees were constructed with FastTree and midpoint rooted. Only genes exhibiting a clear rediploidized topology ((*uf*, *uf*), (*f*, *f*)) were classified as young ohnologues. To accurately quantify the relative expression of *uf* and *f* copies, we identified rediploidized sites, defined as positions fixed for different alleles between *uf* and *f* copies, based on coding sequence alignments. Following screening, 2,449 ohnologue pairs were retained, each containing at least one rediploidized site.

### RNA-seq of different tissues

Multiple individuals of *S. younghusbandi* (sex not determined) approximately 1 year of age, with weight approximately 4 g and body length of approximately 80 mm, were selected. Eleven tissues (brain, gill, muscle, liver, gut, kidney, heart, bone, skin, bladder and spleen) were dissected and pooled from several individuals. Total RNA was extracted from each pooled tissue sample. All RNA-seq library preparation and sequencing were performed in OneMore. All experimental procedures were approved by the IACUC of Southwest University (IACUC-20260126-05).

### Relative expression levels between *uf* and *f* copies

For RNA-seq data from 11 tissues, reads were aligned to the haploid genome (all unfused p1 copy, including *uf* copy from paternal) using HISAT2. SNP calling was conducted following GATK best practices for RNA-seq data including steps such as Picard tools, SplitNCigarReads and HaplotypeCaller. Heterozygous sites detected in the RNA-seq data were used to assign transcripts to either the *uf* or *f* copy. Genes containing at least three such informative sites from RNA-seq data were retained for subsequent analysis. The relative expression levels of *uf* and *f* copies were determined based on read depths at *uf*-specific and *f*-specific sites to the total depth. A gene was considered *uf* biased if the *uf* versus total expression ratio exceeded 0.65, and *f* biased if the ratio was below 0.35. On the basis of read counts from featureCounts (v2.0.6)^79^, gene expression levels was normalized to transcripts per million (TPM). The expression levels of the *uf* and *f* ohnologue were derived by multiplying the TPM values by their corresponding read depth ratios, respectively.

### RNA-seq for cold exposure

One-year-old *S. younghusbandi* (sex not determined) reared at 22 °C were divided into two groups: a control group and a cold-induced group. The cold-induced group was gradually cooled at a rate of 1.5 °C per year, maintained at this temperature for 24 h, whereas the control group was kept at 22 °C. Six individuals per group were sampled for biological replication, and RNA-seq was performed on muscle, liver, brain, heart and gill tissues. Reads were mapped into a haploid reference genome (comprising 25 unfused chromosomes, which included all p1 haplotype and *uf* copy of paternal origin) and perform reads count by using featureCounts. Differentially expressed genes were identified using the DESeq2 (v1.44) package. Genes showing significant differential expression were defined by |log_2_(fold change)| > 1 and adjusted *P* < 0.01. For the differentially expressed ohnologue pairs, we calculated the TPM of *uf* and *f* (mean value across all biological replicates). We then computed the ratios of *uf* (5 °C) versus* uf* (22 °C) and *f* (5 °C) versus *f* (22 °C) to examine whether both *uf* and *f* responded to cold in a proportional manner.

### Hi-C analysis

Published Hi-C data of *A. laticeps*^80^*, Schizothorax o’connori*^8^*, Oxygymnocypris*^81^ *and Gymnocypris przewalskii*^82^ were mapped into their corresponding contigs by using Chromap (0.2.6-r490)^83^. We mapped these contigs into the haploid subset of the *S. younghusbandi* genome using minimap2. A Hi-C contact heatmap between 25 chromosomes was generated from pair results of Chromap and then normalized by the KR^84^ method.

### Genome assembly of *Schizothorax curvilabiatus*

A male specimen of *S. curvilabiatus* was collected in Mêdog county, Tibet, China. Genomic DNA was extracted from muscle for the construction of PacBio HiFi and Hi-C sequencing libraries, following protocols previously described for *S. younghusbandi*. All experimental procedures were approved by the IACUC of Southwest University (IACUC-20260126-02). Approximately 108 Gb of PacBio HiFi data and 156 Gb of Hi-C data were generated. A haplotype-resolved genome assembly was performed using the same approach as for *S. younghusbandi* without ONT reads, resulting in a final assembly size of 3.73 Gb. The four haplotypes were arbitrarily labelled as h1–h4, and chromosomes involved in fusion events were labelled as *uf1/uf2* and *f1*/*f2*. A chromosomal collapse was identified in the *f2* copy of chromosomes 19–22 and was rescued by manual correction of the inserted segments from chromosomes 19–22 (*f1*) into chromosomes 19–22 (*f2*). Protein-coding genes were annotated using BRAKER3 (v3.0.8)^66^ with RNA-seq data from a closely related species (*S. o’connori*)^85^. The phylogenomic and syntenic analysis was performed using the same approach as for *S. younghusbandi*.

### Ancestral rediploidization test

We used two phylogenetic approaches based on whole-genome alignments and concatenated orthologous protein sequences to test ancestral and independent rediploidization models. Whole-genome alignments were generated using Cactus for four haplotypes of *S. younghusbandi*, four haplotypes of *S. curvilabiatus* and the outgroup species *O. macrolepis*. Phylogenetic trees were constructed in 30-kb sliding windows, and the approximately unbiased test^86^ was applied to assess whether the topologies rejected either ancestral or independent rediploidization scenarios (*P* < 0.05). The proportions of windows supporting each of the two models were then summarized in 2-Mb intervals. Using MMseqs2 (17-b804f)^87^, we identified 684 and 486 orthologous groups in chromosome 19 and chromosome 22, respectively. Protein sequences were aligned with MAFFT, and all alignments were concatenated within each 2-Mb window. Phylogenetic trees were constructed with IQ-TREE and subjected to the approximately unbiased test (-n 0 -zb 10000 -zw -au).

### Reporting summary

Further information on research design is available in the Nature Portfolio Reporting Summary linked to this article.

## Online content

Any methods, additional references, Nature Portfolio reporting summaries, source data, extended data, supplementary information, acknowledgements, peer review information; details of author contributions and competing interests; and statements of data and code availability are available at 10.1038/s41586-026-10439-1.

## Supplementary information

Supplementary Figures Supplementary Figs. 1–16.

Reporting Summary

Supplementary Tables Supplementary Tables 1–15.

Peer Review File

## Data Availability

The accession numbers for the raw data used for genomic survey are provided in Supplementary Tables 1 and 15. The raw sequencing data (PacBio HiFi, ONT, Hi-C and short reads) for *S. younghusbandi* and *S. curvilabiatus* were deposited in the Sequence Read Archive database under the corresponding accession numbers PRJNA1210471 and PRJNA1394917. The raw data of RNA-seq generated in this study can be found in the NCBI under the accession number PRJNA1402086. The four assembled haplotypes of the *S. curvilabiatus* genome can be accessed via the European Nucleotide Archive under the accession numbers GCA_978021885.1, GCA_978021895.1, GCA_978021905.1 and GCA_978021915.1. The assembled *S. younghusbandi* genome can be found in Figshare^88^ (10.6084/m9.figshare.31700350). The annotation files can be found in Figshare^89,90^ (10.6084/m9.figshare.28468799 and 10.6084/m9.figshare.31101751). Raw imaging data of chromosome spreading can be found in Figshare^91^ (10.6084/m9.figshare.31143715). The BUSCO database used in this study is available (https://busco-data.ezlab.org/v5/data/lineages).

## Code availability

The codes and pipelines used in this study can be found on GitHub (https://github.com/csxie-swu/Sy-genome).
