# Supplementary material for: Chromosomal fusions trigger rediploidization of autopolyploid genomes
Source: Nature. 2026 Apr 22;654(8119):706–13. doi: 10.1038/s41586-026-10439-1 (PMC13275295; doi:10.1038/s41586-026-10439-1)
Supplement: Supplementary file 1 — Supplementary Figs. 1–16. [file 41586_2026_10439_MOESM1_ESM.pdf]

nature portfolio

https://doi.org/10.1038/s41586-026-10439-1

Supplementary information

Chromosomal fusions trigger rediploidization of autopolyploid genomes

In the format provided by the authors and unedited

Supplementary information

for “Chromosomal fusions trigger rediploidization of autopolyploid genomes”

Chuanshuai Xie[1,#], Zitu Ma[1,#], Chaowei Zhou[1,#], Kexin Ma[1,#], Haoyu Wang[1], Jiahong Wu[1], Yan Zhou[1], Yongrui Lu[1], Da Ji[1], Xuedie Gu[2], He Gao[1], Junting Li[1], Suxing Fu[1], Weiqiang Li[1], Zhaofang Han[1], Shijun Xiao[3], Fei Liu[4], Benhe Zeng[4], Shen’ao Chen[5], Jiangong Niu[6], Tao Zhang[6], Jian Shen[6], Chunna Liu[7], Jing Luo[2], Daniel J. Macqueen[8], Axel Meyer[9,10,11,*], Haiping Liu[1,*], Luohao Xu[1,*]

*Correspondence:

luohaox@swu.edu.cn, luihappying@163.com, axel.meyer@uni-konstanz.de

# equal contribution

Contents

Supplementary Figure 1 Morphological characteristics and altitude distribution of three group of snow carps.
Supplementary Figure 2 Kmer-distribution of hexaploid snow carp (*Schizothorax wangchiachii*).
Supplementary Figure 3 *S. younghusbandi* trio construction and assembly.
Supplementary Figure 4 Comparison of the read-binning assembly and the trio-based assembly.
Supplementary Figure 5 Landscape of transposable element (TEs) for *S. younghusbandi*.
Supplementary Figure 6 Selection pressure analysis of fused and non-fused quartets.
Supplementary Figure 7 The distribution of alternative allele depth for tetrasomic and disomic quartets.
Supplementary Figure 8 Distribution of bivalent and multivalent inheritance genotypes across chromosomes.
Supplementary Figure 9 Divergence time estimates for wave2 based on an independent rate clock model.
Supplementary Figure 10 The karyotype of meiotic metaphase I cells in the testis.
Supplementary Figure 11 Features of *S. younghusbandi* centromere.
Supplementary Figure 12 Average sequence divergence (*Ks*) among all pairwise homologous in short and long arm.
Supplementary Figure 13 Gene loss in *S. younghusbandi*.
Supplementary Figure 14 Expression patterns of *uf* and *f* ohnologs in eleven tissues.
Supplementary Figure 15 Gene expression level between ohnolog *uf* and *f.*
Supplementary Figure 16 Phylogenetic tree topologies on chromosome 19 and 22.

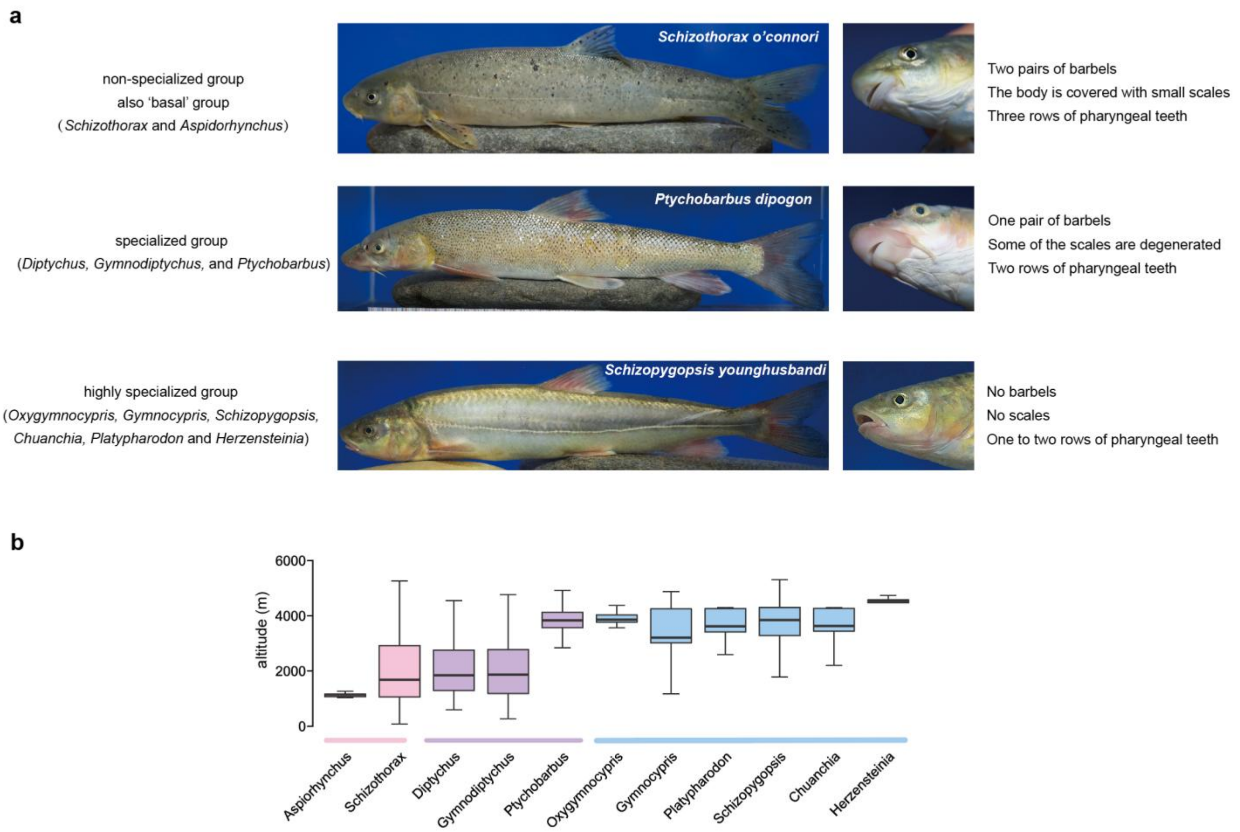

Supplementary Figure 1 Morphological characteristics and altitude distribution of three group of snow carps. (a) Morphological features of three species of schizothoracine fish: the left side shows the whole body, and the right side shows a close-up of the head (photo credit, He Gao). (b) Altitude distribution of 11 genera of snow carps. The color represent different morphological groups.

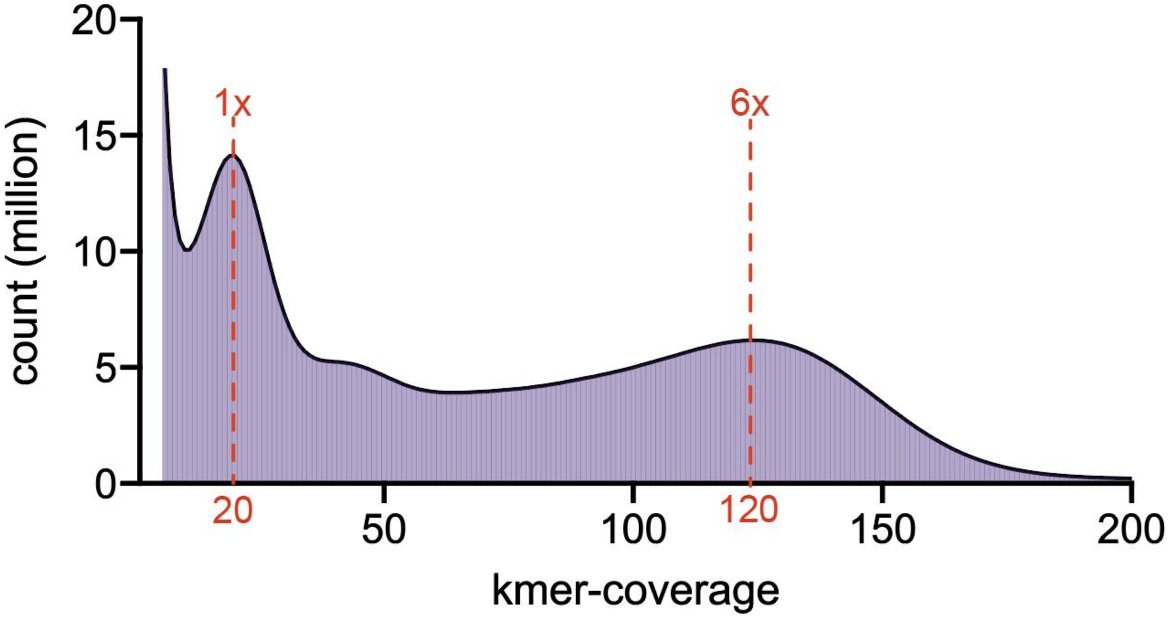

Supplementary Figure 2 Kmer-distribution of hexaploid snow carp (*Schizothorax wangchiachii*).

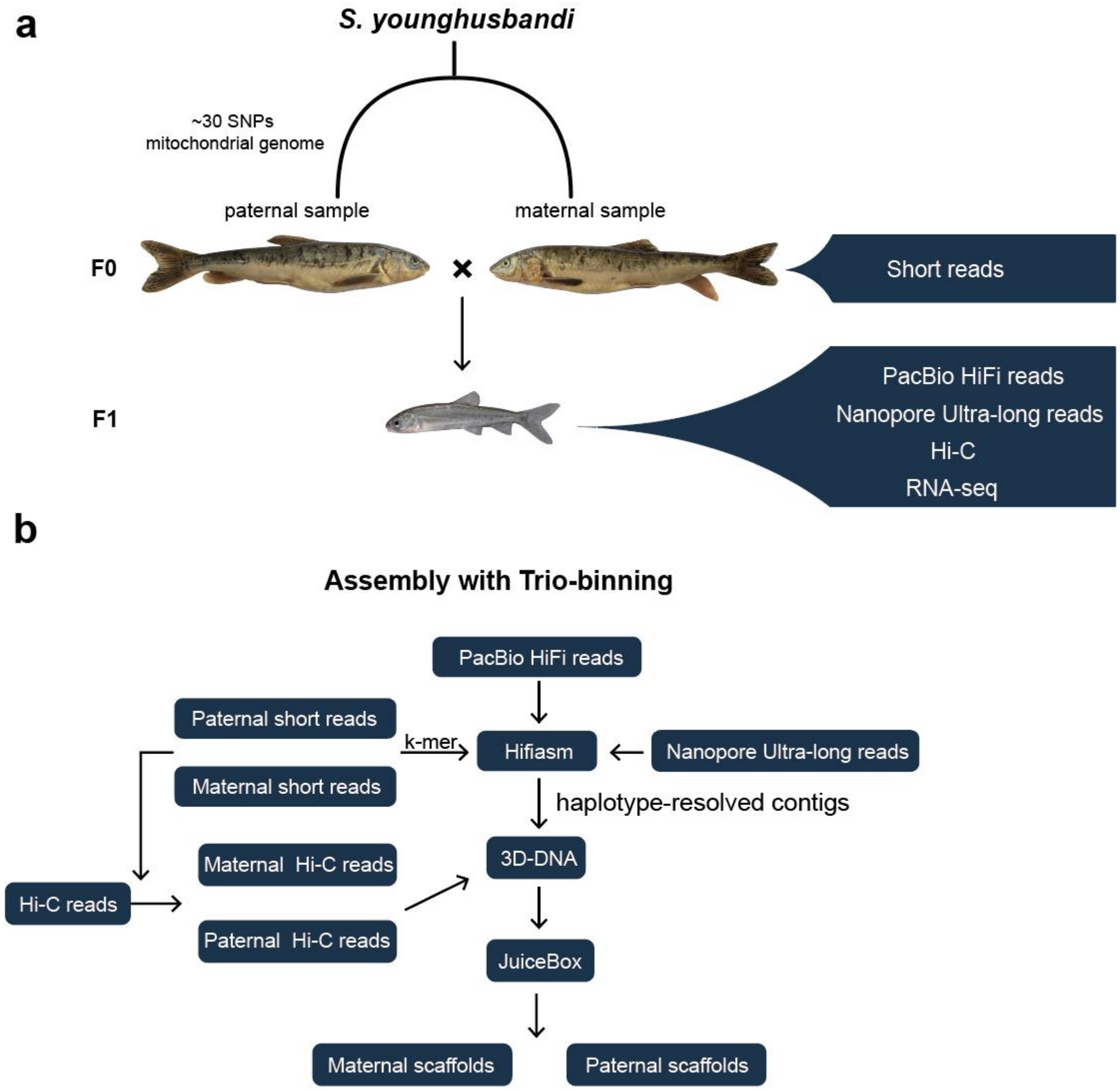

Supplementary Figure 3 *S. younghusbandi* trio construction and assembly. (a) The trio of *S. younghusbandi* consists of two parents with mitochondrial genomes differing by 30 SNPs and their offspring (photo credit, He Gao).(b) Workflow of assembly with trio-binning.

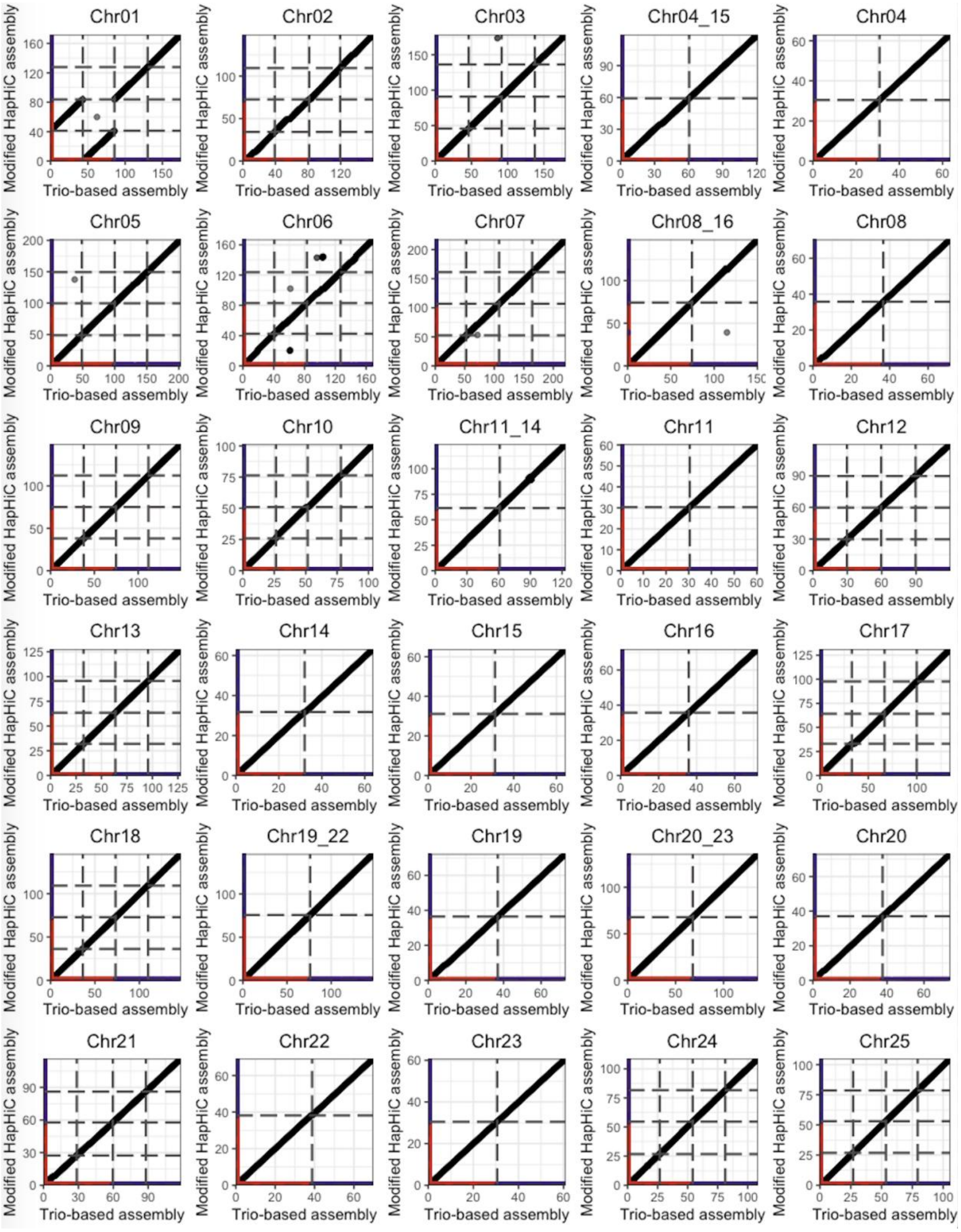

Supplementary Figure 4 Comparison of the read-binning assembly and the trio-based assembly. The dot plot shows the collinearity of the chromosomes from two assembly version. The x-axis is tiro-based assembly, and the y-axis is read-binning assembly. Red and purple represent haplotypes from maternal and paternal, respectively.

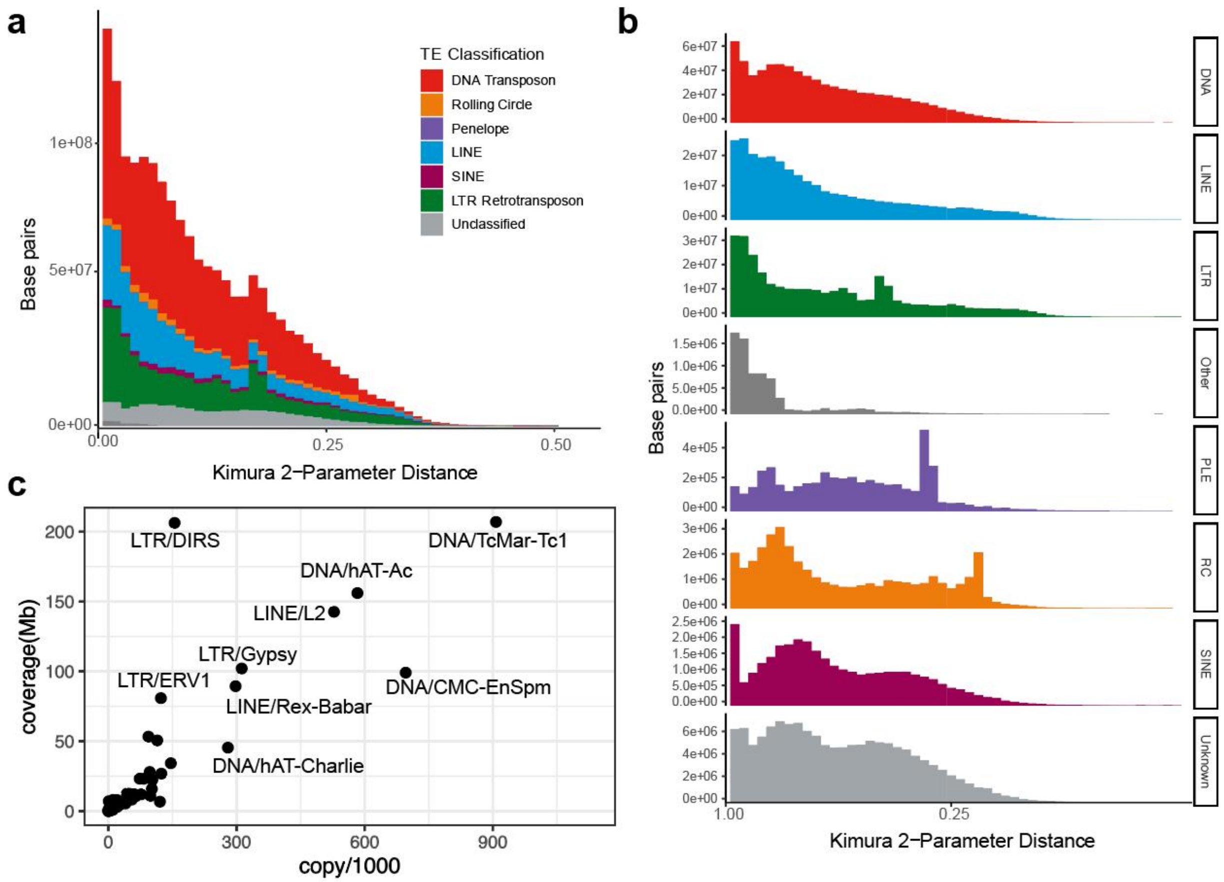

Supplementary Figure 5 Landscape of transposable element (TEs) for *S. younghusbandi*. (a) and (b) Distribution of kimura distance of each type of TEs. Different colors represent different TE classe. (c) Scatter plot of copy number (x-axis) versus total length in base pairs (y-axis) for individual TE families. Abundant families are highlighted.

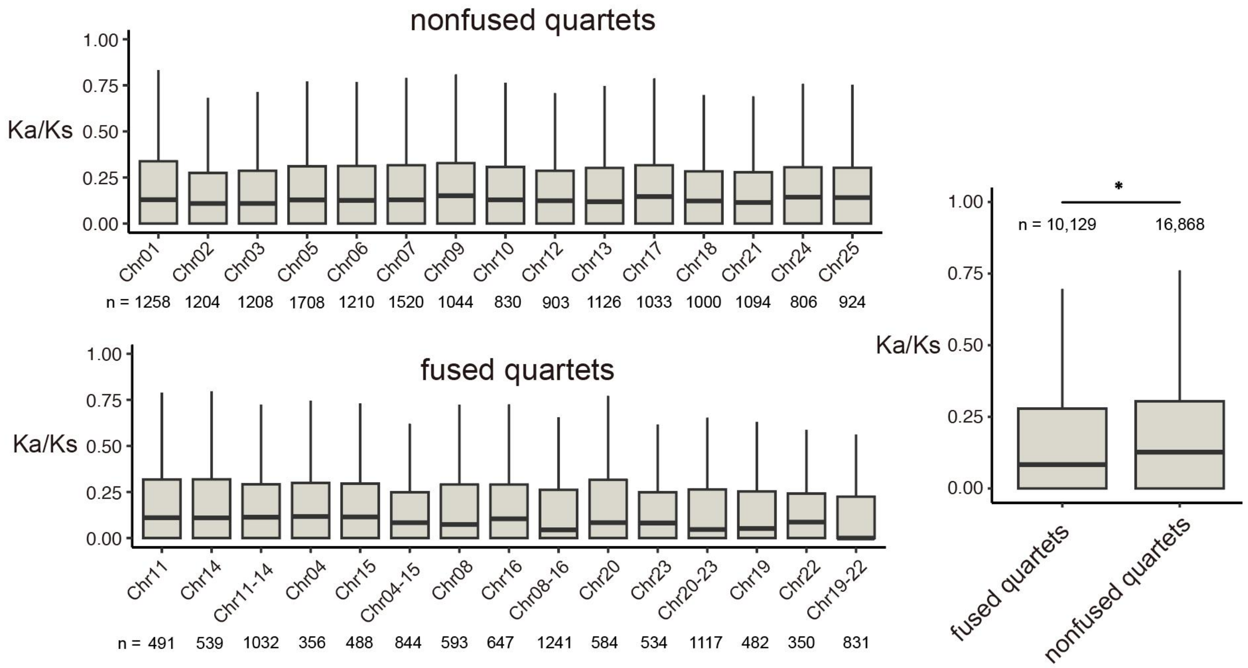

Supplementary Figure 6 Selection pressure analysis of fused and non-fused quartets. The left panel shows the *Ka/Ks* ratio for each chromosome, while the right panel presents the aggregated distribution. Asterisks denote statistical significance (two-sided Mann-Whitney U test, $p < 0.01$). The chromosome name Chr11-14, Chr04-15, Chr08-16, Chr20-23 and Chr19-22 indicate the fused chromosomes (*f* copy). Each dot represents one gene pair (n indicated above/below). The box plots display the median (center line), the interquartile range (box), and the data range within 1.5×IQR (whiskers). The sample size (n) denotes the number of homologous gene pairs used in the calculation, as labeled below.

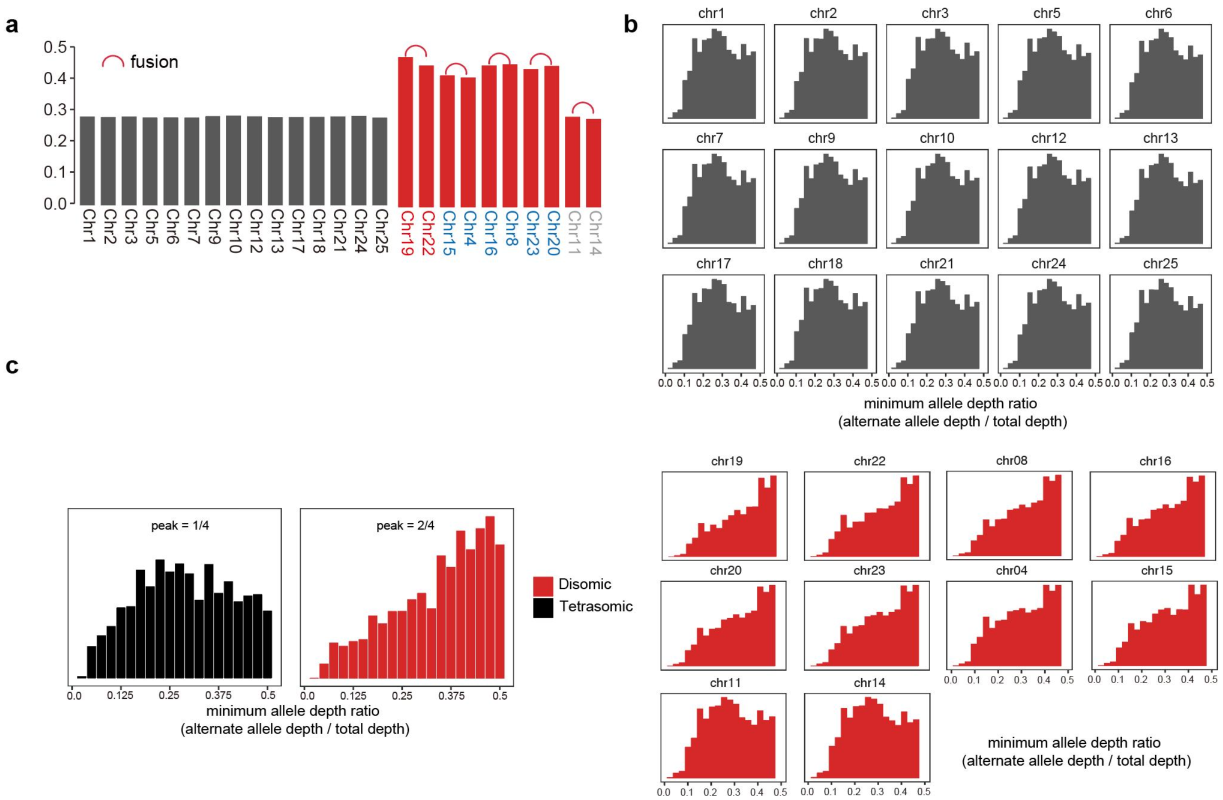

Supplementary Figure 7 The distribution of alternative allele depth for tetrasomic and disomic quartets. (a) Ratio of disomic genotype (AAaa) among all heterozygous tetraploid genotype. The red line represents the 5 pairs of fused chromosomes. The three waves of rediploidization are indicated by the colors of the chromosome names. Two-sided Mann-Whitney U test was used to compare the differences between fused and unfused quartets. (b) Alternative allele depth distritbution for non-fused quartets (top) and fused quartets (bottom). (c) The distinct distribution pattern for tetrasomic and disomic quartets shows that the peak of the depth ratio is close to 0.25 under tetrasomic inheritance, whereas it is close to 0.5 under disomic inheritance. Among them, Chr11 and Chr14, despite being fused quartets, have not yet exhibited a disomic inheritance pattern, as they may have undergone recent fusion. All tetrasomic chromosomes are highlighted in red.

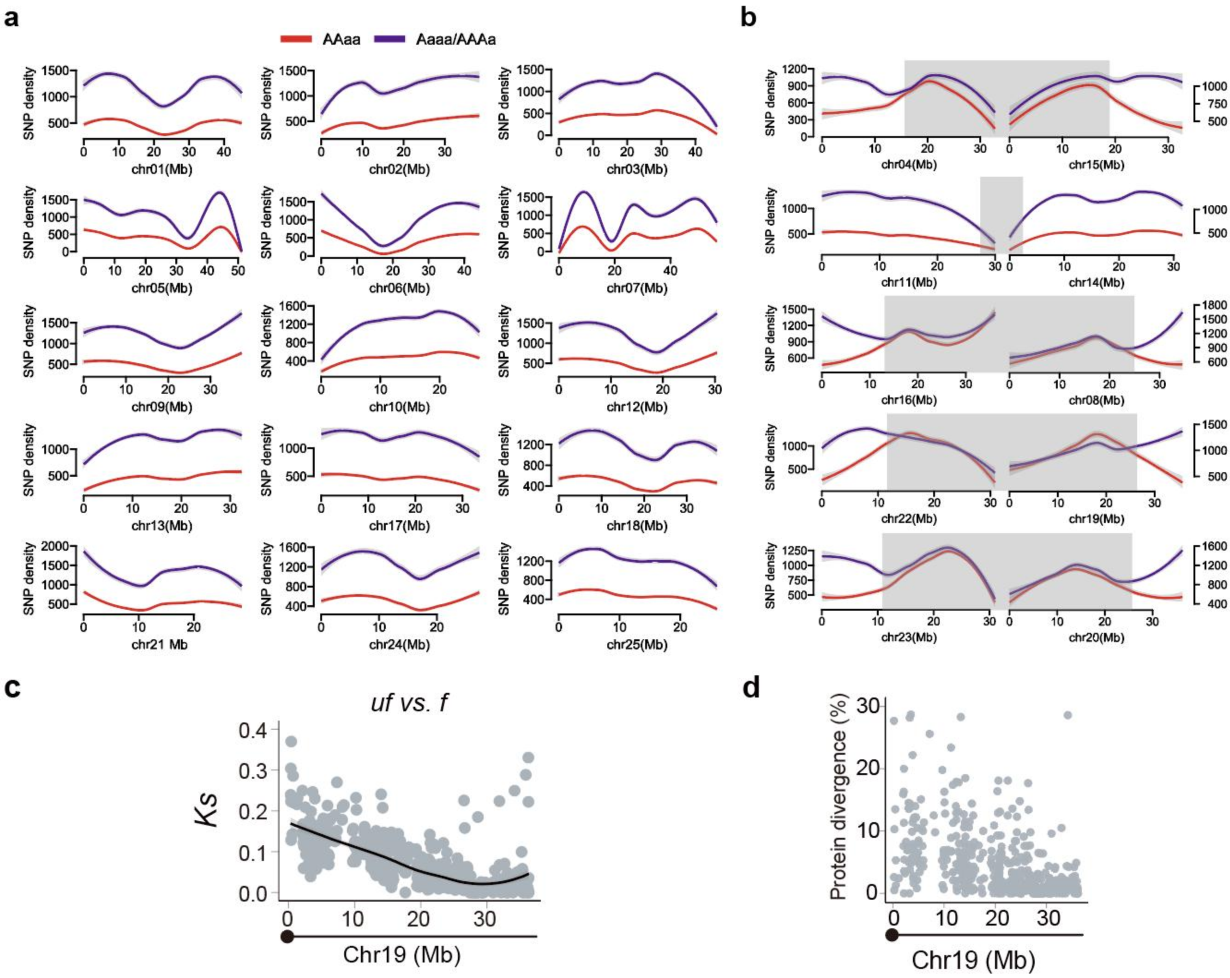

Supplementary Figure 8 Distribution of bivalent and multivalent inheritance genotypes across chromosomes. (a) non-fused quartets (b) fused quartets. The tetrasomic and disomic genotypes are labeled in blue and red respectively. (c) The distribution of *Ks* values between homologs (unfused *vs.* fused) along the chromosome is elevated at the fusion site. (d) The distribution of protein sequence divergence is elevated at the fusion site. The black dots indicate the locations of fusion sites.

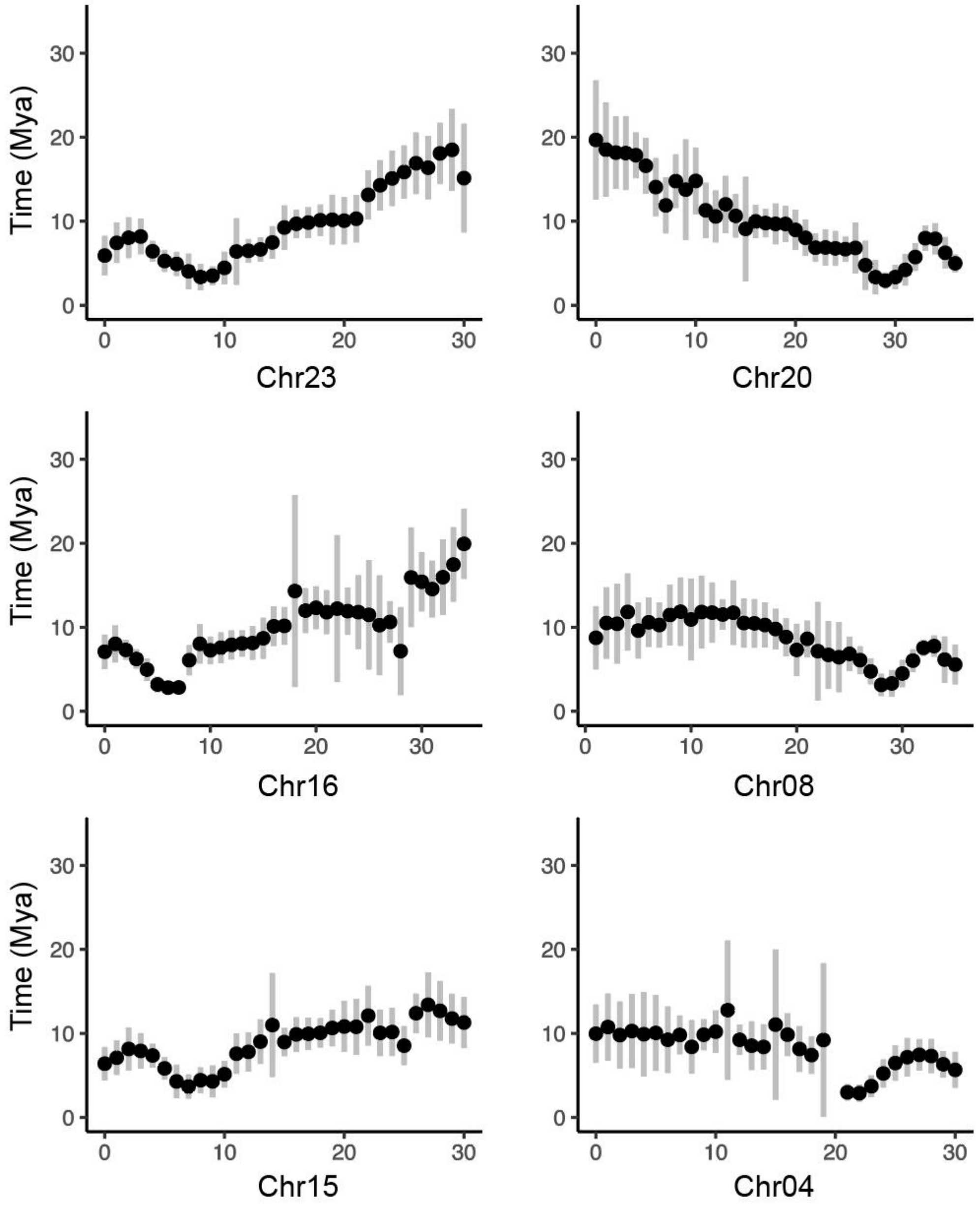

Supplementary Figure 9 Divergence time estimates for wave2 based on an independent rate clock model. For each genomic window, the point shows the median estimate of node age, and the bar indicates the 95% HPD interval.

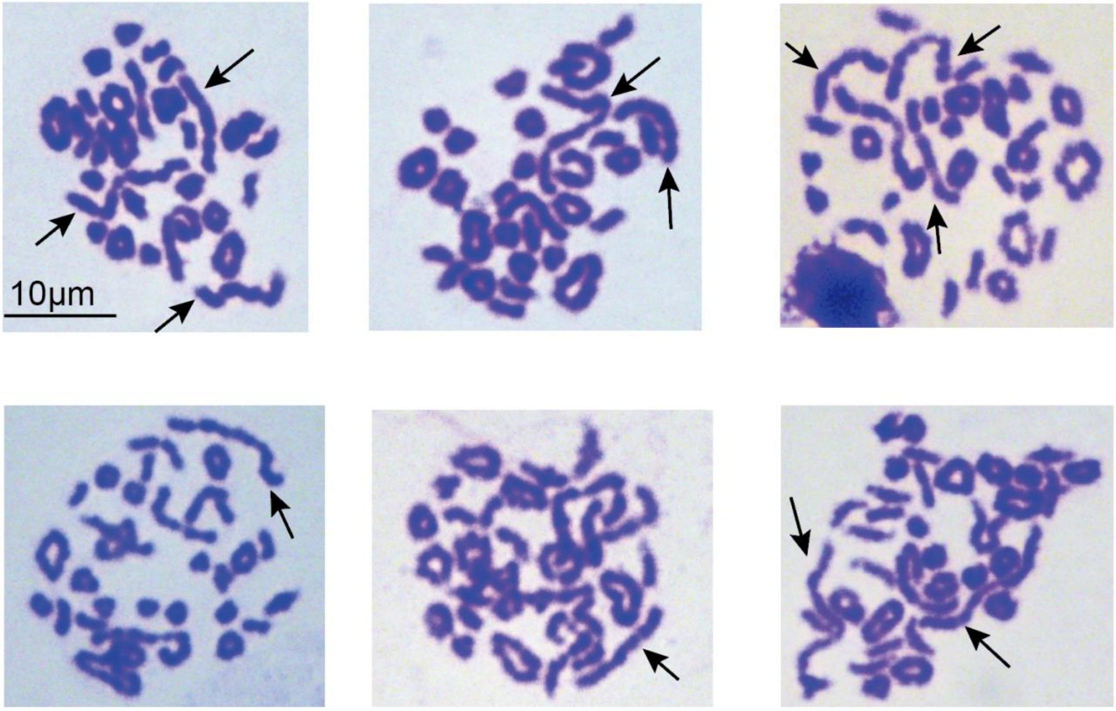

Supplementary Figure 10 The karyotype of meiotic metaphase I cells in the testis. The arrows point to the chain-shaped pairing structures. Multiple centromere constrictions can be observed in those long chain pairing structures.

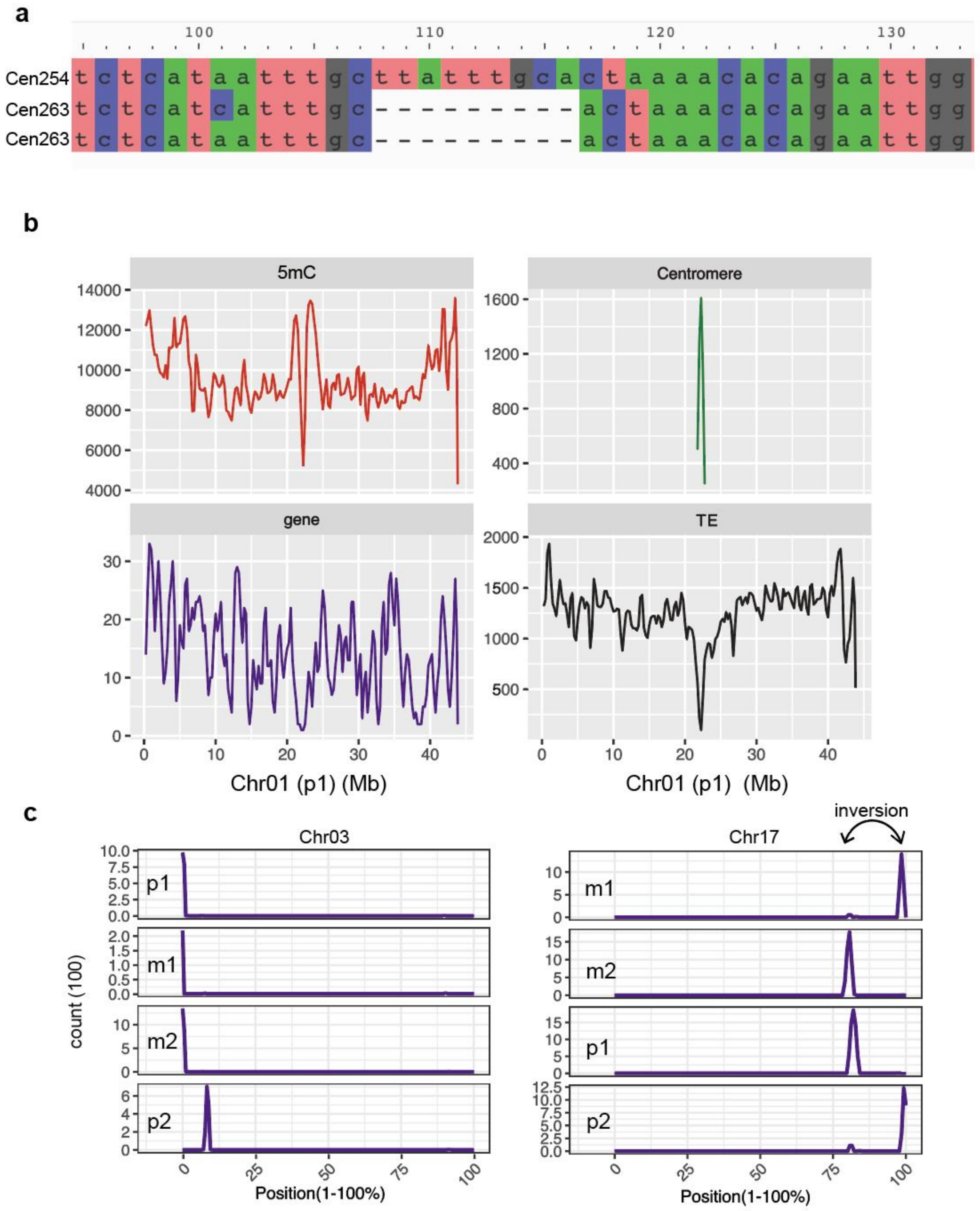

Supplementary Figure 11 Features of *S. younghusbandi* centromere. (a) Sequence differences between Cen254 and Cen263. Different colored backgrounds represent different bases (visualized using AliView). (b) Distribution of DNA methylation (red line), centromeric repeats (green line), gene density (purple line), and transposable element density (black line) on Chr01 (p1). (c) Centromeric inversion on Chr03 and Chr17.

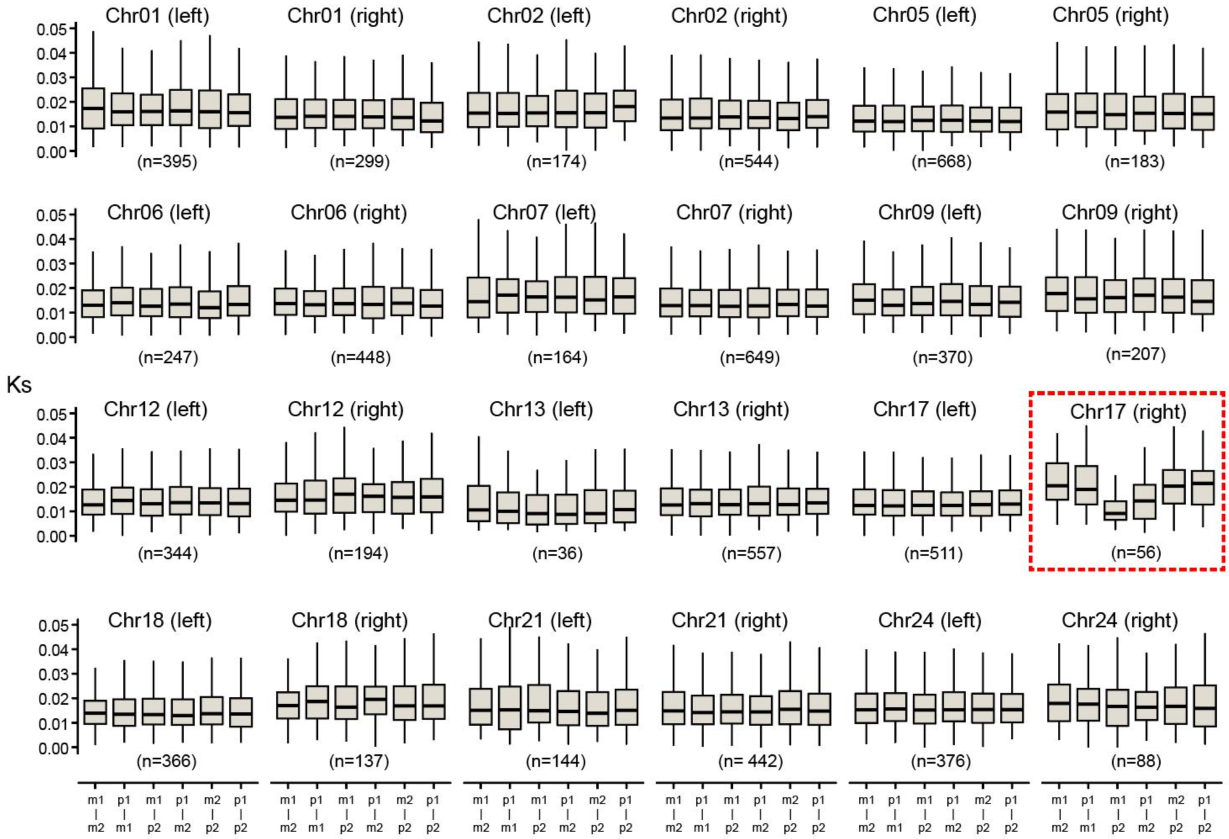

Supplementary Figure 12 Average sequence divergence (*Ks*) among all pairwise homologous in short and long arm. In the unfused quartets, except for Chr17 (marked in red), the long and short arms exhibited consistent patterns. "Left" and "right" denote the left- and right-flanking regions of the centromere, corresponding to the long or short arm. Centromere positions are shown in Extended Data Fig. 3d. The three telocentric chromosomes (Chr03, Chr10, and Chr25) were excluded from the analysis. The box plots display the median (center line), the interquartile range (box), and the data range within 1.5×IQR (whiskers). The sample size (n) denotes the number of homologous gene pairs used in the calculation, as labeled below.

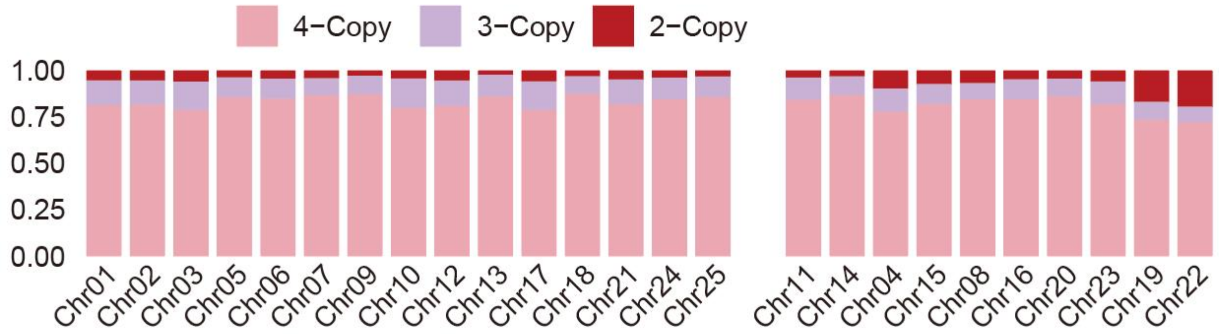

Supplementary Figure 13 Gene loss in *S. younghusbandi*. The proportion of 2-copy, 3-copy, and 4-copy genes between tetrasomic chromosomes and disomic chromosomes. The left chromosomes is tetrasomic and the right chromosomes is disomic.

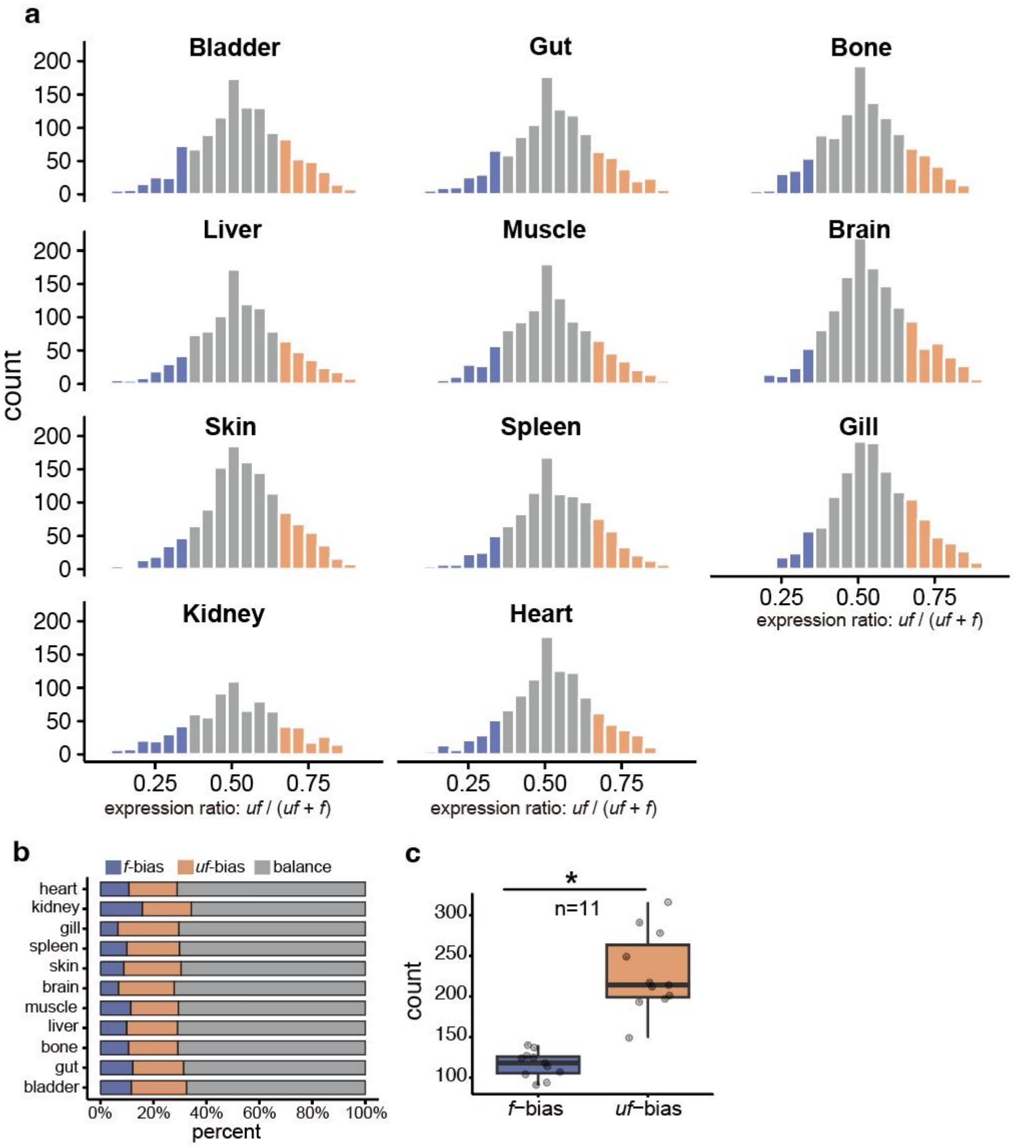

Supplementary Figure 14 Expression patterns of *uf* and *f* ohnologs in eleven tissues. (a) Distribution of the expression ratios of *uf* ohnolog. Ohnologs with the *uf* copy contributing more than 0.65 of the total expression are defined as *uf*-bias (marked in orange), those with ratio lower than 0.35 are defined as *f*-bias (marked in blue); and the remaining ohnologs are classified as balanced (marked in grey). (b) Proportion of three ohnolog expression patterns across eleven tissues. (c) Boxplot shows that the proportion of the *uf*-biased pattern is significantly higher than that of the *f*-biased pattern (two-tailed paired t-test, p-value < 0.05). Plots display the median (center line), interquartile range (box), and the data range within 1.5×IQR (whiskers). The sample size (n) denotes the number of pairwise comparisons across 11 tissues.

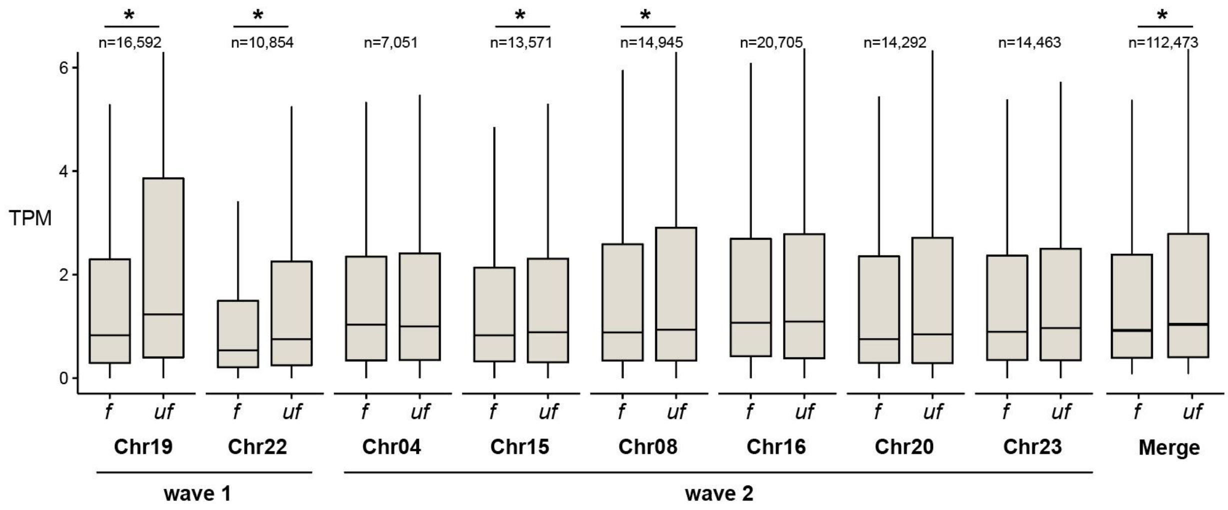

Supplementary Figure 15 Gene expression level between ohnolog *uf* and *f.* RNA-seq data across eleven tissues were combined and subjected to a two-tailed t-test, with asterisks indicating statistical significance at p-value < 0.05. The box plots display the median (center line), the interquartile range (box), and the data range within 1.5×IQR (whisker).The sample size (n) denotes the number of homologous gene pairs used in the calculation, as labeled above.

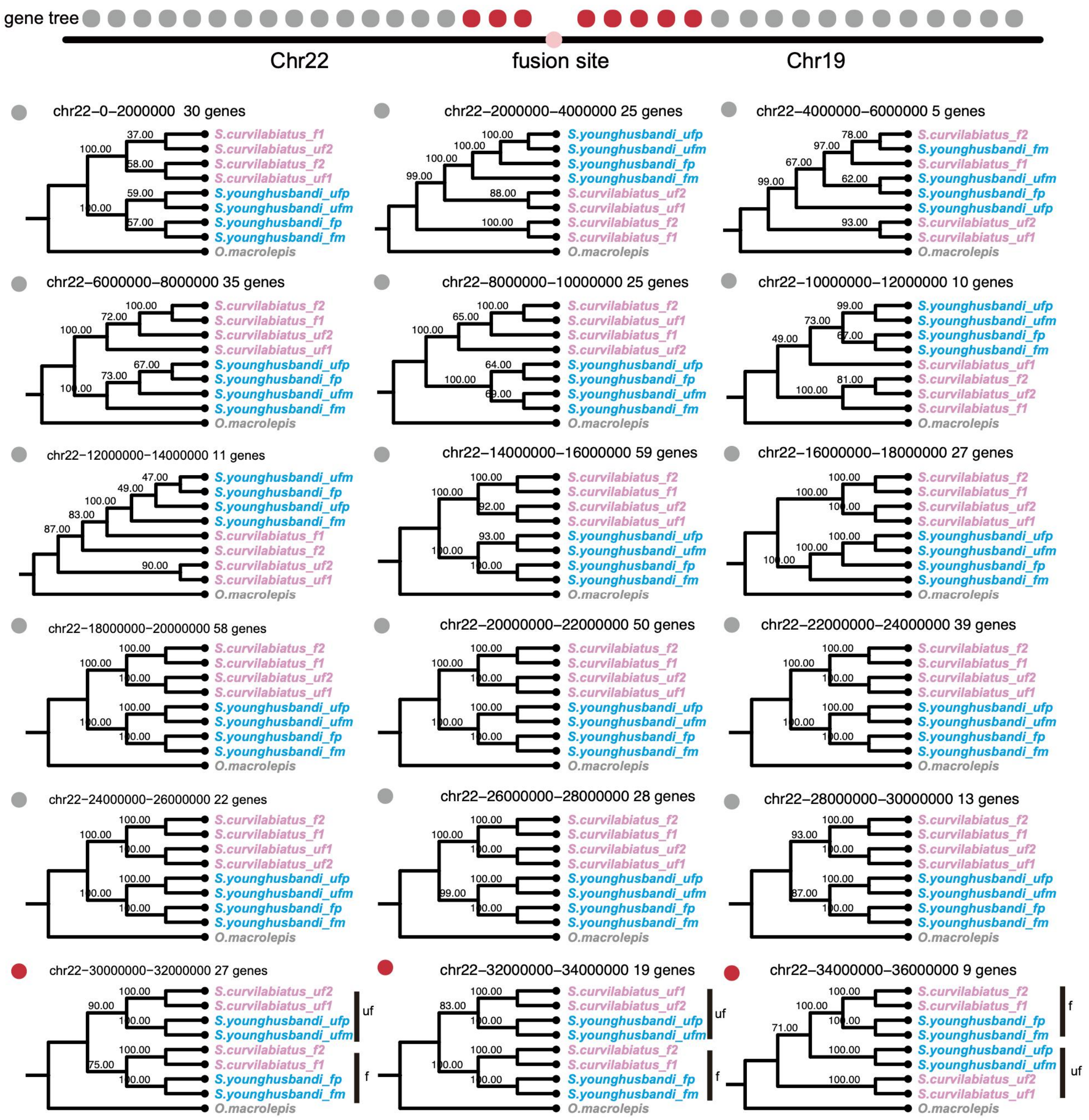

gene tree
Chr22
fusion site
Chr19
chr22-0-2000000 30 genes
chr22-2000000-4000000 25 genes
chr22-4000000-6000000 5 genes
chr22-6000000-8000000 35 genes
chr22-8000000-10000000 25 genes
chr22-10000000-12000000 10 genes
chr22-12000000-14000000 11 genes
chr22-14000000-16000000 59 genes
chr22-16000000-18000000 27 genes
chr22-18000000-20000000 58 genes
chr22-20000000-22000000 50 genes
chr22-22000000-24000000 39 genes
chr22-24000000-26000000 22 genes
chr22-26000000-28000000 28 genes
chr22-28000000-30000000 13 genes
chr22-30000000-32000000 27 genes
chr22-32000000-34000000 19 genes
chr22-34000000-36000000 9 genes
S.curvilabiatus_f1
S.curvilabiatus_f2
S.curvilabiatus_uf1
S.curvilabiatus_uf2
S.younghusbandi_ufp
S.younghusbandi_ufm
S.younghusbandi_fp
S.younghusbandi_fm
O.macrolepis
uf
f

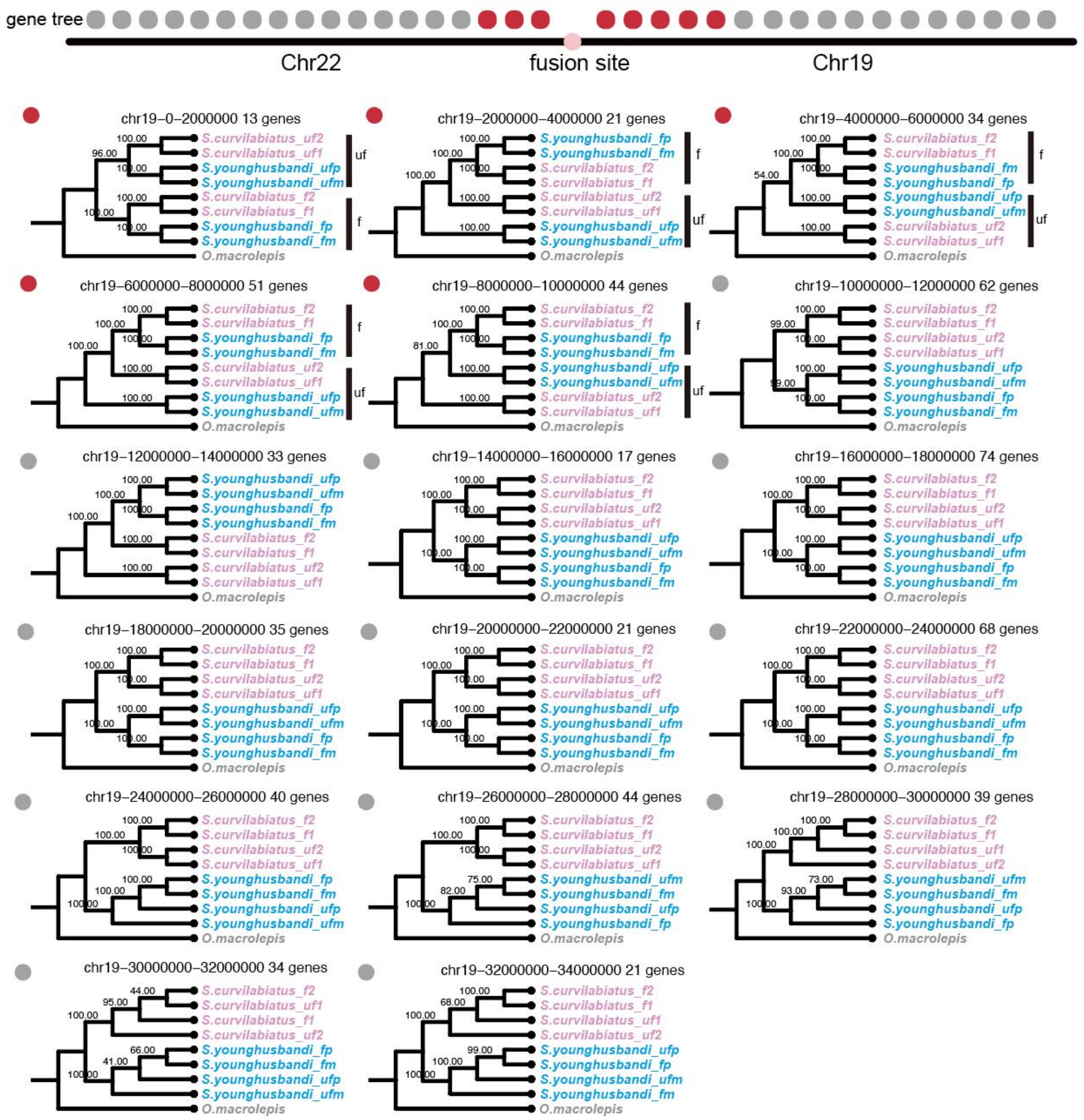

Supplementary Figure 16 Phylogenetic tree topologies on chromosome 19 and 22. Maximum-likelihood phylogenetic trees were constructed for genes within each 2-Mb window using concatenated alignments (protein coding genes). The approximately unbiased (AU) test was applied to compare two alternative models: ancestral rediploidization and independent rediploidization. Tree topologies supporting ancestral rediploidization are highlighted in red, whereas those supporting independent rediploidization are shown in gray. Bootstrap support values are indicated on each tree, and branch colors denote different species. At the top, the positions of individual windows for Chr19 and Chr22 are shown, with points colored according to the model they support. The approximate locations of fusion sites are indicated by pink dots.
