# Supplementary material for: Chromosomal fusions trigger rediploidization of autopolyploid genomes
Source: Nature. 2026 Apr 22;654(8119):706–13. doi: 10.1038/s41586-026-10439-1 (PMC13275295; doi:10.1038/s41586-026-10439-1)
Supplement: Supplementary file 2 — Reporting Summary [file 41586_2026_10439_MOESM2_ESM.pdf]

nature portfolio

Corresponding author(s): Axel Meyer, Haiping Liu, Luohao Xu

Last updated by author(s): Mar 11, 2026

Reporting Summary

Nature Portfolio wishes to improve the reproducibility of the work that we publish. This form provides structure for consistency and transparency in reporting. For further information on Nature Portfolio policies, see our Editorial Policies and the Editorial Policy Checklist.

Statistics

For all statistical analyses, confirm that the following items are present in the figure legend, table legend, main text, or Methods section.

n/a	Confirmed	
☐	☒	The exact sample size (*n*) for each experimental group/condition, given as a discrete number and unit of measurement
☐	☒	A statement on whether measurements were taken from distinct samples or whether the same sample was measured repeatedly
☐	☒	The statistical test(s) used AND whether they are one- or two-sided<br>*Only common tests should be described solely by name; describe more complex techniques in the Methods section.*
☒	☐	A description of all covariates tested
☐	☒	A description of any assumptions or corrections, such as tests of normality and adjustment for multiple comparisons
☐	☒	A full description of the statistical parameters including central tendency (e.g. means) or other basic estimates (e.g. regression coefficient) AND variation (e.g. standard deviation) or associated estimates of uncertainty (e.g. confidence intervals)
☐	☒	For null hypothesis testing, the test statistic (e.g. *F*, *t*, *r*) with confidence intervals, effect sizes, degrees of freedom and *P* value noted<br>*Give P values as exact values whenever suitable.*
☐	☒	For Bayesian analysis, information on the choice of priors and Markov chain Monte Carlo settings
☒	☐	For hierarchical and complex designs, identification of the appropriate level for tests and full reporting of outcomes
☒	☐	Estimates of effect sizes (e.g. Cohen's *d*, Pearson's *r*), indicating how they were calculated

Our web collection on statistics for biologists contains articles on many of the points above.

Software and code

Policy information about availability of computer code

Data collection: No software used for data collection.

Data analysis:
Genome survey: genomescope 2.0, KMC(3.1.1)
Assembly: MitoZ (v3.4), Hifiasm (0.19.8-r603),Hifiasm (0.19.9-r616), HapHiC (v1.0.2), yak(r56), 3D-DNA (v180419), juicer (1.6), Juicebox (v2.20), Seqkit (v2.8.0), Merqury (v1.3), BUSCO (5.7.0),MUMmer (4.0.0rc1)
Annotation : EarlGrey (4.2.4) , HISAT2 (2.2.1), RepeatMasker (4.1.5), StringTie (v2.2.3) , TransDecoder (v5.7.1), BRAKER3 (3.0.8), EvidenceModeler (v2.1.0), IsoSeq3
Phylogenetics: Cactus (v2.8.2), FastTree (2.1.11), IQ-TREE (v2.0.3), MAFFT (v7.526)
SNPcalling: GATK (4.5.0.0) ,BWA (0.7.18)
Rediplodization analysis: JCVI (1.4.11), MCScanX(v1.0), KaKs_Calculator2.0, paml (4.10.7), MMseqs2 (17-b804f), GENESPACE (1.3.1)
Centromere analysis: TRASH (v1.2), RepeatMasker (4.1.5)
FISH image: Chorus2 (v2.1) , Image J (2.9.0)
RNA-seq analysis: HISAT2 (2.2.1), featureCounts (v2.0.6), DESeq2 package (v1.44)
Hi-C analysis: Chromap (0.2.6-r490), minimap2(2.28)
Custom code available at https://github.com/csxie-swu/Sy-genome.

For manuscripts utilizing custom algorithms or software that are central to the research but not yet described in published literature, software must be made available to editors and reviewers. We strongly encourage code deposition in a community repository (e.g. GitHub). See the Nature Portfolio guidelines for submitting code & software for further information.

Data

Policy information about availability of data

All manuscripts must include a data availability statement. This statement should provide the following information, where applicable:

- Accession codes, unique identifiers, or web links for publicly available datasets
- A description of any restrictions on data availability
- For clinical datasets or third party data, please ensure that the statement adheres to our policy

The accession numbers for the raw data used for genomic survey are provided in Supplementary Table 1 and Supplementary Table 15. The raw sequencing data (PacBio HiFi, ONT, Hi-C, and short reads) for S. younghusbandi and S. curvilabiatus were deposited in the SRA database under the corresponding accession numbers PRJNA1210471 and PRJNA1394917. The raw data of RNA-seq generated in this study can be found in NCBI under accession PRJNA1402086. The four assembled haplotypes of the S. curvilabiatus genome can be accessed via the ENA under the accession numbers GCA_978021885.1, GCA_978021895.1, GCA_978021905.1, and GCA_978021915.1. The assembled S. younghusbandi genome can be found in Figshare (doi.org/10.6084/m9.figshare.31700350). The annotation files can be found in Figshare (doi.org/10.6084/m9.figshare.28468799 and doi.org/10.6084/m9.figshare.31101751). Raw imaging data of chromosome spreading can be found in Figshare (doi.org/10.6084/m9.figshare.31143715). The BUSCO database used in this study is available at https://busco-data.ezlab.org/v5/data/lineages.

Research involving human participants, their data, or biological material

Policy information about studies with human participants or human data. See also policy information about sex, gender (identity/presentation), and sexual orientation and race, ethnicity and racism.

Reporting on sex and gender	N/A
Reporting on race, ethnicity, or other socially relevant groupings	N/A
Population characteristics	N/A
Recruitment	N/A
Ethics oversight	N/A

Note that full information on the approval of the study protocol must also be provided in the manuscript.

Field-specific reporting

Please select the one below that is the best fit for your research. If you are not sure, read the appropriate sections before making your selection.

☒ Life sciences ☐ Behavioural & social sciences ☐ Ecological, evolutionary & environmental sciences

For a reference copy of the document with all sections, see nature.com/documents/nr-reporting-summary-flat.pdf

Life sciences study design

All studies must disclose on these points even when the disclosure is negative.

Sample size	Six biological replicates meet the statistical power requirements of most RNA-seq experiments, and only one individual for each species was used for genome sequencing.
Data exclusions	No data exclusions.
Replication	Six biological replicates were used for the RNA-seq data, with one condition (5°C, heart) having five replicates; biological replicates are not a standard requirement for other experiments and genome sequencing in this study.
Randomization	In the RNA-seq experiment, juvenile fish were randomly assigned to the control and experimental groups; the other genome sequencing studies in this work do not involve randomization.
Blinding	Blinding was not used to in this study. All of experiment group are not involved in data analysis process. Results are derived from objective data processed by bioinformatics software and are therefore not influenced by investigator.

Reporting for specific materials, systems and methods

We require information from authors about some types of materials, experimental systems and methods used in many studies. Here, indicate whether each material, system or method listed is relevant to your study. If you are not sure if a list item applies to your research, read the appropriate section before selecting a response.

Materials & experimental systems

n/a	Involved in the study
☒	☐ Antibodies
☒	☐ Eukaryotic cell lines
☒	☐ Palaeontology and archaeology
☐	☒ Animals and other organisms
☒	☐ Clinical data
☒	☐ Dual use research of concern
☒	☐ Plants

Methods

n/a	Involved in the study
☒	☐ ChIP-seq
☒	☐ Flow cytometry
☒	☐ MRI-based neuroimaging

Animals and other research organisms

Policy information about studies involving animals; ARRIVE guidelines recommended for reporting animal research, and Sex and Gender in Research

Laboratory animals

Juvenile S. younghusbandi were provided by the College of Fisheries at Southwest University and reared in aquaculture facilities. When the juveniles reached approximately one year of age, with a body length of about 80 mm and a weight of approximately 4 g, and before their sex was determined, they were used in the experiment. The tanks (diameter: 80 cm, volume: 300 L) were maintained at 16°C with a light/dark cycle of 12 hours/12 hours, a water flow rate of 0.286 L/s, and dissolved oxygen >8 mg/L. These juveniles were randomly divided into a control group and an experimental group. The juveniles used in the experiment (both the control and experimental groups) were euthanized using an overdose of MS222, and all tissues were collected. Animal carcasses were disposed of by a professional company. This experiment was approved by the Institutional Animal Care and Use Committee (IACUC) of Southwest University.

Wild animals

Aspiorhynchus laticeps, Gymnodiptychus pachycheilus, Ptychobarbus dipogon, Chuanchia labiosa, Platypharodon extremus, and Herzensteinia microcephalus (one individual each) were collected from Xinjiang, Gansu, and Tibet (Xizang) provinces of China. The collection of these specimens was conducted with local sampling permits. The age and sex of these samples were unknown. Because it was not possible to rear them in the field, and to prevent DNA degradation, the animals were processed and sampled immediately after capture. The fish were euthanized by over-anesthetization with MS222, and various tissues were collected and preserved using dry ice.
One adult female and one adult male Schizopygopsis younghusbandi were collected from Yarlung Tsangpo River and placed in transportation bags filled with oxygen. The fish were transported to the fish culture facility at Southwest University. The tanks (80 cm diameter, 300 L volume) were maintained at 16°C with a 12-h light/12-h dark cycle, a water flow of 0.286 L/s, and dissolved oxygen >8 mg/L. Artificial induction of spawning and fertilization were performed according to standard technical procedures to obtain fertilized eggs. After hatching and subsequent growth, the juveniles were euthanized according to the protocol described above, and tissues were collected and preserved. The parent fish were euthanized and tissues were collected in the same manner. All animal experiments were approved by the Institutional Animal Care and Use Committee (IACUC) of Southwest University. Animal carcasses were disposed of by a professional company.

Reporting on sex

Except for the parent fish used for artificial reproduction, no sex information was determined for the other animals.

Field-collected samples

No field-collected samples were used.

Ethics oversight

The method used for animal euthanasia was MS-222 overdose anesthesia. The procedure was as follows: a high-concentration aqueous solution of MS-222 (>250 mg/L) was prepared, and the experimental animals were transferred into the overdose solution. Individuals were immersed for 10 minutes, and after complete cessation of opercular movement, immersion was continued for an additional 5–10 minutes. Before sampling, a secondary physical confirmation of death was performed by destruction of the brain (e.g., rapid decapitation) to ensure complete euthanasia. This procedure ensures that the animals remained under deep anesthesia throughout the entire process and experienced no pain or distress, in accordance with animal welfare and ethical standards.

Note that full information on the approval of the study protocol must also be provided in the manuscript.

Plants

Seed stocks

N/A

Novel plant genotypes

N/A

Authentication

N/A
