# Supplementary material for: Chromosomal fusions trigger rediploidization of autopolyploid genomes
Source: Nature. 2026 Apr 22;654(8119):706–13. doi: 10.1038/s41586-026-10439-1 (PMC13275295; doi:10.1038/s41586-026-10439-1)
Supplement: Supplementary file 4 — Peer Review File [file 41586_2026_10439_MOESM4_ESM.pdf]

nature portfolio

Peer Review File

Chromosomal fusions trigger rediploidization of autopolyploid genomes

Corresponding Author: Professor Axel Meyer

Any redactions in this file are there to maintain patient confidentiality, the confidentiality of unpublished data, or to remove third-party material.

This file contains all reviewer reports in order by version, followed by all author rebuttals in order by version.

Version 1:

Reviewer comments:

Referee #1

(Remarks to the Author)
The manuscript "Chromosomal fusions trigger genome rediploidization in a young autopolyploid vertebrate" by Xie et al., presents a thorough analysis of whole genome duplication events in a clade of carps. It convincingly shows the autopolyploid origin of the initial duplication events, and provides an unprecedented view of the ongoing process of rediploidization, highlighting the key role that chromosomal fusions play in this process. Overall, the manuscript is well-written and balanced, and the figures are particularly elegant and easy to follow, despite the complexity of the subject. We are positive about this manuscript, provided the author address the following list of points.

Major points:

Our only major points concern the analysis of divergent expression patterns (Figure 5c-g). We have several concerns with these:

• Fig5d: Apparently, it contains data concerning one tissue only (skin) out of the 29 tissues analyzed. Why is this? If there is a reason to use skin, it should be noted. Otherwise, why not aggregate all tissues together in the same figure? It would not be more complex, but would better reflect reality.

• Fig5e suggests that out of 29 tissues, 27 show a consistent bias towards expression of ‘uf’ copies, while 2 others show an inverse pattern. It would be very interesting to know what these 2 outliers are, and to comment on why this may be. More generally, it would be useful to add the names of the tissues (or just a 1-2-letter symbol) next to the dots in order to make the figure more readable.

• We feel that the conclusion that expression imbalance between ohnologues is primarily caused by the repression of one ohnologue (as opposed to, for example, the upregulation of the other), is poorly supported. This is actually reflected in the convoluted phrasing of the paragraph itself (see minor points below).
As far as we understand, the only argument supporting this claim is that among the pairs of unbalanced ohnologues, the ‘most expressed’ ohnologues have a distribution of expression levels closer to that of ‘balanced’ ohnologues, while the ‘least expressed’ ohnologues tend to have markedly lower expression levels than the ‘balanced’ ohnologues (Fig 5f). This conclusion seems however very fragile. First, because, it is not really true: for example, uf-biased genes (which make a majority of the biased genes) have stronger expression levels than ‘balanced’ genes. This point is even mentioned in the paragraph, which is confusing since it somehow contradicts the claimed result. Second, and most importantly, it is probably not conclusive to compare biased genes with balanced genes: they make two distinct populations, and it is highly probable that they differ in many biological aspects, including their range of expression. Therefore, a more rigorous way to do such comparisons would be to compare the expression of ohnologues with the expression of their non-duplicated orthologue in a related species (ideally, in a comparable tissue). In these conditions only would it make sense to claim that each ohnologue expression is enhanced vs. repressed compared to the orthologue reference, similar to what was done, for example, in the following study:
Braasch, I. et al. The spotted gar genome illuminates vertebrate evolution and facilitates human-teleost comparisons. Nat.

Genet. 48, 427–437 (2016).
We understand that such data may not be easily exploitable, but in their absence, we think one should be much more cautious about the conclusion. Especially given that this particular result (the repression of one ohnologue) is not central to the manuscript claims.

• Finally, while the cold-induction experiment is very interesting its principle, we feel its results are not clearly rendered in the text. It seems that the main result is negative: there is no bias towards or against ohnologues among DEGs. While this may be disappointing, it should be clearly stated as such.
The case of GPX4, however, is too speculative to be worth mentioning, and certainly does not deserve the term of “strong candidate”. There are hundreds of DEGs on the volcano plot of Fig.5g. It would at least be interesting to see which one is GPX4 among those, in order to know whether it is a strong outlier, or just one among hundreds of others. As far as we can tell from the data shown in the manuscript, there is almost nothing to support a particular role for GPX4 in these carps, besides what is already known in the literature. This result should be either supported by much more statistical and experimental evidence, or dropped altogether.

Minor points:

• L180: “a plethora” should be replaced by the number of actual trees that were computed, even an approximate number.

• l 211: typo: stable

• Figure 4e: I do not understand the difference between A and B chromosomes? B seems to be the fused version, but this is not explicit.

• L264-268: The conclusion that “Centromere repositioning may have also contributed to rediploidization” would be better supported if it was shown that the distinct inheritance patterns between chr17 long arm and short arm are specific to this chromosome, i.e. that there is no difference in inheritance patterns between short and long arms in other acrocentric chromosomes. Furthermore, since the repositioning of the centromere in chr17 is caused by an inversion, it is not clear whether the loss of recombination between the 2 copies is caused primarily by the displacement of the centromere rather than by the inversion itself.

• L. 276: “Those gene losses can be categorized into deletion polymorphisms caused by polysomic masking and true gene losses occurring post-rediploidization, where the latter are expected to occur exclusively either unfused or fused copies”.
It may be useful to define the term polysomic masking here, since it is not of common usage and does not seem to be clearly defined in the cited reference. In addition, it seems to us that deletion polymorphisms and true gene losses are 2 successive steps of the same process, the latter resulting from the genetic fixation of the former. While gene loss is indeed associated to rediploidization, since it precludes recombination between the divergent copies, it could in theory occur without the need of fusion events. We may have missed some crucial details, though, so maybe this section needs more information.

• L282 and Figure 5b: The bias of ohnolog gene loss towards the fused copy is so conspicuous in the figure that it is hard to think that the p-value of the null hypothesis be so unremarkable as p=0.03. Rather than applying a Wilcoxon statistical test on just 5 cases (= the 5 fused chromosomes) and measuring the statistical significance of the fused (blue) being always higher than the unfused (orange), wouldn’t it be more appropriate (and certainly more statistically significant) to consider each gene loss as an independent event (i.e. n = 360 gene losses), and use a test (e.g. chi-square) that measures the statistical significance of the deviation evidenced by the 257 ‘fused’ gene losses vs 103 non-fused gene losses, against the null hypothesis that each loss may occur equally on the fused or unfused copy ? We think this would make the result stand out in its right proportion.

• L283-285 “This biased loss similar to observations of subgenome dominance proposed to be the hallmark of allopolyploids.”
What do the authors want to convey with this sentence? While it is true that the same kind of biased gene loss is also observed upon subgenome dominance in allopolyploids, it is a bit misleading to mention this in the Results section, since what is being studied here is precisely not an allopolyploidy, therefore we are not dealing with a process of subgenome dominance per se. Hence, the sentence does not make an appropriate conclusion for the paragraph. Plus, it tends to confuse the reader about the nature of the carp polyploidy.
This observation would be more appropriately moved to the Discussion section, where it could be highlighted as a caveat for people studying WGDs: some properties of the rediploidization mechanism described here mimic subgenome dominance in allopolyploids, meaning that care must be taken when drawing conclusions on allo- vs. auto-polyploidy on that sole basis.

• L288 “ohnolog pairs exhibiting clear bifurcating structures”
Could the authors please be more explicit as to what they mean by exhibiting clear bifurcating structures ? The phrase is too elliptic for the average reader.

• L298 We recommend that the authors not use the term “suppression” here, since it may apply both to gene deletion, which was studied just above, or to repression of expression, which is the case here. It would be clearer to use the more precise term “repression’.

• L298-301 These 2 sentences seem grammatically incorrect (lack of verbs).
See also the major point above about the conclusions of this paragraph, which need some refinement.

• L305-306 “A total of 637 and 2,759 differentially expressed gene (DEGs) were identified, respectively (Fig. 5g, Supplementary Table 12), including (10%) and 274 (10%) ohnologs”
If 80% of genes are retained in 4 copies (l. 274), how can there be only 10% ohnologues among the DEG genes? This appears like a striking under-representation. Or, more likely, some detail has escaped our attention, which suggests the results should be more clearly stated.

• L315: The sentence containing “ 5.2 – 17.9 million heterozygous tetraploid SNPs” should be more explicit (e.g. between 5.2 and 17.9 million”, stating that this is a range of counts in . Also it would be good to define “tetraploid SNP”. Most readers would be familiar with diploid genomes where SNPs can be bi-allelic or tri-allelic, or multi-allelic (three or more), counting the reference as one and additional SNPs allowing for additional variants. I am not sure myself what a tetraploid SNP is.

• Fig. 2d: how could the authors measure f(p) vs f(p)? there may be a typo here?
• Fig.2e: Chromosome 14 (?) is incorrectly labelled as ‘41’.

Referee #2

(Remarks to the Author)
I do not have any major technical critique of the methods used in this manuscript. The genome assembly, orthology and chromosomal comparisons are standard and well executed. Validation via FISH is an important piece of data that goes along with the HiC assembly.
To me, the overall interpretation of a shared ancestral polyploidization, followed by a fusion can be logical from the syntenic point of view. However, it is not clearly visible from the figures or data. It would be beneficial to show homologies of m1/m2/p1/p2 across snow carp lineages to highlight that these chromosomes are separate yet conserved units across multiple species. I think a similar plot as in Supplementary Figure 7 would be beneficial to show for a selection of species in the main text, to highlight the chromosomal retention after WGD (which is followed by fusion in one or some species). The logic for the inference of a shared chromosomal (!) WGD is unclear to me otherwise.
Assuming that ancestral and shared WGD event is true and can be confirmed by synteny across multiple carp species, I would then still recommend to take the alleged conflicting result that the authors mention with a bit of nuance. That study did not look at chromosomal synteny or conservation and it is not uncommon that gene genealogies are rather noisy in whole genome duplication timing reconstructions (this is actually my concern with the SNP-based results described in the section "Snow carp likely share a single autopolyploid event"). In this context, I would suggest to be explicit about the limitation of the study in reference 22. More generally, a single shared polyploidization event seemed to be anyways the most parsimonious explanation in multiple publications, so it would be good to state so (if true).
I personally do not find it surprising that fused chromosomes show higher rates of rediploidization since selection would act against increase in dosage, especially if ohnologs suddenly become located on a single chromosome. It is certainly very good to have such example investigated in this paper, but can the authors be more explicit what the alternative would be or if any mechanism can be imagined that would keep double dosage of genes on recently fused chromosomes?
Finally, it is unclear why there are (or should be) three stages of rediploidization in younghusbandi. What is the evidence that these fusions happened sequentially and is not some phylogenetic dating artefact? Can the authors try to put the different chromosomal fusions along the phylogeny (here again it would be beneficial to compare to other related carp species with synteny and not duplicate dating) to see if certain sets of fusions happened at particular speciation times (or not). Maybe I can see a clear Ks difference between Stages 3 and 2, but stages 1 and 2 seem to be more similar to each other.

Referee #3

(Remarks to the Author)
Xie and colleagues present here a model for the molecular mechanism underlying early steps of rediploidization after autotetraploidy, utilizing snow carp species as a model system. They have generated a high-quality, chromosome-level, haplotype-resolved genome of the species *Schizopygopsis younghusbandi* and generated genomic data, including Hi-C data, for other 10 species. They have found that fusion among different chromosomes sparks early rediploidization, explaining in part why the process is asynchronous.

The assembly strategy for *S. younghusbandi* is quite smart and I appreciate the efforts put by the authors to generate such a resource. The gene analyses done in terms of their divergence and disomic versus tetrasomic inheritance across the genome, taking into account the ohnology relationships between 90 chromosomes (!!) is very elegant. The statistical analyses seem correct to me, well justified and well presented in all cases. Overall, I think this is one of the most outstanding studies centered on understanding the mechanisms of early rediploidization after WGD in vertebrates, especially underlying asynchronous rediploidization. No doubt this study will inspire many other works focusing on different taxa in order to replicate these results. In this regard, I support its publication mainly as it is, I do not think it requires, in my humble opinion, any major changes.

I do have, nonetheless, some general questions or comments, particularly regarding the interpretation of the results. They mention that after the vertebrate ancestral 1R WGD event, 9 chromsomal fusions have been inferred (actually some models infer 8). In this regard, although rediploidization at fusion sites might be general, it is noteworthy to mention that not all ancestral chromosomes undergo fusion events but end up being rediploidized later in evolution. Therefore, it seems it could be a mechanism that could accelerate the process, but not the one that explains a complete rediploidization of a genome. Do the authors have any thoughts on this?

My second question concerns the evolutionary implications of rediploidization, particularly in its early stages, for phenotypic diversification or adaptive radiation in the group. If I understood correctly, the authors suggest that the chromosomal fusion events and the initial rediploidization observed could be linked to the adaptation of the specialized+highly specialized group to extremely cold environments. However, at present there are no fusion events that appear to be synapomorphic for the specialized+highly specialized clade. Without such shared derived changes, establishing a causal relationship between these fusions and the adaptive evolution of snow carps remains, in my humble opinion, speculative, and the proposed link seems based solely on a correlation. Addressing this would likely require stronger functional evidence (their RNA-seq DE analysis, for instance, mostly fails to recover genes associated with cold adaptation) together with clearer phylogenetic support. I do not state this as a criticism of the study, but rather as a spark for a stimulating and constructive discussion!

I have some other minor comments for the authors listed below:
1) L73, L272: Apparently, and at least after ancient WGD events in both gnathostomes and cyclostomes, specialization is a more common fate for duplicates than neofunctionalization or subfunctionalization. See Marlétaz et al., 2018 (https://doi.org/10.1038/s41586-018-0734-6) and Yu et al., 2024 (reference 9)

2) L73: add “likely” to the statement “promoting phenotypic innovation” to be in line with previous mentions of the potential of WGD events in generating diversification and facilitating evolution of novelty. The causal link between WGDs and their consequences in phenotype evolution are not so clear as I suggest above (see also Yu et al., 2024, reference 9, for an example about cyclostome evolution).

3) Change “basal group” to “non-specialized group”: The use of the term “basal” to define one of the snow carp groups is incorrect. A cladogram of extant species just shows the phylogenetic relationships among them, and as such, it does not tell us if an extant clade is basal or not, but instead which it is the sister group to. In this case, what the authors call the “basal” group is actually the sister (species poor) group of the specialized+highly specialized clade. Calling the Aspiorhynchus+Schizothorax clade a “basal group” implies some connotations of a ladder of progress when reading the tree, when in reality both derived from a common ancestor (which is basal to both of them). I suggest therefore to change all instances of basal, in the text and figures, to the more accurate name of “non-specialized” to refer to the group given the context used by the authors.

4) Legend of Supplementary Fig. 7. Add “each” to the sentence for clarity: “10 chromosomes correspond each to two chromosomes in O. macrolepis”.

5) Naming the stages of rediploidization from III to I sounds counterintuitive. I think it makes more sense in reverse

6) There is a typo in Fig. 2e, it should be 14 instead of 41.

7) x axis labels for Fig. 4e seems incorrect on the disomic chromosomes?

8) Figure 6 legend: e and d are inverted: d represents a single WGD for snow carps, and e multiple WGDs.

Version 2:

Reviewer comments:

Referee #1

(Remarks to the Author)

Referee #2

(Remarks to the Author)
The authors have addressed my comments and provided new data and validations, which satisfactory address my questions on WGD timing and chromosomal-fusion history. What I still strongly recommend to adjust is the discussion of the previous study that claims multiple rounds of polyploidization. I think the current wording is unnecessarily confrontational in the sentence "This strongly refutes a recent study that concluded the existence of multiple independent...". I think this should be changed to rather highlight the actual qualitative difference between the two studies, i.e. the chromosomal-scale data and comparisons.
I have one last suggestion: in figure 2 (and maybe others like EDF8), I suggest to color the homology lines by chromosomal identity of unfused chromosomes (19 one color, 22 another color etc). This will allow to see if the fused chromosomes have

undergone any mixing through translocations across the centromere, which would further solidify their shared derived (and irreversible) state. See for example reference 56 for a few examples for the ancient vertebrate WGDs and the fusion-with-mixing concept and its implications. In the alternative, if these fusions are not majorly mixed yet across the centromere (as I would naively expect given the divergence time), then the fusions could in theory revert back and therefore its good to show several such shared fusions, as the authors do. In any case, the reference 56 is probably good to include in any discussion of the results that suggest impact of chromosomal fusions on rediploidizations after vertebrate WGDs.

Referee #3

(Remarks to the Author)
I am pleased to see that the authors found my suggestions, as well as those of other reviewers helpful. I appreciate the additional effort invested in strengthening the manuscript, particularly providing a haplotype resolved genome assembly for a second carp species, and extending their transcriptomics analyses.

As I mentioned previously, I think this is an impressive piece of work that represents a significant advance in our understanding of the genome evolutionary processes following whole genome duplication events.

Open Access This Peer Review File is licensed under a Creative Commons Attribution 4.0 International License, which permits use, sharing, adaptation, distribution and reproduction in any medium or format, as long as you give appropriate credit to the original author(s) and the source, provide a link to the Creative Commons license, and indicate if changes were made.
In cases where reviewers are anonymous, credit should be given to 'Anonymous Referee' and the source.
The images or other third party material in this Peer Review File are included in the article’s Creative Commons license, unless indicated otherwise in a credit line to the material. If material is not included in the article’s Creative Commons license and your intended use is not permitted by statutory regulation or exceeds the permitted use, you will need to obtain permission directly from the copyright holder.
To view a copy of this license, visit https://creativecommons.org/licenses/by/4.0/

Referee #1 (Remarks to the Author):

The manuscript "Chromosomal fusions trigger genome rediploidization in a young autopolyploid vertebrate" by Xie et al., presents a thorough analysis of whole genome duplication events in a clade of carps. It convincingly shows the autopolyploid origin of the initial duplication events, and provides an unprecedented view of the ongoing process of rediploidization, highlighting the key role that chromosomal fusions play in this process. Overall, the manuscript is well-written and balanced, and the figures are particularly elegant and easy to follow, despite the complexity of the subject. We are positive about this manuscript, provided the author address the following list of points.

R: Thank you for the positive comments and your constructive suggestions to improve the manuscript. We address all of your points in turn below.

Major points:

Our only major points concern the analysis of divergent expression patterns (Figure 5c-g). We have several concerns with these:

• Fig5d: Apparently, it contains data concerning one tissue only (skin) out of the 29 tissues analyzed. Why is this? If there is a reason to use skin, it should be noted. Otherwise, why not aggregate all tissues together in the same figure? It would not be more complex, but would better reflect reality.

R: In the revised manuscript, we have generated new RNA-seq data from eleven tissues of *S. younghusbandi* to standardize our comparisons and limit batch effects. The RNA-seq from eleven tissues was pooled from multiple individuals of similar ages to average out individual variations to ensure the RNA-seq datasets were highly suitable for transcriptomic analysis.

The original Fig. 5d (Fig. 5c in revised version) has been revised to illustrate more intuitively the expression levels of the *uf* and *f* copies. In the Fig. 5c, data from all tissues are aggregated into a single panel, and we also provide separate panels for all eleven tissues in the Supplementary Fig. 14.

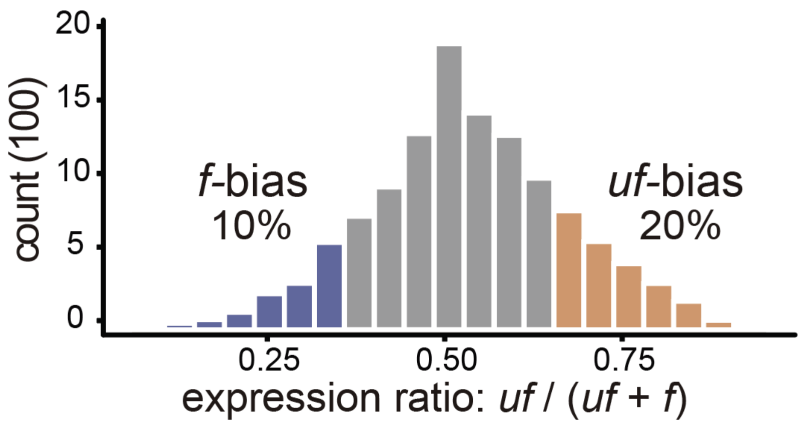

Fig. 5c

• Fig5e suggests that out of 29 tissues, 27 show a consistent bias towards expression of ‘uf’ copies, while 2 others show an inverse pattern. It would be very interesting to know what these 2 outliers are, and to comment on why this may be. More generally, it would be useful to add the names of the tissues (or just a 1-2-letter symbol) next to the dots in order to make the figure more readable.

R: Thank you for your suggestions here. In the revised manuscript, RNA-seq datasets from public databases are no longer used, because those datasets were collected from different research groups with varying quality. Instead, we generated RNA-seq data from eleven tissues using the same pooled set of multiple individuals, allowing us to revisit this question with a standardized dateset. Our new results show a consistent pattern, indicating that the uf-bias pattern holds in all eleven tissues. The detailed figures are included in Supplementary Fig. 14.

• We feel that the conclusion that expression imbalance between ohnologues is primarily caused by the repression of one ohnologue (as opposed to, for example, the upregulation of the other), is poorly supported. This is actually reflected in the convoluted phrasing of the paragraph itself (see minor points below).

R: We understand your concern. We agree that without RNA-seq data from a closely related diploid species, we have been unable to determine the direction of change of gene expression. As this point is not central to the main conclusions of the study, we have removed this potentially misleading statement from the revised manuscript.

As far as we understand, the only argument supporting this claim is that among the pairs of unbalanced ohnologues, the ‘most expressed’ ohnologues have a distribution of expression levels

closer to that of ‘balanced’ ohnologues, while the ‘least expressed’ ohnologues tend to have markedly lower expression levels than the ‘balanced’ ohnologues (Fig 5f). This conclusion seems however very fragile. First, because, it is not really true: for example, uf-biased genes (which make a majority of the biased genes) have stronger expression levels than ‘balanced’ genes. This point is even mentioned in the paragraph, which is confusing since it somehow contradicts the claimed result. Second, and most importantly, it is probably not conclusive to compare biased genes with balanced genes: they make two distinct populations, and it is highly probable that they differ in many biological aspects, including their range of expression. Therefore, a more rigorous way to do such comparisons would be to compare the expression of ohnologues with the expression of their non-duplicated orthologue in a related species (ideally, in a comparable tissue). In these conditions only would it make sense to claim that each ohnologue expression is enhanced vs. repressed compared to the orthologue reference, similar to what was done, for example, in the following study:

Braasch, I. et al. The spotted gar genome illuminates vertebrate evolution and facilitates human-teleost comparisons. Nat. Genet. 48, 427–437 (2016).

We understand that such data may not be easily exploitable, but in their absence, we think one should be much more cautious about the conclusion. Especially given that this particular result (the repression of one ohnologue) is not central to the manuscript claims.

R: Thank you for raising this important concern and recommending an improved strategy to strengthen our approach for inferring the ohnolog expression changes. We agree that adequately addressing this issue would require the inclusion of RNA-seq data from closely related diploid species with matched tissues, which has been unfortunately not available. In order not to further distract the manuscript, we decided to streamline this part and focused more on the chromosome behavior of snow carps during meiosis. In fact, we have collected more meiotic karyotypes with both centromeres and telomeres targeted for FISH experiments, providing stronger evidence for tetravalent pairing.

• Finally, while the cold-induction experiment is very interesting its principle, we feel its results are not clearly rendered in the text. It seems that the main result is negative: there is no bias towards or against ohnologues among DEGs. While this may be disappointing, it should be clearly stated as such.

R: We have re-designed the cold-induction experiment (22℃ *vs.* 5℃) and performed RNA-seq on five tissues, with six biological replicates for each condition (Fig. 5d and Extended Data Fig.

6b-d). The results showed a significant enrichment of ohnologs among DEGs in the liver and heart (odds ratios = 1.23 and 1.21, respectively), but no enrichment was detected in the other tissues (Supplementary Table 13). The new analyses were consistent with our previous analyses, and because we thought these analyses did not provide strong evidence for an adaptive role of ohnolog expression divergence, we have further toned down the causal relationship between rediploidization and adaptation.

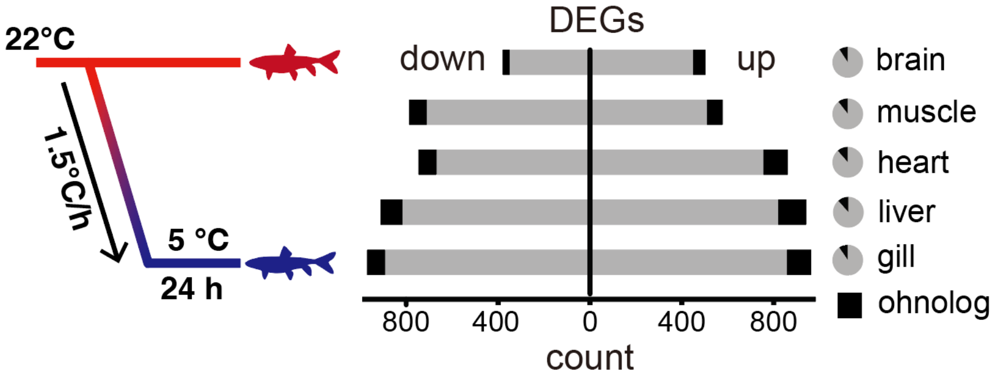

Fig. 5d

The case of GPX4, however, is too speculative to be worth mentioning, and certainly does not deserve the term of "strong candidate". There are hundreds of DEGs on the volcano plot of Fig.5g. It would at least be interesting to see which one is GPX4 among those, in order to know whether it is a strong outlier, or just one among hundreds of others. As far as we can tell from the data shown in the manuscript, there is almost nothing to support a particular role for GPX4 in these carps, besides what is already known in the literature. This result should be either supported by much more statistical and experimental evidence, or dropped altogether.

R: Due to the weak support for the role of *GPX4* in low-temperature adaptation in snow carps, we have removed this statement from the revised manuscript, as per your advice.

Minor points:

• L180: "a plethora" should be replaced by the number of actual trees that were computed, even an approximate number.

R: The text has been revised to "27,049 phylogenetic trees".

• l 211: typo: stable

R: Fixed.

• Figure 4e: I do not understand the difference between A and B chromosomes? B seems to be the fused version, but this is not explicit.

R: Thank you for pointing this out. In the revised manuscript, we used the unfused (*uf*) and the fused (*f*) to differentiate the homologous chromosomes.

• L264-268: The conclusion that "Centromere repositioning may have also contributed to rediploidization" would be better supported if it was shown that the distinct inheritance patterns between chr17 long arm and short arm are specific to this chromosome, i.e. that there is no difference in inheritance patterns between short and long arms in other acrocentric chromosomes. Furthermore, since the repositioning of the centromere in chr17 is caused by an inversion, it is not clear whether the loss of recombination between the 2 copies is caused primarily by the displacement of the centromere rather than by the inversion itself.

R: This is a great suggestion. We added a supplementary figure (Supplementary Fig. 12, also shown below) illustrating the Ks patterns of genes on short and long chromosome arms across all non-fused tetrasomic quartets. The right arm of chr17 shows a higher divergence level between the two haplotypes. We agree that inversions, instead of centromeric repositioning, are more likely the cause of rediploidization. We have now clarified the writing:

"In the short arm of chr17 involving a centromeric inversion, a rediploidized pattern might be indicative of disomic inheritance, while the long arm showed a tetrasomic inheritance pattern (Extended Data Fig. 3f). This rediploidization pattern is not observed in other acrocentric chromosomes without centromeric inversions (Supplementary Fig. 12), indicating an association between rediploidization and centromeric inversions."

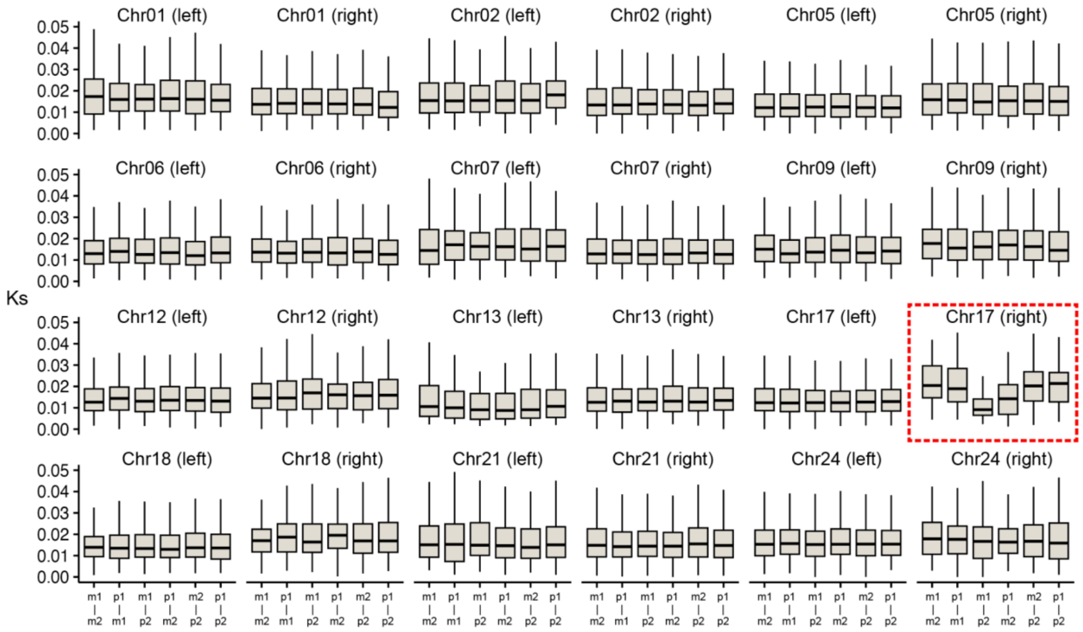

Supplementary Figure 12, only shows unfused quartets

• L. 276: "Those gene losses can be categorized into deletion polymorphisms caused by polysomic masking and true gene losses occurring post-rediploidization, where the latter are expected to occur exclusively either unfused or fused copies".

It may be useful to define the term polysomic masking here, since it is not of common usage and does not seem to be clearly defined in the cited reference. In addition, it seems to us that deletion polymorphisms and true gene losses are 2 successive steps of the same process, the latter resulting from the genetic fixation of the former. While gene loss is indeed associated to rediploidization, since it precludes recombination between the divergent copies, it could in theory occur without the need of fusion events. We may have missed some crucial details, though, so maybe this section needs more information.

R: Thank you very much for your comment. We modified the diagram to illustrate ohnolog loss; other types of loss are referred to as allele loss. To more clearly define ohnologous gene losses, we have rephrased the sentence:

"We further focused on ohnolog loss (Fig. 5a), referring to genes absent from either the uf or f chromosomes in disomic quartets, and identified 360 such events." We hope that the writing is clearer now.

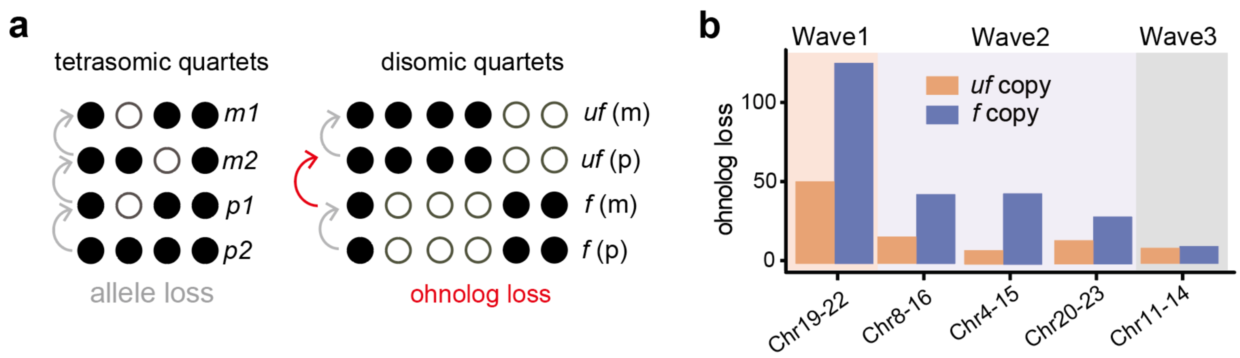

Fig. 5a-b

• L282 and Figure 5b: The bias of ohnolog gene loss towards the fused copy is so conspicuous in the figure that it is hard to think that the p-value of the null hypothesis be so unremarkable as p=0.03. Rather than applying a Wilcoxon statistical test on just 5 cases (= the 5 fused chromosomes) and measuring the statistical significance of the fused (blue) being always higher than the unfused (orange), wouldn't it be more appropriate (and certainly more statistically significant) to consider each gene loss as an independent event (i.e. n = 360 gene losses), and use a test (e.g. chi-square) that measures the statistical significance of the deviation evidenced by the 257 'fused' gene losses vs 103 non-fused gene losses, against the null hypothesis that each loss may occur equally on the fused or unfused copy ? We think this would make the result stand out in its right proportion.

R: Thank you for your helpful suggestion regarding statistical analysis. We have now performed a chi-square test, and the results indicate that ohnolog loss occurs significantly more frequently in the f copy (p-value< 0.01)

• L283-285 "This biased loss similar to observations of subgenome dominance proposed to be the hallmark of allopolyploids."

What do the authors want to convey with this sentence? While it is true that the same kind of biased gene loss is also observed upon subgenome dominance in allopolyploids, it is a bit misleading to mention this in the Results section, since what is being studied here is precisely not an allopolyploidy, therefore we are not dealing with a process of subgenome dominance per se. Hence, the sentence does not make an appropriate conclusion for the paragraph. Plus, it tends to confuse the reader about the nature of the carp polyploidy.

This observation would be more appropriately moved to the Discussion section, where it could be highlighted as a caveat for people studying WGDs: some properties of the rediploidization

mechanism described here mimic subgenome dominance in allopolyploids, meaning that care must be taken when drawing conclusions on allo- vs. auto-polyploidy on that sole basis.

R: Thank you for your suggested rewriting. We have clarified this and moved the rewritten sentence to the Discussion section.

• L288 “ohnolog pairs exhibiting clear bifurcating structures”

Could the authors please be more explicit as to what they mean by exhibiting clear bifurcating structures ? The phrase is too elliptic for the average reader.

R: We have rephrased “bifurcating structures” as “ohnolog pairs … were selected based on phylogenetic evidence for rediploidization”, which we feel will be easier for readers to digest.

• L298 We recommend that the authors not use the term “suppression” here, since it may apply both to gene deletion, which was studied just above, or to repression of expression, which is the case here. It would be clearer to use the more precise term “repression’.

R: This sentence has been partially removed and rewritten.

• L298-301 These 2 sentences seem grammatically incorrect (lack of verbs).

See also the major point above about the conclusions of this paragraph, which need some refinement.

R: This sentence has been partially removed and rewritten.

• L305-306 “A total of 637 and 2,759 differentially expressed gene (DEGs) were identified, respectively (Fig. 5g, Supplementary Table 12), including (10%) and 274 (10%) ohnologs”

If 80% of genes are retained in 4 copies (l. 274), how can there be only 10% ohnologues among the DEG genes? This appears like a striking under-representation. Or, more likely, some detail has escaped our attention, which suggests the results should be more clearly stated.

R: We performed DEG analysis using a single haplotype as the reference that had 25,023 genes. Accordingly, gene counts in DEGs are based on this single haplotype rather than the total number of genes across all four haplotypes. Although approximately 80% of genes retain four copies in the genome, ohnolog pairs represent only a subset of these, defined here as gene pairs exhibiting phylogenetic topologies such as ((uf, uf), (f, f)), and account for 9.8% (2,449 of 25,023) of all genes. In the revised manuscript, this statement has been clarified, and detailed statistics for DEG numbers and enrichment analyses are provided in Supplementary Table 13.

• L315: The sentence containing " 5.2 – 17.9 million heterozygous tetraploid SNPs" should be more explicit (e.g. between 5.2 and 17.9 million", stating that this is a range of counts in . Also it would be good to define "tetraploid SNP". Most readers would be familiar with diploid genomes where SNPs can be bi-allelic or tri-allelic, or multi-allelic (three or more), counting the reference as one and additional SNPs allowing for additional variants. I am not sure myself what a tetraploid SNP is.

R: We agree that "tetraploid SNPs" is potentially misleading, and now the sentence has been revised *as "We identified a range of 5.2 to 17.9 million heterozygous bi-allelic sites across nine additional species to S. younghusbandi (Supplementary Table 14)"*

• Fig. 2d: how could the authors measure f(p) vs f(p)? there may be a typo here?

R: typo fixed, thank you for spotting this.

• Fig.2e: Chromosome 14 (?) is incorrectly labelled as '41'.

R: typo fixed, thank you for spotting this.

Referee #2 (Remarks to the Author):

I do not have any major technical critique of the methods used in this manuscript. The genome assembly, orthology and chromosomal comparisons are standard and well executed. Validation via FISH is an important piece of data that goes along with the HiC assembly.

R: Thank you for your positive comments on our manuscript. In addition to Hi-C and karyotype analyses, we have now provided FISH results adding further support to the Chr19-Chr22 fusion (Extended Data Fig. 2c).

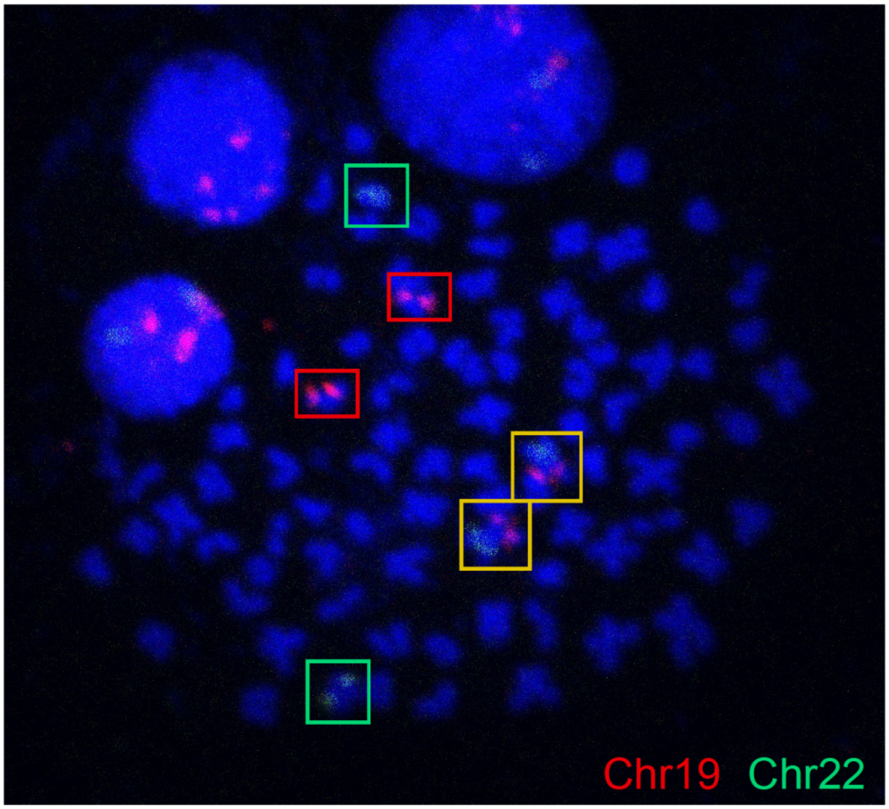

Extended Data Fig. 2c

To me, the overall interpretation of a shared ancestral polyploidization, followed by a fusion can be logical from the syntenic point of view. However, it is not clearly visible from the figures or data. It would be beneficial to show homologies of m1/m2/p1/p2 across snow carp lineages to highlight that these chromosomes are separate yet conserved units across multiple species. I think a similar plot as in Supplementary Figure 7 would be beneficial to show for a selection of species in the main text, to highlight the chromosomal retention after WGD (which is followed by fusion in one or some species). The logic for the inference of a shared chromosomal (!) WGD is unclear to me otherwise.

R: Thank you for your suggestion. To better illustrate the shared WGD, we have included a new haplotype-resolved genome assembly for a non-specialised snow carp *S. curvilabiatus* (Extended Data Fig. 8a,b). This species was chosen to provide maximal scope to confirm the inferred period of ancestral rediploidization in the common snow carp ancestor, given that the split between *S. curvilabiatus* and *S. younghusbandi* is the deepest speciation event in the group (Extended Data Fig. 8a). We added zebrafish as an outgroup to demonstrate the conserved synteny among cypriniformes genomes (Extended Data Fig. 8d). In the *S. curvilabiatus* genome, the chr19-22

fusion was clearly identified in synteny analysis (Extended Data Fig. 8b-d). This result adds strong independent support for an ancestral WGD followed by chromosomal fusion of chr19-22 before the divergence of *S. curvilabiatus* and *S. younghusbandi* (Fig. 6b, left), which fits our original conclusion that the chr19-22 fusion is the only genomic region that started rediploidization in the snow carp ancestor.

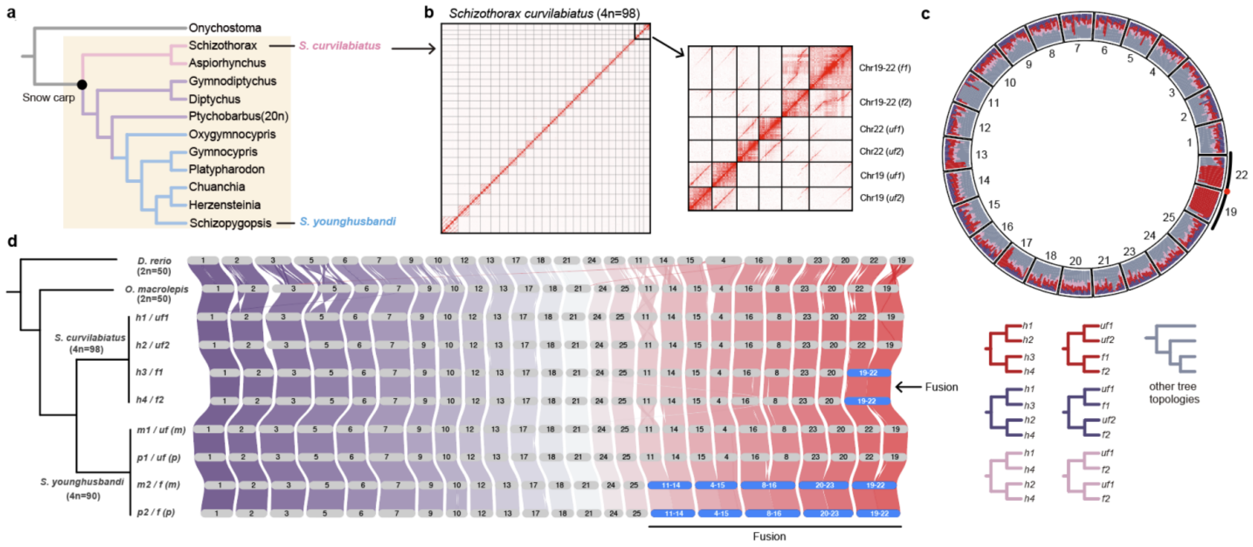

Extended Data. Fig 8

To strengthen our conclusions using the additional snow carp genome, we performed phylogenetic analyses of genes (or genomic regions) located in the rediploidized region of the chr19-22, capturing both species (Fig. 6c-d). The results captured ancestrally diverging ohnolog pairs (or genomic regions) shared by the two species (Fig. 6c, left), further supporting that the chromosomal fusion leading to rediploidization took place pre-speciation (Fig. 6b, left). Specifically, we observe a dominance of ancestrally diverging ohnolog pairs near the chr19-22 fusion site (Fig. 6d and Supplementary Fig. 15). This can only be explained if the stable disomic inheritance of unlinked ohnolog pairs began in the common ancestor to *S. curvilabiatus* and *S. younghusbandi* (i.e. ancestral rediploidization), which then continued in both lineages after speciation. It further supports our model of rediploidization being most advanced near fusion sites and provides a novel perspective of lineage-specific rediploidization (Extended Data Fig. 9).

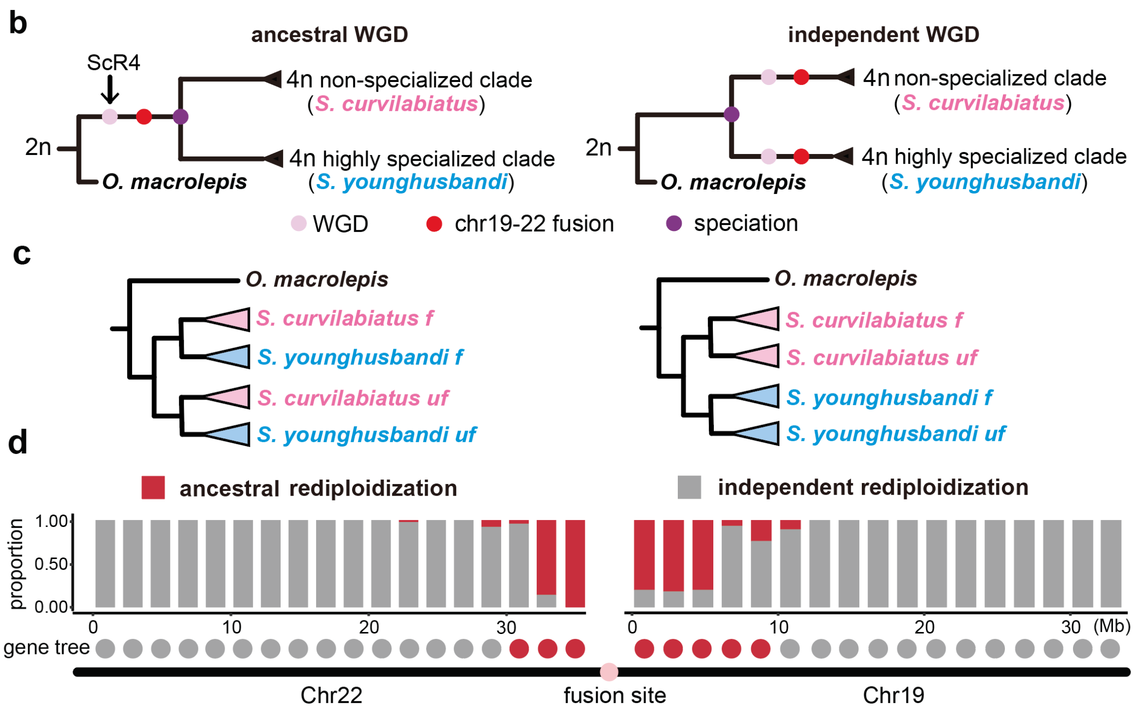

Fig. 6b-d

Assuming that ancestral and shared WGD event is true and can be confirmed by synteny across multiple carp species, I would then still recommend to take the alleged conflicting result that the authors mention with a bit of nuance. That study did not look at chromosomal synteny or conservation and it is not uncommon that gene genealogies are rather noisy in whole genome duplication timing reconstructions (this is actually my concern with the SNP-based results described in the section "Snow carp likely share a single autopolyploid event"). In this context, I would suggest to be explicit about the limitation of the study in reference 22. More generally, a single shared polyploidization event seemed to be anyways the most parsimonious explanation in multiple publications, so it would be good to state so (if true).

R: We fully agree with you that a shared polyploidization event provides the most parsimonious explanation. We have now added some discussion on the conflicting study:

"This strongly refutes a recent study that concluded the existence of multiple independent autopolyploidization events in snow carp evolution based on phylogenetic analysis of ohnologs without considering the possibility of asynchronous rediploidization and its expectations."

I personally do not find it surprising that fused chromosomes show higher rates of rediploidization since selection would act against increase in dosage, especially if ohnologs suddenly become located on a single chromosome. It is certainly very good to have such example investigated in

this paper, but can the authors be more explicit what the alternative would be or if any mechanism can be imagined that would keep double dosage of genes on recently fused chromosomes?

R: We would like to clarify that the rediploidization and gene loss rate are relatively slow. In chr19 and chr22, the chromosomal fusion occurs for two of the four haplotypes of chr19 and chr22, resulting in two unfused copies and two fused copies. The fused and unfused chromosomes have similar dosage for most genes, because most genes still retain four copies.

Finally, it is unclear why there are (or should be) three stages of rediploidization in younghusbandi. What is the evidence that these fusions happened sequentially and is not some phylogenetic dating artefact? Can the authors try to put the different chromosomal fusions along the phylogeny (here again it would be beneficial to compare to other related carp species with synteny and not duplicate dating) to see if certain sets of fusions happened at particular speciation times (or not). Maybe I can see a clear Ks difference between Stages 3 and 2, but stages 1 and 2 seem to be more similar to each other.

R: Thank you very much for the comment. We previously used “three stages” because different chromosomes have different ages of chromosomal fusions, thus varying degree of rediploidization. In the revised manuscript, we used three “waves” to refer to varying timing of rediploidization. We have now also included the genome of another snow carp (from the non-specialised group) in our analyses. We discovered that chr19-chr22 fusion (wave 1) was shared by these two species, supporting that this fusion was ancestral to all snow carps (as discussed above). For the wave 2 chromosomal fusions, they only occurred in *S. younghusbandi*, but not *S. curvilabiatus*, suggesting that those fusions were specific to the highly-specialised group.

Referee #3 (Remarks to the Author):

Xie and colleagues present here a model for the molecular mechanism underlying early steps of rediploidization after autotetraploidy, utilizing snow carp species as a model system. They have generated a high-quality, chromosome-level, haplotype-resolved genome of the species *Schizopygopsis younghusbandi* and generated genomic data, including Hi-C data, for other 10 species. They have found that fusion among different chromosomes sparks early rediploidization, explaining in part why the process is asynchronous.

The assembly strategy for *S. younghusbandi* is quite smart and I appreciate the efforts put by the authors to generate such a resource. The gene analyses done in terms of their divergence and disomic versus tetrasomic inheritance across the genome, taking into account the ohnology relationships between 90 chromosomes (!!) is very elegant. The statistical analyses seem correct to me, well justified and well presented in all cases. Overall, I think this is one of the most outstanding studies centered on understanding the mechanisms of early rediploidization after WGD in vertebrates, especially underlying asynchronous rediploidization. No doubt this study will inspire many other works focusing on different taxa in order to replicate these results. In this regard, I support its publication mainly as it is, I do not think it requires, in my humble opinion, any major changes.

R: We appreciate the reviewer's positive comments on our work.

I do have, nonetheless, some general questions or comments, particularly regarding the interpretation of the results. They mention that after the vertebrate ancestral 1R WGD event, 9 chromsomal fusions have been inferred (actually some models infer 8). In this regard, although rediploidization at fusion sites might be general, it is noteworthy to mention that not all ancestral chromosomes undergo fusion events but end up being rediploidized later in evolution.

Therefore, it seems it could be a mechanism that could accelerate the process, but not the one that explains a complete rediploidization of a genome. Do the authors have any thoughts on this?

R: Yes, we agree that our study suggests chromosome fusions only serve as a trigger of rediploidization at the early stage, and we have no intention to suggest that they are the only mechanism for all chromosomes at all stages of rediploidization. We now write in the discussion to clarify this:

"It should be noted that not all vertebrate ancestral chromosomes experienced fusions, implying that other mechanisms, such as inversions, may also contribute to genome rediploidization, as proposed in salmonids."

My second question concerns the evolutionary implications of rediploidization, particularly in its early stages, for phenotypic diversification or adaptive radiation in the group. If I understood correctly, the authors suggest that the chromosomal fusion events and the initial rediploidization observed could be linked to the adaptation of the specialized+highly specialized group to extremely cold environments. However, at present there are no fusion events that appear to be synapomorphic for the specialized+highly specialized clade. Without such shared derived changes, establishing a causal relationship between these fusions and the adaptive evolution of

snow carps remains, in my humble opinion, speculative, and the proposed link seems based solely on a correlation. Addressing this would likely require stronger functional evidence (their RNA-seq DE analysis, for instance, mostly fails to recover genes associated with cold adaptation) together with clearer phylogenetic support. I do not state this as a criticism of the study, but rather as a spark for a stimulating and constructive discussion!

R: We agree that determining causal relationship between rediploidization timing and evolutionary outcomes for adaptation or species radiation remains challenging and the subject of speculation at this stage. Indeed, there is a lack of shared fusions in the clade comprising specialized + highly specialized snow carps. However, this does not preclude lineage-specific rediploidization and/or functional specialization of ohnologs located on chr19-22, which may have been ongoing in the common ancestor to specialized + highly specialized snow carps. Moreover, the highly specialized clade shares a range of ancestral fusions that occurred in their common ancestor, which may have promoted adaptive outcomes in this group. We agree that much deeper functional analyses will be required in the future to convincingly link rediploidization to specific evolutionary outcomes. However, compared to classical models of WGD and evolution, our model of asynchronous rediploidization provides a major conceptual advance in being able to precisely test hypotheses - resolved to specific phylogenetic nodes and individual genes - on the role of rediploidization on such evolutionary outcomes.

In an attempt to strengthen our inferences in this area, we regenerated RNA-seq data from five tissues with six biological replicates for a cold-induction experiment (22℃ *vs.* 5℃), to investigate the potential role of ohnologs in adaptation to cold environments (Fig. 5d and Extended Data Fig. 6b-d). The results showed statistical enrichment of ohnologs among DEGs in the liver and heart (odds ratios = 1.23 and 1.21, respectively), whereas no enrichment was detected in the other tissues (Supplementary Table 13). This pattern indicates that ohnologs and tetrasomic genes both contribute to cold adaptation, although ohnologs may exhibit a slightly more pronounced response under certain conditions. We consider these findings to be suggestive, but still insufficient to support a definitive conclusion. Moreover, we also note that this point is not central to the main claims of the manuscript. For these reasons, we have decided to tone down conclusions on causal relationships between rediploidization and adaptation throughout the revised manuscript.

I have some other minor comments for the authors listed below:

1) L73, L272: Apparently, and at least after ancient WGD events in both gnathostomes and cyclostomes, specialization is a more common fate for duplicates than neofunctionalization or subfunctionalization. See Marlétaz et al., 2018 (https://doi.org/10.1038/s41586-018-0734-6) and Yu et al., 2024 (reference 9)

R: Agreed – we have included "specialization" and cited the amphioxus paper.

2) L73: add "likely" to the statement "promoting phenotypic innovation" to be in line with previous mentions of the potential of WGD events in generating diversification and facilitating evolution of novelty. The causal link between WGDs and their consequences in phenotype evolution are not so clear as I suggest above (see also Yu et al., 2024, reference 9, for an example about cyclostome evolution).

R: We have added "likely", a fair point.

3) Change "basal group" to "non-specialized group": The use of the term "basal" to define one of the snow carp groups is incorrect. A cladogram of extant species just shows the phylogenetic relationships among them, and as such, it does not tell us if an extant clade is basal or not, but instead which it is the sister group to. In this case, what the authors call the "basal" group is actually the sister (species poor) group of the specialized+highly specialized clade. Calling the Aspiorhynchus+Schizothorax clade a "basal group" implies some connotations of a ladder of progress when reading the tree, when in reality both derived from a common ancestor (which is basal to both of them). I suggest therefore to change all instances of basal, in the text and figures, to the more accurate name of "non-specialized" to refer to the group given the context used by the authors.

R: We fully accept your point, and have changed "basal group" to "non-specialized group" throughout the manuscript/figures, ensuring conceptual accuracy. However, to ensure connection with previous studies that have used "basal", we highlight this in parentheses when first introducing "non-specialized".

4) Legend of Supplementary Fig. 7. Add "each" to the sentence for clarity: "10 chromosomes correspond each to two chromosomes in O. macrolepis".

R: We have added "each" to the sentence, as suggested.

5) Naming the stages of rediploidization from III to I sounds counterintuitive. I think it makes more sense in reverse

6) R: We previously used “three stages” because different chromosomes have different ages of chromosomal fusions, thus varying degree of rediploidization. In the revised manuscript, we used the term “waves” instead to refer to varying age of rediploidization rather to imply that rediploidization necessarily has to go through these three (consecutive) stages. Now Stage I, II and III has been changed to Wave1, Wave2 and Wave3, respectively. We hope that this now better describes the observed patterns without verbally implying a mechanism.

6) There is a typo in Fig. 2e, it should be 14 instead of 41.
R: typo corrected, thank you for spotting this.

7) x axis labels for Fig. 4e seems incorrect on the disomic chromosomes?
R: typo corrected, thank you for spotting this.

8) Figure 6 legend: e and d are inverted: d represents a single WGD for snow carps, and e multiple WGDs.
R: These typos have been fixed – thank you again for highlighting these typos.

Nature manuscript: 2025-10-26285B

Referees' comments:

Referee #1 made no comments to the authors.

Referee #2 (Remarks to the Author):

The authors have addressed my comments and provided new data and validations, which satisfactory address my questions on WGD timing and chromosomal-fusion history. What I still strongly recommend to adjust is the discussion of the previous study that claims multiple rounds of polyploidization. I think the current wording is unnecessarily confrontational in the sentence "This strongly refutes a recent study that concluded the existence of multiple independent...". I think this should be changed to rather highlight the actual qualitative difference between the two studies, i.e. the chromosomal-scale data and comparisons.

R: Thank you for your suggestion. We have now avoided the confrontational wording.

I have one last suggestion: in figure 2 (and maybe others like EDF8), I suggest to color the homology lines by chromosomal identity of unfused chromosomes (19 one color, 22 another color etc). This will allow to see if the fused chromosomes have undergone any mixing through translocations across the centromere, which would further solidify their shared derived (and irreversible) state. See for example reference 56 for a few examples for the ancient vertebrate WGDs and the fusion-with-mixing concept and its implications. In the alternative, if these fusions are not majorly mixed yet across the centromere (as I would naively expect given the divergence time), then the fusions could in theory revert back and therefore its good to show several such shared fusions, as the authors do. In any case, the reference 56 is probably good to include in any discussion of the results that suggest impact of chromosomal fusions on rediploidizations after vertebrate WGDs.

R: Thank you for your suggestion. For the fused quartets, we have now used two colors to label the homology lines (EDF2). Yes, as you expected, these chromosomal fusions are too young to have exhibited mixing. According to suggestion, Ref. 56 has been cited in the discussion (Ref. 39).

Referee #3 (Remarks to the Author):

I am pleased to see that the authors found my suggestions, as well as those of other reviewers helpful. I appreciate the additional effort invested in strengthening the manuscript, particularly providing a haplotype resolved genome assembly for a second carp species, and extending their transcriptomics analyses.

As I mentioned previously, I think this is an impressive piece of work that represents a significant advance in our understanding of the genome evolutionary processes following whole genome duplication events.

R: Thank you again for your positive comments.
